# Epoxy–Amine Route to Tough and Degradable Aromatic Polyester Thermosets

**DOI:** 10.1021/acsapm.5c03377

**Published:** 2025-11-26

**Authors:** Jeffrey Aguinaga, Richard C. Ferguson, Michael Blanton, Windfield S. Swetman, James W. Rawlins, Tristan D. Clemons, Travis Thornell, Derek L. Patton

**Affiliations:** † School of Polymer Science and Engineering, 5104 University of Southern Mississippi, Hattiesburg, Mississippi 39406, United States; ‡ Geotechnical and Structures Laboratory, Engineer Research and Development Center, 199011 US Army Corps of Engineers, Vicksburg, Mississippi 39180, United States

**Keywords:** polyester thermosets, epoxy−amine, aromatic ester, toughness, glycolysis

## Abstract

We report polyester thermosets from diepoxide and diamine monomers that contain aromatic ester linkages and short aliphatic spacers between the phenylene units. The influences of aromatic substitution pattern, spacer length, and ester content were studied. An increase in the spacer length or in the level of meta-substitution depressed glass transition temperature consistently. Tensile and flexural strengths and moduli increased with meta-substitution and ester content and decreased as spacer length increased. Properties of reference thermosets derived from monomeric/oligomeric diglycidyl ether of bisphenol A (DGEBA) and para or meta-substituted aromatic amines were compared, and several parallels in strength and modulus were observed. Consistently higher fracture toughness (2.0–3.4×) and impact resistance (5.1–6.9×) were observed for the polyester thermosets – potentially attributable to enhanced molecular mobility and differences in secondary interactions. Additionally, the presence of ester functionality in every network strand enabled glycolytic degradation under ambient pressure – a potential route for end-of-use processing.

## Graphical abstract

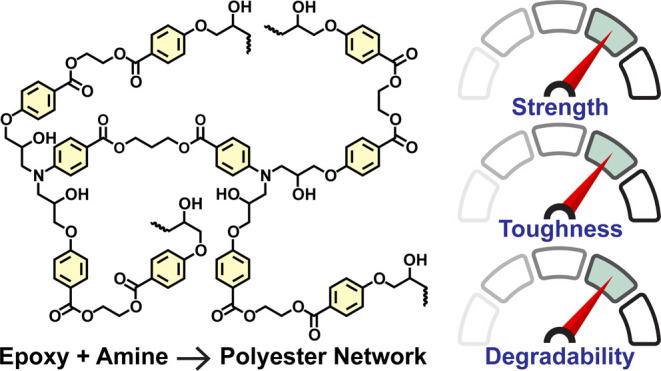

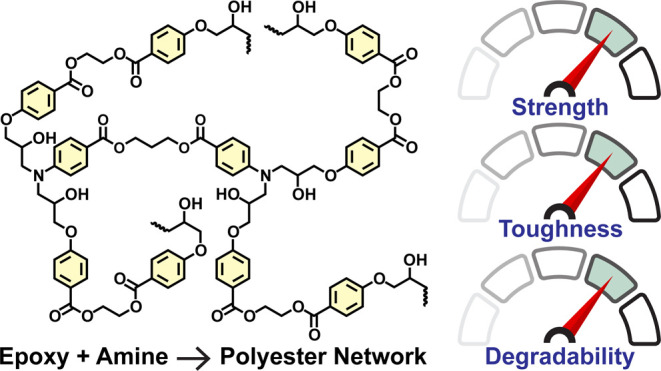

## 1 Introduction

Epoxy thermosets are extensively used in coating, electrical (e.g., power transmission equipment and motor insulation), electronic (e.g., printed circuit boards and component encapsulation), vehicular and energy-production (e.g., aerospace and wind turbine), structural and nonstructural adhesive, and other applications. Common attributes include high thermal, electrical, and corrosion resistance; good mechanical behavior and processability; and lack of condensation byproducts – though properties of epoxy thermosets can vary widely as a function of several factors.^1–6^ Epoxy monomers polymerize via the epoxide (oxirane) group either cationically or anionically through chain growth mechanisms, or via a step-growth mechanism with active-hydrogen compounds (amines, thiols, etc.) as comonomers.^1,2,7,8^ In step-growth polymerization, material properties are controllable through structural variation of the epoxide monomer, the active hydrogen component, or both. DGEBA in monomeric/oligomeric form is the most common epoxide component in terms of volume usage, and constitutes an estimated 75–90% of epoxy systems currently in use.^1,9,10^ While the compact, rigid aromatic structure of DGEBA can contribute to high mechanical properties and thermal/chemical resistance in the resultant thermosets (in particular when an aromatic amine is the comonomer), limited molecular mobility prompts poor resistance to fracture under static or dynamic loading or thermal shock.^2,3,5,11–13^

Various approaches have been developed to augment the fracture toughness of epoxy thermosets – broadly classified into those that rely on secondary phase-separated components and those that focus on the chemical composition and architecture of the thermoset itself.^14^ Examples of approaches that rely on phase-separated components are the reactive or nonreactive incorporation of elastomers and thermoplastics.^11,15,16^ Stress-prompted stretching and void formation in elastomeric domains and local plastic deformation around the interfaces (areas of high stress concentration) can oppose fracture.^16^ Local plastic deformation can also occur around thermoplastic particles, and additional energy dissipation mechanisms related to interference with a propagating crack front can also increase fracture toughness of those systems.^17^ Importantly, less reduction in strength, modulus, and high temperature performance occurs from incorporation of thermoplastics vs elastomers.^11,15,18^ Some thermoplastic and elastomer approaches use reactive-induced phase separation,^19–21^ where the initially high viscosity due to the presence of dissolved high molecular weight polymer can impede processability. When phase separation fails to occur, fracture toughness typically does not increase and modulus can decrease.^22^ Regulation of the phase separation process and of domain size and distribution have also been cited as critical in the use of hyperbranched polymers as toughening agents – where the branched architecture is useful from an energy dissipation standpoint and initial viscosity is lower compared to linear polymers of equivalent molecular weight.^23,24^ Inorganic nanometer-scale fillers can also increase fracture toughness and impact resistance if sufficient dispersion is achieved, and concurrent increases in modulus and strength have been reported.^25,26^ It is also useful to point out that fibrous and micron scale or higher particulate inorganic fillers (typical components in an array of epoxy applications) can increase toughness through fiber bridging, filler debonding, and shear yielding prompted by the difference in epoxy/filler modulus.^27–29^ Micron-scale particulate fillers generally decrease tensile and flexural strength, whereas fibrous fillers can augment both properties.^2^

Modification of the inherent fracture toughness of the thermoset is also possible via modulation of monomer stoichiometry,^12^ functionality,^30^ and/or chemical composition.^31^ Stoichiometry and functionality (in addition to polymerization conditions, etc.) regulate the fraction of elastically active network strands and cross-link density. Although there is generally a strong positive correlation of cross-link density to ambient temperature strength and modulus, relatively high cross-link density can limit molecular mobility through confinement of motions available from chemical structure. Limited molecular mobility can translate to lowered ability to dissipate energy prior to fracture under loading. Levita et al. found an inversely proportional relationship of fracture toughness and cross-link density in evaluation of several DGEBA oligomer/4,4′-diaminodiphenylsulfone (4,4′-DDS) systems.^32^ Tanks et al. found this relationship when linear DGEBA/piperazine-derived strands were integrated into a DGEBA-4,4-diaminodiphenylmethane (4,4′-DDM) system, though the change in cycloaliphatic content relative to aromatic was a potential additional contributor.^30^ Grillet et al. studied thermosets derived from DGEBA and versions of 4,4'-DDM and 4,4'-DDS modified with phenoxy groups.^31^ The additional phenoxy linkages augmented the potential for rotation within the network strands with only a minor decrease in cross-link density. The reported fracture toughness (by critical stress intensity factors (*K*_IC_)) for a phenoxy-modified vs reference network increased by nearly a factor of 1.5.

Another possible approach to increase molecular mobility with minimal reduction of cross-link density relative to typical DGEBA-aromatic diamine thermosets is the integration of aromatic ester linkages into the network structure. These linkages can be incorporated into the interphenylene spans of the epoxy and/or amine comonomer structures. Aromatic ester linkages are rigid compared to aliphatic linkages due to direct attachment to phenyl/phenylene units, yet the rotational barrier remains relatively low. Rotational rearrangements of segments in glassy thermoplastics under loading have been described as substantial contributors to plastic deformability, though the cross-linked architecture inherent to epoxy thermosets resists plastic deformation.^33–35^ Molecular elongation/reorientation are critically connected to plastic deformability,^35^ and the energy dissipated in plastic deformation can augment material toughness. The incorporation of ester groups could also increase energy dissipation ability through augmentation of secondary network structure. Additionally, the esterification process to form the monomers permits the modular introduction of aliphatic spacer groups internal to the rigid phenylene units. Through constriction of the length of the aliphatic spacer, reduction in cross-link density and the accompanying losses in strength and modulus could be minimized.

Kakiuchi and Takei^36^ were the first to report aromatic ester-containing epoxy monomers with aliphatic spacers internal to phenylene groups. Several monomers derived from 4-hydroxybenzoic acid (pHBA) and aliphatic spacers that ranged in length from ethyl to hexyl were reported. While the work demonstrated synthetic feasibility, it did not examine the toughness of the polymers derived from those structures. Although several other researchers have incorporated aromatic ester functionality into epoxy monomers, they have focused mainly on bisphenol A (BPA) replacement and bioderivability.^37–40^ Analogous ester-containing diamines have also been used in epoxy-derived thermoset polymers. For example, Cui et al. used an aliphatic spacer-bridged aromatic ester-containing diamine at additive level (0–1%) to augment molecular flexibility and limit thermal stress accumulation in a thermoset novolac-type epoxy molding compound for electronics application.^5^

In addition to the utility of aromatic ester linkages from a materials property standpoint, these linkages are attractive from a chemical degradability standpoint – a concept that underpins the present study. Efforts to introduce degradability into epoxy thermosets via ester linkages have been widely reported,^41,42^ with many approaches relying on reactions that generate ester linkages in the cure process – particularly epoxy–anhydride and epoxy–acid systems.^6,39,43^ These systems can undergo hydrolytic or alcoholytic cleavage – typically under acidic, basic conditions, or elevated temperature – enabling deconstruction to small-molecule fragments.^42^ A continuing challenge in this area is achieving degradability while maintaining high-performance. As emphasized by Robertson et al.^44^ thermosets capable of hydrolysis or alcoholysis must be sufficiently robust during service yet cleavable under specific postuse conditions. Balancing these opposing design criteria typically requires careful control of how and where degradable motifs are incorporated into the network. In contrast to systems where ester linkages arise only from the curing reaction, embedding aromatic ester groups as phenylene bridging units within both epoxy and amine monomers provides a uniform distribution of cleavable sites. Critically, this monomer-level design approach also enables systematic tailoring of structural variables (spacer length, substitution pattern, ester content) to optimize performance and degradability.

Building on this design concept, we report an array of aromatic ester–containing diepoxide and diamine monomers and corresponding polyester thermosets derived from epoxy-amine reactions. The incorporation of short aliphatic spacers between aromatic ester units balances molecular mobility and network rigidity to tailor the plastic deformation response and toughness. To demonstrate modularity within this platform, we examined the effects of para versus meta substitution, spacer length, and ester content on network properties, with particular emphasis on strength, fracture toughness, impact resistance, and adhesion relative to conventional DGEBA–aromatic diamine systems. Additionally, we also evaluated degradation by alcoholysis as an end-of-use processing strategy.

## 2 Results and Discussion

### 2.1 Synthesis of Ester-Bridged Diepoxide and Diamine Monomers

To enable the synthesis of several structurally related aromatic ester diepoxide and diamine monomers, asymmetric monoester and symmetric diester bisphenol and diarylnitro precursors were prepared via transesterification. Asymmetric diaryl precursors bridged by a single ester were synthesized from either 4-(2-hydroxyethyl)­phenol (tyrosol) or 4-nitrophenethyl alcohol using a near-stoichiometric ratio of para-substituted methyl benzoate in a single-stage reaction (Figure 1a). In contrast, syntheses of symmetric diaryl precursors bridged by two esters generally followed a two-stage transesterification approach: an initial stage employed a 4:1 molar excess of alcohol to benzoate functionality to preferentially generate a monocondensation adduct. In the second stage, free diol was removed under reduced pressure, and additional diol was reactively distilled during conversion of the monocondensation adduct to the dicondensation adduct (Figure 1b–c). In all cases, compounds were isolated via solvent rinses to remove the dibutyltin dilaurate (DBTDL) transesterification catalyst and used without additional purification. ^1^H and ^13^C nuclear magnetic resonance (NMR) spectra for all bisphenol and dinitro compounds are shown in Figures S1–S16. Yields ranged from 78 to 87% for bisphenol compounds and 80–87% for aryl dinitro compounds.

**Figure 1:**
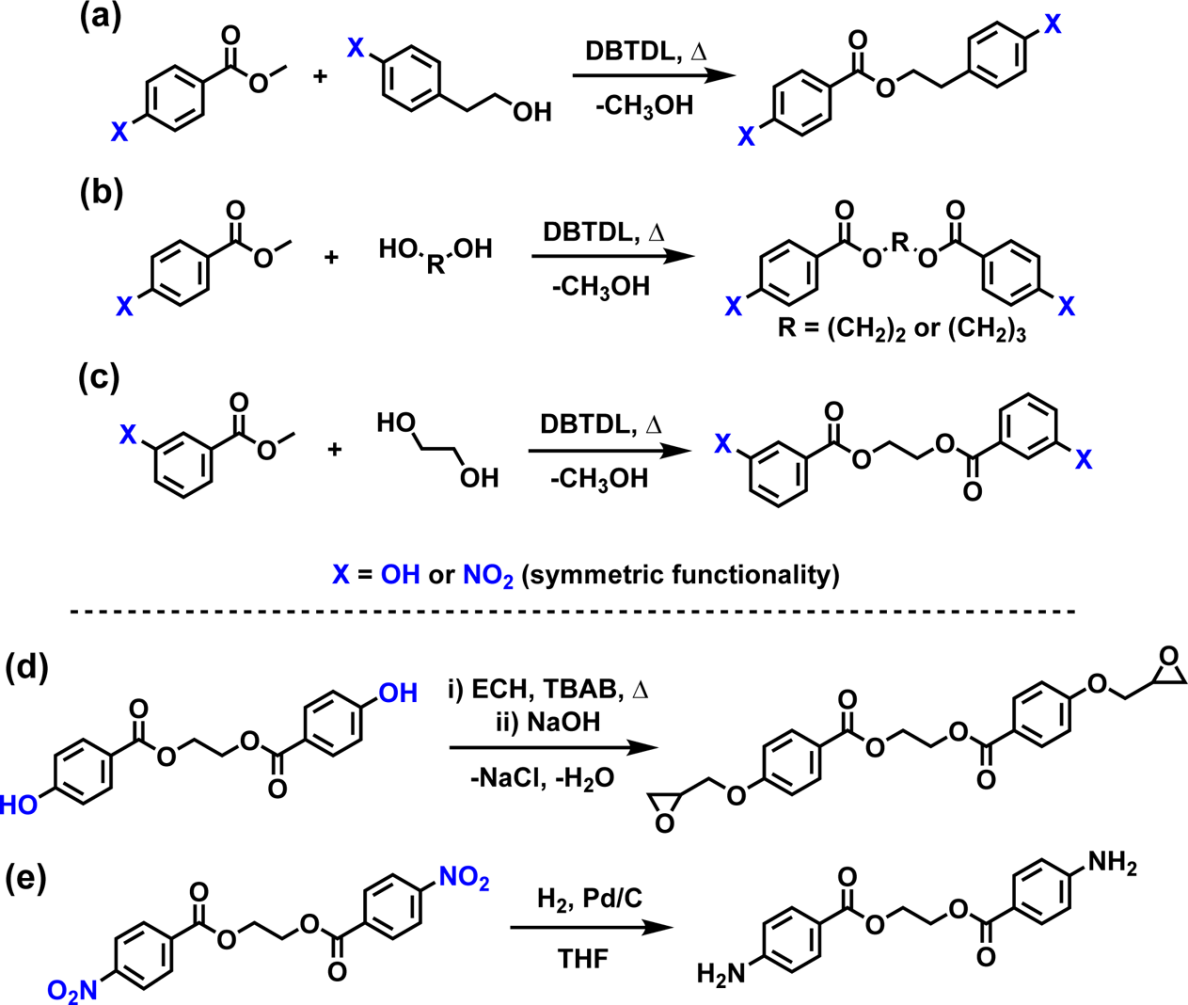
Synthetic routes to representative precursors and monomers: (a–c) DBTDL-catalyzed transesterification yielding precursors with bisphenol or dinitro functionality; (d) diepoxide monomers prepared from bisphenol precursors via ECH; (e) diamine monomers obtained by catalytic hydrogenation of dinitro precursors.

The Fischer esterification route described in US Patent US 2016/0177028 Al^45^ was also investigated for the production of certain bisphenols. Although that approach permitted synthesis of symmetric bisphenols, production of the asymmetric bisphenol was problematic – potentially due to decomposition of the alcohol reactant in the strongly acidic conditions. In contrast, we were able to prepare all bisphenols via the bulk transesterification route described. For ester-containing bisphenol syntheses, reaction temperatures were generally limited to 170–180 °C to mitigate potential transesterification with the phenol functionality. It was also found that the use of ethyl p-hydroxy benzoate as opposed to methyl p-hydroxy benzoate could limit phenolic reaction – possibly due to the lower reactivity of ethyl p-hydroxy benzoate. The absence of this side reaction in the diarylnitro syntheses allowed use of higher temperatures (180–200 °C, up to 240 °C near completion) to drive conversion.

All bisphenol precursors were converted to diepoxide monomers using a two-stage epichlorohydrin (ECH)/base procedure adapted from that described by Fang et al.^46^ This approach is a well-established route for synthesizing aromatic glycidyl ethers, providing reproducible conversion and high product purity without extensive purification. A representative epoxidation of a symmetric diester bisphenol is shown in Figure 1d. In this process, ECH functioned as a reactant and a solvent. A 10:1 molar ratio of ECH to phenol was used, and tetra-butylammonium bromide (TBAB) was used as a coupling catalyst in the initial stage and a phase transfer catalyst in the biphasic second stage of the epoxidation reaction. The role of a quaternary ammonium salt such as TBAB in ECH ring-opening, coupling to phenol, and chlorohydrin ring closure to form glycidyl aryl ether linkages is described mechanistically in US2943096A.^47^ NaOH was gradually added as an aqueous solution at near ambient temperature. Despite the potential for ester hydrolysis in the alkaline second stage of the epoxidation process, crude ^1^H NMR spectra indicated minimal hydrolysis (0–4%), based on aromatic-to-ester methylene integral ratios. Crude diepoxide monomers were washed with water to remove NaOH, dried, and concentrated. Monomers were dissolved in dichloromethane and run through silica plugs to remove TBAB. No further purification was performed. ^1^H and ^13^C NMR spectra for all diepoxide monomers are shown in Figures S17–S24. Yields ranged from 75 to 82%.

Aryl dinitro compounds were reduced to diamines via Pd/C-catalyzed hydrogenation in tetrahydrofuran, as shown in the synthetic scheme in Figure 1e. To minimize ester reduction, the reaction temperature was maintained at ambient to 30 °C. Pd/C was removed by filtration through a silica plug, and no further purification was performed. ^1^H and ^13^C NMR spectra for all diamines are shown in Figures S25–S32. Yields for the diamines ranged from 92 to 97%.

In addition to potential side reactions in ester-containing monomer synthesis, amidation between amine and ester groups could, in principle, occur during network formation. To evaluate the occurrence of this side reaction, a model reaction was conducted using non-network forming monoepoxide (ethyl-4-glycidyloxybenzoate) and monoamine (ethyl-4-aminobenzoate/benzocaine) reactants, chosen for their structural similarity to the diepoxide and diamine monomers in this work. ^1^H NMR spectra of the reactants are shown in Figures S33–S34, and a scheme of the potential side reaction is shown in Figure S35. The reaction was performed under the same thermal conditions as used for formation of 4 of 6 of the polyester networks (network structures in Figure 2): 120 °C for 3 h followed by a ramp to 200 °C over 2 h and an isothermal hold at 200 °C for an additional 2 h. The ^1^H NMR spectrum of the product (Figure S36) was consistent with the expected epoxy–amine adduct. Specifically, the terminal methyl protons of the triethyl ester structure appeared at 1.27 and 1.30 ppm. The integral ratio of these peaks to the aromatic protons (7.91, 7.71, 7.06, and 6.83 ppm) was 9.1:11.8, closely matching the theoretical value of 9:12. If amidation had occurred, this ratio would decrease due to cleavage of ester groups. Further, ethanol (a byproduct of amidation) does not contribute to the observed methyl signals, as the reported chemical shift for the methyl protons of ethanol is 1.06 ppm in the same solvent.^48^ This suggests that under the applied conditions, the monomers do not undergo appreciable amidation.

**Figure 2:**
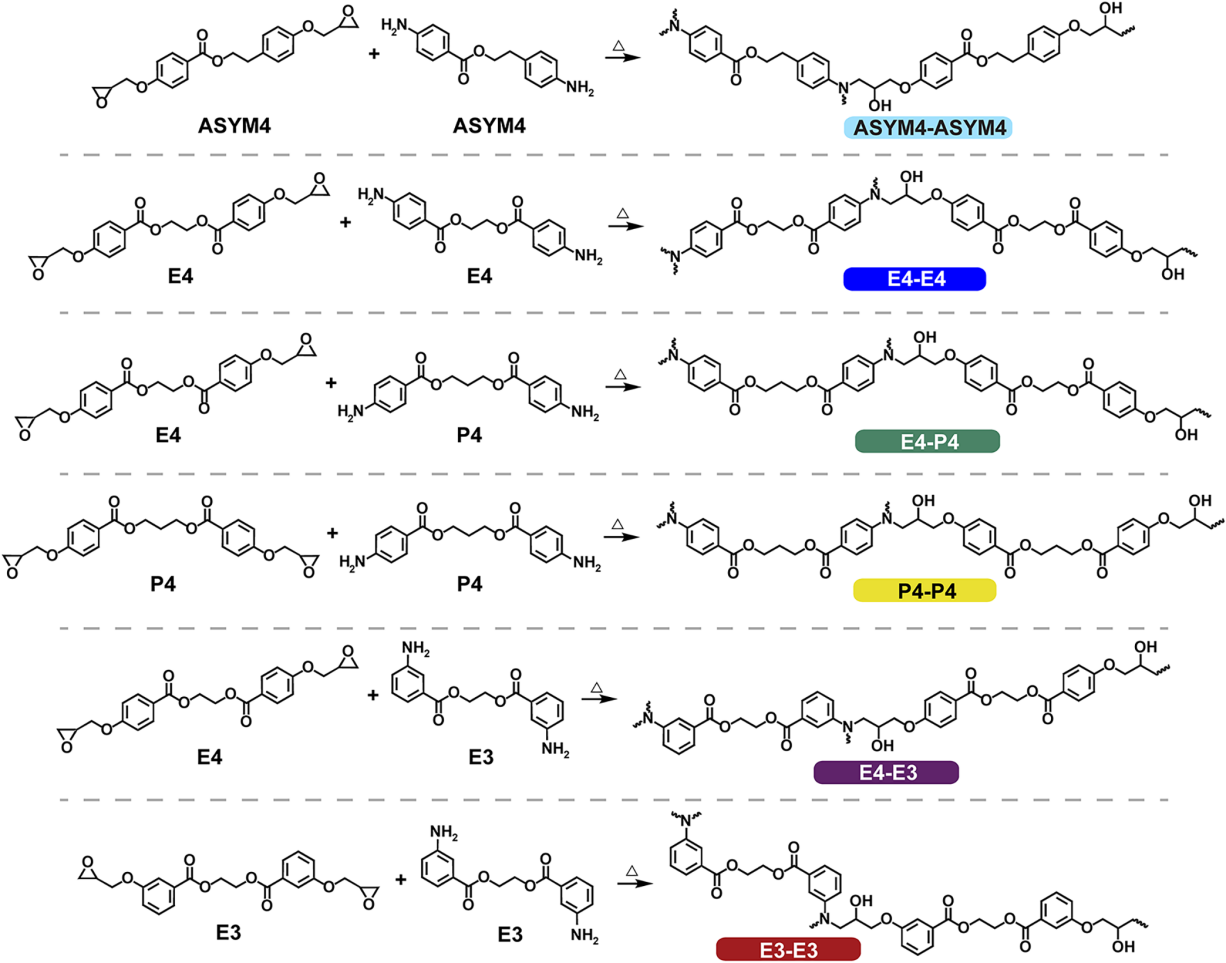
Overview of polyester networks derived from epoxy and amine components.

### 2.2 Synthesis of Polymer Thermosets

To evaluate structure–property relationships in polymer networks formed from ester-containing monomers, six polyester thermosets (Figure 2) were synthesized from the diepoxide and diamine components using a nominal 1:1 stoichiometry of epoxide to amine hydrogen. Abbreviations for polyester networks reflect the structure of the diepoxide and diamine monomers: 3 and 4 indicate meta- and para-substitution, “E” and “P” indicate ethyl or propyl spacers between the aromatic ester groups of symmetric monomer structures, and “ASYM” refers to asymmetry – as in the asymmetric diepoxide and diamine monomers bridged by single ester groups. Thermosets derived from DGEBA monomer/oligomer and 4,4′- DDM, 4,4′-DDS, or 3,3′-diaminodiphenyl sulfone (3,3′-DDS) were included as industrially relevant aromatic epoxy-amine reference systems.

Single ramp differential scanning calorimetry (DSC) experiments were used to determine cure temperature profiles for each system. Polymerization exotherm onsets, peak maxima and activation energies (*E*_a_) determined by the Kissinger method^49^ are summarized in Table 1. *E*_a_ values for the polyester networks spanned 54.6–63.6 kJ mol^–1^, falling between those of the DGEBA-4,4′-DDM (53.5 kJ mol^–1^) and DGEBA-4,4′-DDS (65.8 kJ mol^–1^) systems, underscoring their intermediate reactivity profiles. *E*_a_ values varied primarily with the nucleophilicity of the amine component. 4,4′-DDM lacks electron withdrawing substituents; this prompted the lowest peak exotherm and *E*_a_ for DGEBA-4,4′-DDM among all systems, consistent with a relatively nucleophilic amine. Systems that used symmetric diamines with ester substitution para to the amine groups (E4-E4, E4-P4, P4–P4) exhibited higher peak exotherm temperatures and activation energies due to the electron withdrawal ability of the para-positioned ester. For reference, *E*_a_ for the reaction of DGEBA and P4 has been reported as 63.7 kJ/mol – close to the values obtained in this work for the E4-P4 and P4–P4 systems (61.9 and 62.8 kJ/mol, respectively).^50^ In the DGEBA-4,4′-DDS reference system where the amine is para to a sulfone group – a stronger electron withdrawing group than an ester – a marginally higher peak exotherm and *E*_a_ were observed, consistent with diminished nucleophilicity of 4,4′-DDS relative to the para-ester substituted amines. Comparative DSC traces for network formation of representative polyester system E4-P4 and for DGEBA-4,4′-DDS that include peak temperatures and enthalpies are shown in Figure S37.

**Table 1:** Polymerization Data Summary

network	peak exotherm temperature (°C)^a^	exotherm onset temperature (°C)^a^	*E*_a_ (kJ/mol) via Kissinger method
DGEBA-4,4′-DDM	114	96	53.5
DGEBA-4,4′-DDS	171	141	65.8
DGEBA-3,3′-DDS	156	130	61.9
ASYM4-ASYM4	129	103	54.6
E4-E4	169	144	63.6
E4-P4	164	133	61.9
P4-P4	166	137	62.8
E4-E3	147	118	59.5
E3-E3	147	115	57.8

a Determined at 1 °C per minute ramp rate.

The meta-substitution in 3,3′-DDS prevents resonance delocalization of the nitrogen lone pair; this increases the nucleophilicity of the amine relative to the 4,4′-analog.^51^ Accordingly, DGEBA-3,3′-DDS exhibited a lower peak temperature and *E*_a_. Similarly, polyester systems with meta-substituted ester groups in the diamine component (E4-E3, E3-E3) exhibited lower exotherm temperatures and activation energies compared to their para-substituted counterparts. Among the ester-containing systems, ASYM4-ASYM4 had the lowest peak temperature and *E*_a_. This diamine is structurally asymmetric as it features one amine group para to an ester and the other para to a nonelectron-withdrawing aliphatic span; asymmetric reactivity in this system is prompted by differential amine nucleophilicity.

Polymerizations were conducted in bulk without external catalyst. Solubility was achieved in all cases, as was qualitatively assessed by optical clarity. The temperature where a monomer solution formed correlated with the melting point (mp) of the diamine component. Systems that used ASYM4, P4, or E3 diamines (mp = 127–133 °C) formed solutions at ≤ 120 °C. In contrast, the system that contained the E4 diamine (mp = 214 °C) achieved full dissolution at >150 °C. Elevated temperature could impede controlled network formation, as there is the potential for higher *E*_a_ secondary amine reaction to cause branches/cross-links prior to substantial linear chain development (from primary amine reaction) – and this could prompt a higher level of network defects/heterogeneity.^52,53^ To minimize time at elevated temperature, the E4 diamine was pulverized to increase surface area; this enabled complete dissolution at ca. 160 °C in less than 10 min. From this point it was possible to lower the temperature for the E4-E4 polymerization to proceed under relative control. Due to the higher initial temperature, the cure protocol for the E4-E4 network differed from the 120–200 °C profile used for the other symmetric polyester systems (see Section 4 for details).

The extents of cure for the polyester networks were assessed via DSC from the ratio of initial to residual polymerization enthalpy following completion of the prescribed cure protocols (Table S1). For the symmetric polyester systems enthalpy data indicated 95.0–96.6% extent of cure, whereas the asymmetric system reached a lower 93.8% extent of cure. By the same approach the DGEBA-4,4′-DDS and 3,3′-DDS reference systems reached 95.4 and 96.0% extent of cure (respectively), and the DGEBA-4,4′-DDM reference system reached 98.1%. DSC traces for extent of cure evaluation of representative polyester system E4-P4 are shown in Figure 3a–b. NIR spectroscopy was used to monitor functional group absorbances over the course of the E4-P4 polymerization, and the Beer–Lambert law was applied to determine concentrations (Figure 3c–d).^53,54^ Quantification of the NIR spectra confirmed that epoxide, primary amine, and secondary amine groups were depleted to baseline levels by the end of the cure – in rough agreement with the DSC results. NIR spectra for all other polyester and nonester containing reference systems taken throughout polymerization are shown in Figures S38–47.

**Figure 3:**
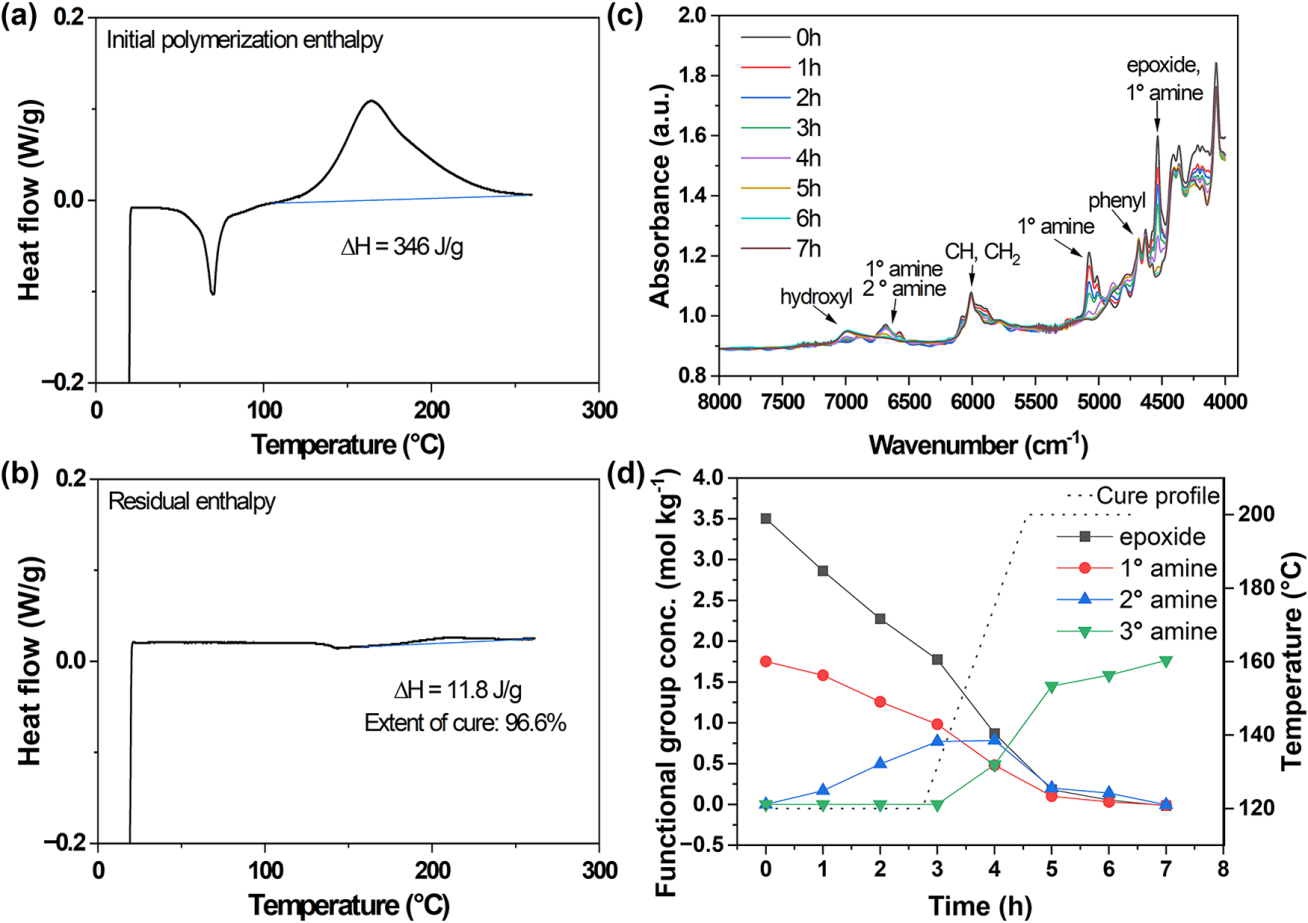
(a) Initial and (b) residual polymerization enthalpy, (c) overlaid near-infrared (NIR) spectra and (d) functional group concentrations throughout the cure protocol for representative polyester system E4-P4.

### 2.3 Properties of Polyester Thermosets

#### 2.3.1 Thermal Stability

The thermal stabilities of the polyester networks were evaluated via thermogravimetric analysis (TGA) using a 10 °C/min ramp rate under N_2_. Weight % vs temperature traces are shown in Figure 4a, with quantification summarized in Table 2. The 5% weight loss temperature (T_d,5_) for the ASYM4-ASYM4 network was 370 °C, and those for the symmetric networks ranged from 378 to 382 °C. Despite the high concentration of ester functionality, the thermal stabilities of polyester networks were only marginally lower than that of the DGEBA-type networks (387–407 °C). Although ester thermolysis is a potential concern, primary ester groups in epoxy thermosets have been reported as stable up to ca. 340 °C.^55,56^ Epoxy networks that contain a higher percentage of oxygen linkages have increased susceptibility to chain scission and cross-linking events that promote char formation,^57^ so the presence of ester functionality in general could partially explain the higher average residue at 800 °C observed for the polyester networks relative to the DGEBA type networks. Among the polyester networks, char yield increased with meta substitution (E4-E4 < E4-E3 < E3-E3). Char yield also increased as the aliphatic spacer length decreased among completely para-substituted networks (P4-P4 < E4-P4 < E4-E4), consistent with the aromatic content of those structures.

**Figure 4:**
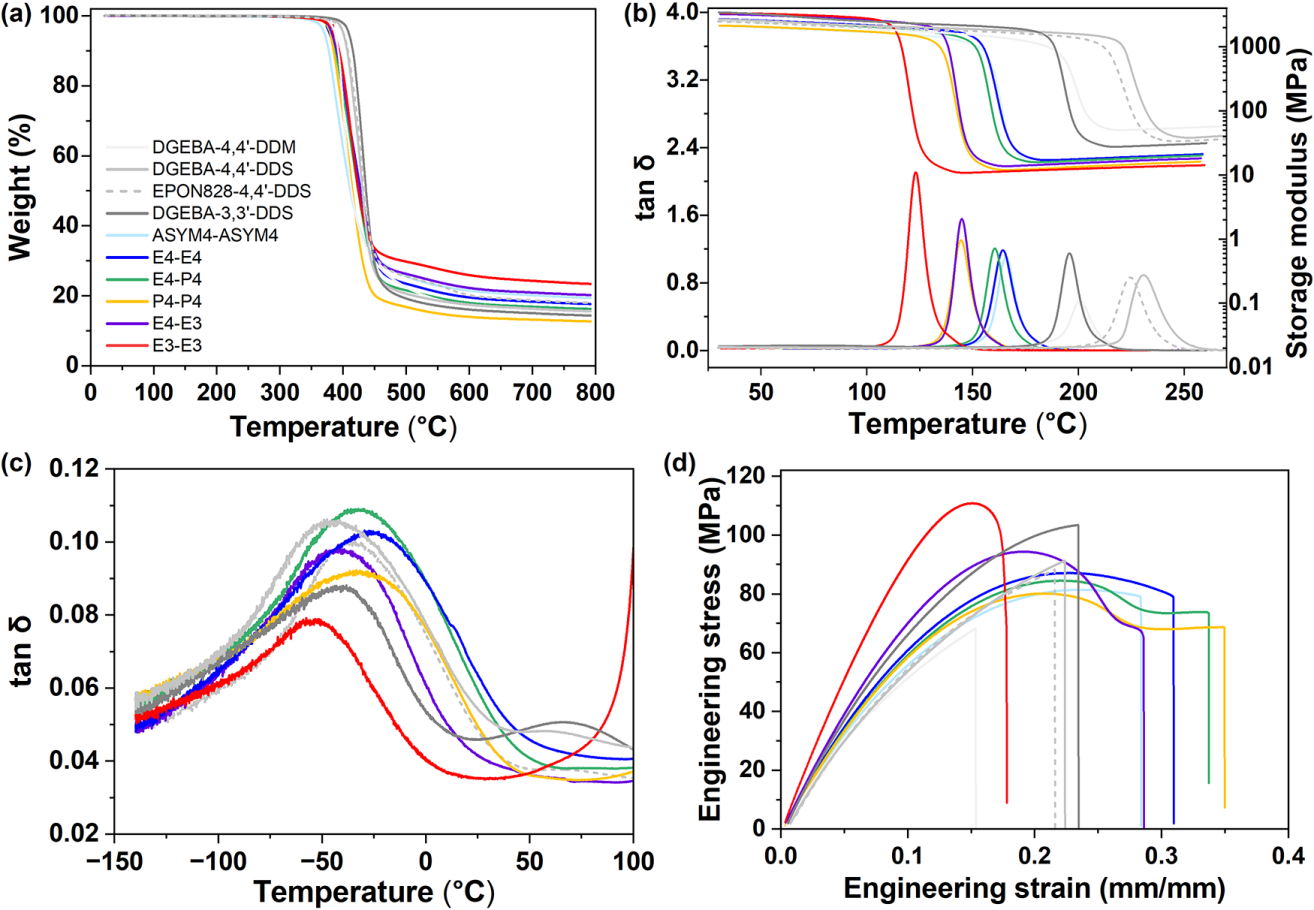
Representative (a) thermogravimetric, (b) storage modulus and tan δ, (c) subambient tan δ and (d) tensile stress/strain traces for polyester and reference networks.

**Table 2:** Network Thermal Stability, Thermomechanical Property, and Crosslink Density Summary

network	*T*_d5_ (°C)	residue at 800 °C (%)	peak tan δ (°C)	peak tan δ fwhh^a^ (°C)	M_c, theoretical_ ^b^ (g/mol)	υ_theoretical maximum_ ^c^ (mol/cm^3^)
DGEBA-4,4′-DDM	387 ± 0.7	16.1 ± 1.1	200 ± 0.5	12.3 ± 0.3	294	0.00272
DGEBA-4,4′-DDS	400 ± 0.0	15.7 ± 0.1	231 ± 0.6	15.5 ± 0.2	310	0.00267
Epon 828-4,4′-DDS	401 ± 0.0	17.6 ± 0.6	225 ± 0.2	14.4 ± 0.1	333	0.00249
DGEBA-3,3′-DDS	407 ± 2.0	14.7 ± 0.4	196 ± 0.2	9.8 ± 0.1	310	0.00267
ASYM4-ASYM4	370 ± 0.2	19.4 ± 0.1	163 ± 2.6	10.9 ± 1.0	333	0.00255
E4-E4	378 ± 0.7	17.8 ± 0.2	164 ± 0.6	11.0 ± 0.2	377	0.00232
E4-P4	381 ± 0.6	15.6 ± 1.0	160 ± 0.3	9.8 ± 0.0	382	0.00228
P4-P4	381 ± 1.2	12.6 ± 0.2	143 ± 1.1	10.2 ± 0.1	391	0.00222
E4-E3	382 ± 0.6	20.2 ± 0.0	144 ± 0.5	8.8 ± 0.0	377	0.00233
E3-E3	380 ± 3.9	21.0 ± 2.5	122 ± 1.0	9.4 ± 0.4	377	0.00233

a fwhh = full width at half height.

b M_c_ = theoretical molecular weight between cross-links

c υ_theoretical maximum_ = theoretical maximum cross-link density

#### 2.3.2 Thermomechanical and Mechanical Properties

Thermomechanical properties of the networks were probed by dynamic mechanical analysis (DMA), with representative plots supplied in Figure 4b (temperature range = ambient to 260–300 °C) and Figure 4c (−140 °C to +100 °C). Quantitative data are summarized in Tables 2 and S2, respectively. Primary relaxation peaks associated with the glass transition temperature (*T*_g_) for the polyester networks occurred at 122–164 °C vs 196–231 °C for the DGEBA reference networks. The difference in *T*_g_ is attributable in part to the increased molecular mobility introduced by the ester linkages and aliphatic spacers in the phenylene bridges of the polyester networks. An additional contributor is the difference in cross-link density (υ). Theoretical maximum cross-link densities for all networks (included in Table 2) were calculated from average strand molar masses (molar masses between cross-link points) and densities obtained via Archimedes’ principle. Average strand molar masses were weighted as 2/3 of network strands are diepoxide-derived and 1/3 are diamine-derived, and conversion to cross-link densities was completed as described by Levita et al. and Chang et al.^32,58^ Details related to the calculation are supplied in Table S3.

The theoretical cross-link densities of networks derived from DGEBA monomers (0.00267–0.00272 mol/cm^3^) are higher than those of the polyester networks (0.00222–0.00233 mol/cm^3^ for symmetric networks, 0.00255 mol/cm^3^ for the asymmetric network). To partially normalize to theoretical cross-link density, a network derived from Epon 828 (monomeric/oligomeric DGEBA – a standard liquid epoxy resin) and 4,4′-DDS was evaluated (theoretical cross-link density = 0.00249 mol/cm^3^). Although the cross-link density is lower for the Epon 828 network than the asymmetric (ASYM4-ASYM4) network – *T*_g_ of the ASYM4-ASYM4 network is still substantially lower. In contrast, the *T*_g_ of the ASYM4-ASYM4 network is practically the same as that of the lower-theoretical cross-link density E4-E4 network. This is likely due to the additional aromatic ester linkage present in every network strand of the symmetric E4-E4 network in place of the aliphatic span on one side of the phenylene bridge in the ASYM4-ASYM4 network.

Among the para-substituted symmetric polyester networks, a systematic decrease in *T*_g_ was observed as the aliphatic spacer length increased from ethyl–ethyl (E4-E4) to ethyl–propyl (E4-P4) to propyl–propyl (P4-P4). Probable contributors to this decrease are reduction in cross-link density and increased network strand flexibility.^59^ Additionally, minor dilution of functional groups with linear aliphatic content could reduce the extent of hydrogen bonding.

Another systematic decrease in *T*_g_ was observed as meta substitution increased in the polyester networks (E4-E4 to E4-E3 to E3-E3). Meta-substitution of the diamine in the DGEBA reference networks (3,3′-DDS vs 4,4′-DDS) also depressed *T*_g_, consistent with prior reports.^60^ This effect arises because the asymmetry of the meta linkages increases the number of accessible conformational states (higher conformational entropy). Relative to the para-linked networks, meta-linked networks can explore more conformations above *T*_g_ yet pack more efficiently in the glassy state (reflected in the higher density of 3,3′-DDS vs 4,4′-DDS or E3-E3 vs E4-E4, Table S3).^57,61,62^ Due to the entropic consideration, less thermal energy input is needed to reach the rubbery state for the 3,3′-DDS system vs the 4,4′-DDS system.^60^ The influence of the isomeric substitution on storage modulus is also evident (Figure 4b). The glassy modulus consistently increases with meta substitution due to tighter chain packing and reduced free volume (i.e., DGEBA-3,3′-DDS > DGEBA-4,4′-DDS and E3-E3 > E4-E3 > E4-E4) and the rubbery plateau modulus consistently decreases due in part to greater conformational freedom above *T*_g_.

As secondary (sub-*T*_g_) mechanical relaxations could contribute to plastic deformation response in the glassy state,^35^ we extended the analysis range to subambient temperature (−140 °C). These glassy-state relaxations arise from short-range molecular motions, such as rotations of pendant groups, phenyl ring flips, or crankshaft-like movements of short backbone segments. In epoxy-amine thermosets, a relaxation typically reported near −60 °C has been attributed to crankshaft-like motion of 2-hydroxypropyl linkages formed during polymerization.^57,60,63,64^ For the networks studied here, the secondary relaxation peaks appeared as broad, low-intensity tan δ peaks (Figure 4c) and likely consist of several distinct types of localized motions. Despite the overlapped contributions, several trends were apparent as a function of structure. For example, as the para-phenylene content decreased from E4-E4 to E4-E3 to E3-E3, the integrated area of the tan δ peak decreased from 5.83 to 4.63 and 2.28, respectively. Additionally, a progressive shift of the peak maximum to lower temperature occurred (−29.1, −44.1, and −54.1 °C respectively). This attenuation reflects the lack of rotational freedom of meta-phenylene groups vs para-phenylene.^61,64^ In the fully meta-substituted E3-E3 network, phenylene rotation would no longer contribute to the integral. In comparison of the DGEBA-4,4′-DDS and DGEBA-3,3′-DDS, the tan δ area decreased from 4.95 to 3.59 and the peak temperatures shifted from −43.9 to −54.1 °C, respectively. For the polyester systems, ester rotation could contribute to the observed secondary relaxations. Although Escaig et al. did not associate ester rotation with an observed secondary relaxation in unsaturated polyesters (in fact only related secondary relaxation to network defects),^65^ Illers et al. associated ester motion with a secondary loss-modulus peak near −65 °C in poly­(ethylene terephthalate) (PET).^66^ While PET is a thermoplastic, at a localized scale there is analogous connectivity (phenylene-ester-alkyl) to the networks reported herein.

In the para-substituted polyester networks, the secondary relaxation integral also decreased as aliphatic spacer length increased (E4-E4 > E4-P4 > P4-P4; 5.83 to 5.50 to 4.63). This is probably attributable to decreases in the concentrations of p-phenylene, 2-hydroxypropyl, and ester groups as linear aliphatic content increases. Although the thermally overlapped motions that likely contributed to the observed peaks were not isolated, the cumulative integral for each system is still potentially useful for comparison of local mobility and is related to fracture toughness in a later section.

Mechanical properties were evaluated at ambient temperature – approximately 100–140 °C below *T*_g_ for the polyester networks and 170–210 °C below *T*_g_ for the reference networks. At test temperature, secondary relaxations are mainly thermally accessible (refer to Figure 4c) and could contribute to mechanical response. A summary of tensile and flexural property data for all polymer networks is supplied in Table 3 and representative tensile and flexural curves are shown in Figures 4d and S48, respectively. The polyester networks generally exhibited ductile failure in tension, with strains at yield that ranged from 0.148 to 0.240 mm/mm, whereas nonester containing reference networks did not yield. Mechanical yield has been described as governed by *T*_g_ and cohesive energy density.^67,68^ *T*_g_ in all cases for polyester networks is lower than for nonester containing reference networks, yet the addition of polar functionality like ester groups could augment cohesive energy density.^69^

**Table 3:** Summary of Ambient Temperature Mechanical Properties

network	tensile strength (MPa)	tensile strain at yield (mm/mm)	tensile strain at break (mm/mm)	flexural strength (MPa)	flexural modulus (MPa)	flexural toughness (MJ/m^3^)
DGEBA-4,4-DDM	70.2 ± 2.2	N/A	0.164 ± 0.013	117 ± 7	2786 ± 58	3.54 ± 0.67
DGEBA-4,4-DDS	85.0 ± 3.3	N/A	0.187 ± 0.019	146 ± 8	3006 ± 66	4.81 ± 0.72
Epon 828–4,4-DDS	87.8 ± 3.3	N/A	0.209 ± 0.023	141 ± 1	3292 ± 83	3.68 ± 0.28
DGEBA-3,3-DDS	107 ± 1.7	N/A	0.222 ± 0.007	167 ± 11	3614 ± 94	5.01 ± 0.82
ASYM4-ASYM4	81.2 ± 0.1	0.240 ± 0.001	0.298 ± 0.019	134 ± 3	2702 ± 40	9.42 ± 0.15
E4-E4	85.6 ± 0.9	0.233 ± 0.001	0.306 ± 0.012	149 ± 3	3004 ± 33	10.47 ± 0.21
E4-P4	83.3 ± 1.0	0.220 ± 0.004	0.327 ± 0.040	144 ± 6	2719 ± 43	10.06 ± 0.48
P4-P4	79.8 ± 0.5	0.209 ± 0.001	0.309 ± 0.021	133 ± 3	2567 ± 69	9.22 ± 0.20
E4-E3	94.1 ± 0.2	0.189 ± 0.002	0.283 ± 0.015	172 ± 2	3416 ± 70	12.01 ± 0.15
E3-E3	112 ± 0.5	0.148 ± 0.003	0.169 ± 0.008	188 ± 9	4154 ± 130	10.05 ± 2.06

Among completely para-substituted polyester networks, strain at yield values decreased consistently with decreased theoretical cross-link density and decreased *T*_g_ (i.e., ASYM4-ASYM4 > E4-E4 > E4-P4 > P4-P4). Strain at yield also decreased progressively as meta-substituted content increased (i.e., E4-E4 > E4-E3 > E3-E3). The tighter packing and restricted rotational freedom of meta-substituted linkages can contribute to the accumulation of stress prior to yield. Tensile and flexural strengths increased with meta substitution across both polyester (E4-E4 < E4-E3 < E3-E3) and reference (DGEBA-4,4′-DDS < 3,3′-DDS) networks. The tensile stress at yield of the fully para substituted E4-E4 network closely matched the stress at break of the fully para substituted DGEBA-4,4′-DDS benchmark, while the partially meta substituted E4-E3 network approached the strength of the partially meta substituted DGEBA-3,3′-DDS benchmark. The fully meta-substituted E3-E3 network exhibited the highest tensile and flexural strengths of any evaluated. Despite a noticeable reduction in strain at break for the E3-E3 network relative to E4-E3 and E4-E4, the material still yielded prior to failure, consistent with the ductile character of the epoxy/amine-derived polyesters.

Among fully para-substituted polyester networks, an increase in the spacer length (i.e., from E4-E4 to E4-P4 to P4-P4) led to progressive decreases in both tensile and flexural strength, and flexural moduli within that group also decreased as spacer length increased. Moduli among all polyester networks ranged from 2.6 to 4.2 GPa (vs 2.8–3.6 GPa for reference networks). A consistent modulus increase with meta substitution was observed (i.e., from E4-E4 to E4-E3 to E3-E3, and DGEBA-4,4′-DDS to DGEBA-3,3′-DDS). Flexural toughness, calculated from the area under the stress/strain curve, was higher (9.2–12.0 MJ-m^3^) for the polyester networks compared to the DGEBA reference networks (3.5–5.0 MJ-m^3^). The pronounced ductility of the polyester networks delayed or prevented failure within the 0.10 mm/mm strain window and prompted higher comparative flexural toughness.

#### 2.3.3 Fracture Toughness and Impact Resistance

Resistance to fracture was evaluated under plane-strain conditions in mode I loading. Fracture toughness (*K*_IC_) was determined using single-edge notched bend (SENB) specimens. Results for symmetric polyester and DGEBA-DDS networks are shown in Figure 5a. To examine the influence of cross-link density, a DGEBA-type network of lower cross-link density than any of the polyester networks derived from Epon 834 and 4,4′-DDS was included (υ_theoretical maximum_ = 0.00199 mol/cm^3^, *T*_g_ = 193 ± 0.2 °C, tensile strength = 89.9 MPa). *K*_IC_ increased as cross-link density decreased among the DGEBA/Epon-4,4′-DDS series.

**Figure 5:**
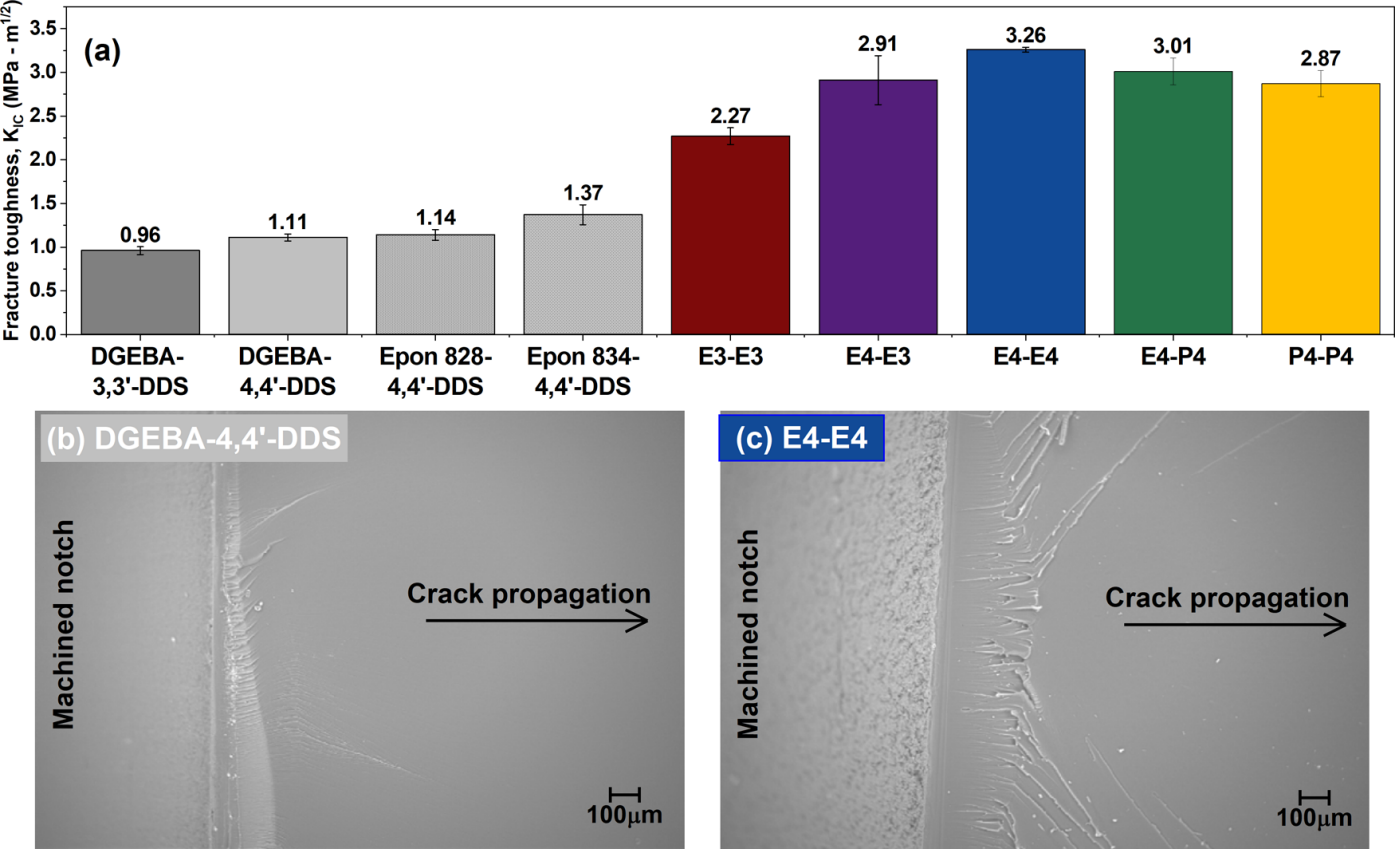
(a) Plane strain fracture toughness of symmetric polyester networks and DGEBA-DDS type networks. SEM images of the fracture surfaces of (b) a DGEBA-type network specimen and (c) a E4-E4 polyester network specimen at 50×x.

*K*_IC_ values for the symmetric polyester networks ranged from 2.0 to 3.4× those for the DGEBA monomer networks (2.27–3.26 MPa-m^1/2^ for polyester networks vs 0.96–1.11 MPa-m^1/2^ for DGEBA monomer networks) and 1.6 to 2.8x those for the oligomeric DGEBA networks (1.14 MPa-m^1/2^ for Epon 828–4,4′-DDS network and 1.37 MPa-m^1/2^ for Epon 834–4,4′-DDS network). As a baseline for comparison, a reported literature value for an unmodified Epon 828–4,4′-DDS system by Levita et al.^32^ was ca. 1.0 MPa-m^1/2^ – lower than the value obtained in this work. Yoon et al. reported an even lower *K*_IC_ value of ca. 0.7 MPa-m^1/2^ for the same unmodified system, and reported that reactive incorporation of PES increased *K*_IC_ to 2.2 MPa-m^1/2^ (ca. 3.1× that of the neat system) when loading level and PES molecular weight were optimized.^70^ The 3.1× increase in *K*_IC_ value from use of a phase-separated ductile thermoplastic in the Epon 828–4,4-DDS system is comparable to the improvement obtained from the approach used here (2.8× for E4-E4 vs Epon 828–4,4-DDS).

Within the polyester series, a statistically significant (*p* < 0.05) decrease in *K*_IC_ was observed from the fully para-substituted E4-E4 to the fully meta-substituted E3-E3 network, consistent with a less pronounced decrease from DGEBA–4,4′-DDS to DGEBA–3,3′-DDS. Though the fully meta-substituted E3-E3 network exhibited the highest tensile modulus and strength (Figure 4d), it underwent minimal postyield deformation. The more compliant E4-E4 network accommodated greater plastic strain in tension. While increased stiffness may enhance resistance to crack initiation, the ability to resist crack propagation, and thus achieve higher fracture toughness, is governed primarily by the capacity of the material for plastic deformation and energy dissipation. Accordingly, fracture behavior in these ester-linked networks is ductility-controlled rather than modulus-controlled.^71^ Although decreases in *K*_IC_ with increases in spacer length were observed among the para-substituted networks (E4-E4 > E4-P4 > P4-P4), the differences were not statistically significant. A correlation of *K*_IC_ to secondary relaxation integral value was observed among the polyester networks; systems that exhibited higher secondary mobility also showed higher fracture toughness. This suggests that local molecular motions facilitate energy dissipation. However, DGEBA-DDS-type networks displayed comparable subambient relaxation integrals with markedly different fracture behavior – so the correlation did not extend to all systems studied.

Scanning electron microscope (SEM) images of the fracture surfaces of SENB specimens indicated clear differences between representative DGEBA-type and polyester networks. A DGEBA-4,4′-DDS specimen (Figure 5b) exhibited a relatively featureless surface characteristic of brittle failure, whereas the E4-E4 specimen displayed a markedly rougher hackled topography (Figure 5c). Increased surface roughness is indicative of greater energy dissipation prior to crack propagation and is consistent with the higher fracture toughness observed for the polyester systems.

Several reference and polyester thermosets were subjected to abrupt loading (impact), as seen in Figure 6. Panels of 100 × 100 × 3.4 mm were subjected to an impact energy of 20.0 J using a 10 mm diameter radiused impactor. Load vs deflection curves (Figure 6a) were integrated to determine puncture energies (Figure 6b). All panels were fully penetrated on the impact with no apparent trends in hole area or damage pattern (Figure 6c). In contrast to the minor differences observed among DGEBA-DDS type reference networks in fracture toughness, no statistically significant differences (*p* < 0.05) were observed among the reference networks in impact resistance. The absorbed energy by the polyester panels (5489 to 7240 N-mm) was roughly 5.1 to 6.7× greater than that of the DGEBA reference networks, an indication that the higher energy dissipation ability observed under quasi-static loading extended to dynamic conditions, despite potential suppression of plastic deformation at higher strain rates. Among the polyester systems, no statistically significant differences (*p* < 0.05) were observed, preventing further assessment of the influence of substitution or spacer length on impact performance.

**Figure 6:**
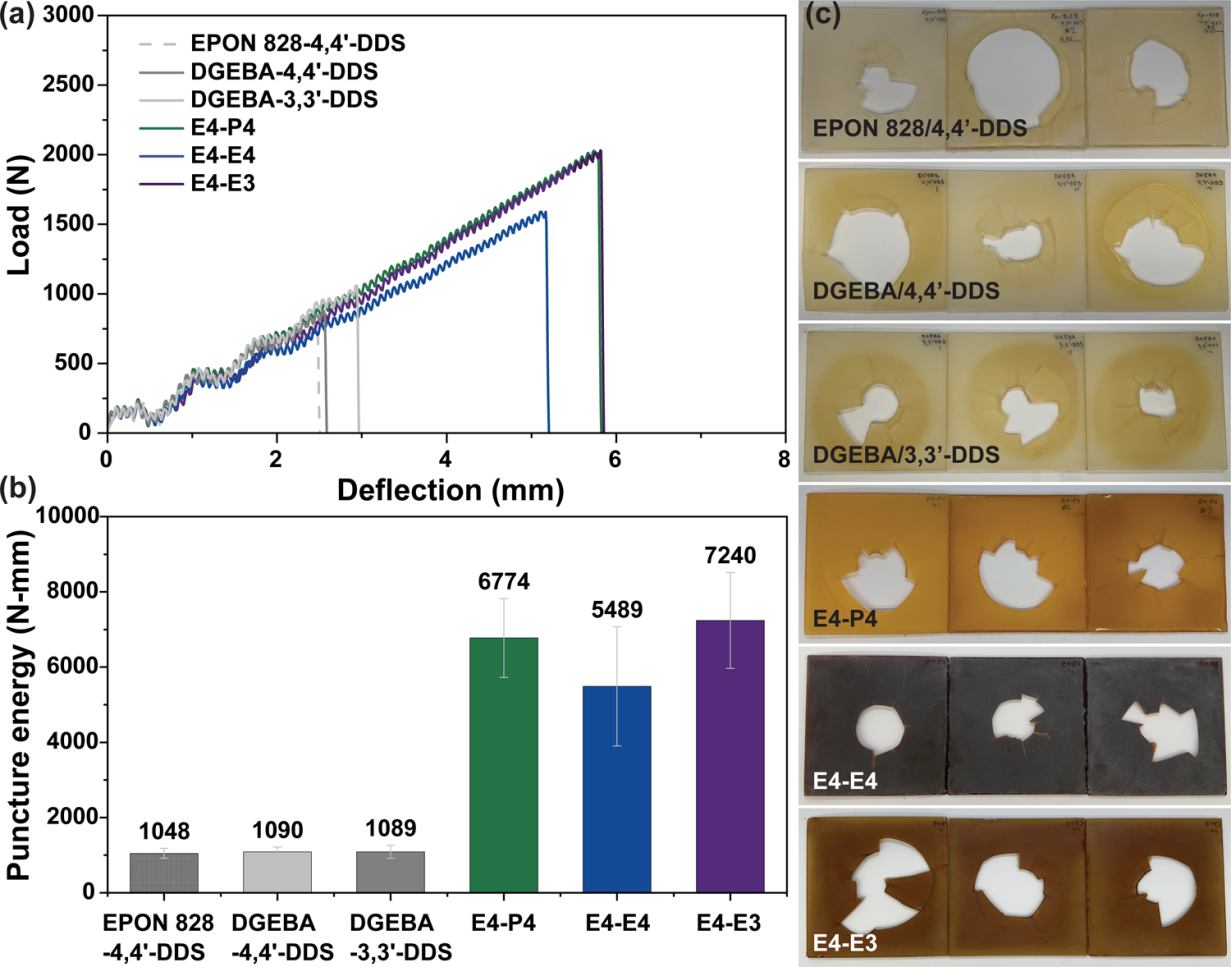
(a) Representative load–deflection curves from drop-weight impact tests, (b) averaged puncture energies for reference and polyester thermoset panels, and (c) images of test panels after impact. Welch’s *t* test (*p* < 0.05) indicated no significant differences among networks within each material type, while polyester systems absorbed significantly more energy than the reference networks.

#### 2.3.4 Hydrogen-Bonding

Differences in mechanical and thermomechanical properties among the DGEBA-type vs polyester systems could also arise from differences in secondary network structure associated with hydrogen bonding. Both polyester and DGEBA-type networks contain hydroxyl groups (from epoxide ring opening) capable of forming relatively strong hydroxyl–hydroxyl hydrogen bonds. Although the theoretical hydroxyl content is lower in the polyester networks (15% lower on average for symmetric polyester networks vs DGEBA-type networks, as seen in Table S4), ester groups can function as hydrogen bond acceptors and thereby add weaker carbonyl–hydroxyl hydrogen bonds to the secondary network structure. The presence of ester groups increases the overall number of possible interactions despite the reduction in hydroxyl functionality.

To qualitatively investigate the participation of ester groups in hydrogen bonding, mid-infrared (mid-IR) temperature sweep experiments were performed on dry E4-P4 and Epon 828 – 4,4′-DDS specimens. As seen in Figure S49, a peak associated with C=O stretch vibration shifted from about 1700 cm^–1^ at ambient temperature to 1705 cm^–1^ at 180 °C. The upshift in frequency is attributed to greater double-bond character of the carbonyl group resulting from thermally induced disruption of hydrogen bonding.^72^ Figures S50 and S51 show that the O–H stretching band shifted from ca. 3400 cm^–1^ at ambient temperature to 3560 and 3570 cm^–1^ at 180 °C for E4-P4 and Epon 828 – 4,4′-DDS, respectively – consistent with the assignment of hydrogen-bonded and free hydroxyl groups in poly­(DGEBA) by Coleman et al.^73^ Increased absorbance at ca. 3480 cm^–1^ with temperature occurs for both systems – though this is clearer for E4-P4. Absorbance in this region for polymers that contain both hydroxyl and ester functionality has been reported previously. Coleman observed a concentration-dependent increase in absorbance at ca. 3500 cm^–1^ when the polyester poly­(caprolactone) was added to poly­(DGEBA) systems.^73^ Additionally, a peak near 3480 cm^–1^ has previously attributed to hydroxyl hydrogen-bonded to furan ester carbonyl groups.^74^ The occurrence of a peak in the same area for DGEBA-4,4′DDS is possibly due to hydroxyl groups hydrogen-bound to sulfone; a peak at ca. 3490 cm^–1^ was attributed to this interaction in poly­(hydroxyethersulfone) by Lü et al.^75^

To distinguish free hydroxyl groups from those hydrogen-bonded to carbonyl, sulfone, or other hydroxyl groups, Gaussian peak fitting was applied to deconvolute the overlapping bands near 3400, 3480, and 3560 cm^–1^ in the ambient-temperature spectra. As seen in Figures S52–53, the peak at 3560 cm^–1^ – assigned to free hydroxyl groups – accounted for ∼1.5% of the total peak area in DGEBA-4,4′-DDS, whereas no discernible contribution could be fitted for E4-P4. These results indicate a higher fraction of hydroxyls engaged in hydrogen bonding within the polyester network – consistent with the high concentration of carbonyls capable of serving as hydrogen bond acceptors. The higher molecular mobility of E4-P4 and its extended window in the rubbery regime during cooling likely facilitates hydrogen bond formation by allowing hydroxyl and acceptor groups to achieve bonding distance.

#### 2.3.5 Adhesive Properties

Because substrate and filler interfaces are common in epoxy applications and interactions at those interfaces can depend on epoxy structure, we also evaluated the adhesive properties of the polyester systems by single-lap shear (Figure 7a) and peel resistance (Figure 7b). Adhesion in epoxy systems is often attributed to hydrogen bonding between hydroxyl groups in the network structure to the functionalities on polar substrates like the aluminum used for both evaluations.^76–78^ Although theoretical hydroxyl concentration in the polyester networks (Table S4) is lower than in the reference networks, all polyester networks exhibited higher failure stresses in single lap shear; this indicates that factors other than hydroxyl content contribute to their adhesive performance. The presence of polar ester groups and enhanced plastic deformability likely contribute to the improved properties observed for the polyester networks.^79^

**Figure 7:**
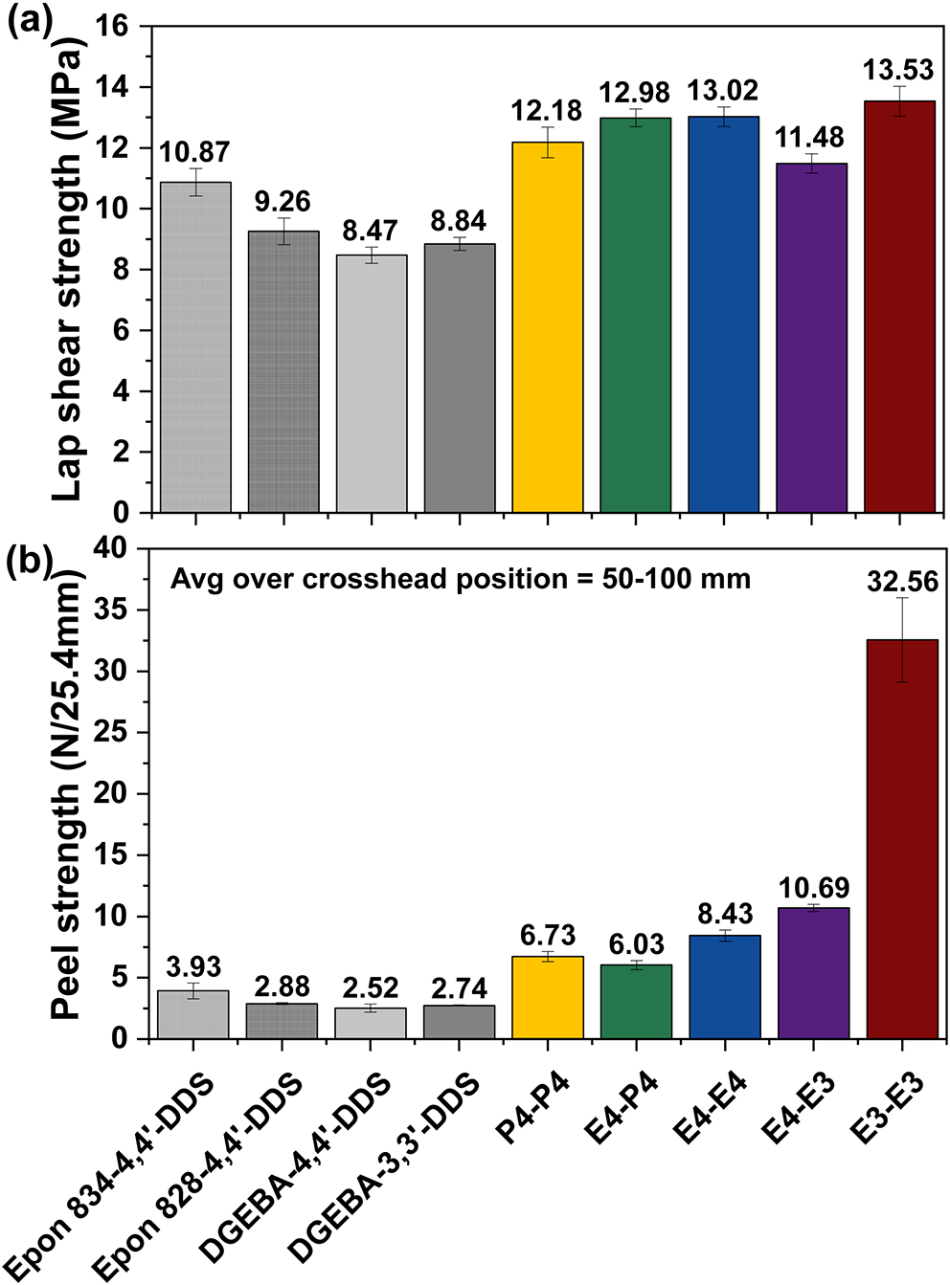
Performance of polyester and reference networks in (a) single lap shear and (b) T-peel on aluminum substates.

Increased ductility in epoxy systems has been shown to enhance peel resistance through shear yielding and plastic flow,^80^ and the introduction of flexible spacers in epoxy monomers has been reported to reduce internal stresses and by extension to increase peel resistance.^80^ Among the para-substituted polyester networks, average peel resistance showed no clear correlation to aliphatic spacer length (peel strengths of P4-P4, E4-P4, and E4-E4 were 6.73, 6.03, and 8.43 N/25.4 mm, respectively). In contrast, increased meta substitution led to a marked increase in peel strength – E4-E3 reached 10.7 N/25.4 mm, while E3-E3 reached 32.6 N/25.4 mm. This reflects the fraction of meta-substituted strands, with E4-E3 containing one-third and E3-E3 containing all meta linkages. E3-E3 and E4-E3 also exhibited the highest tensile and flexural strengths among the polyester networks. Despite similar tensile and flexural strength to those polyester networks, the DGEBA-3,3′-DDS network exhibited a peel strength of 2.74 N/25.4 mm – only marginally higher than its fully para counterpart DGEBA-4,4′-DDS (2.52 N/25.4 mm). One feature that distinguished the polyester networks from DGEBA-3,3′-DDS and DGEBA-4,4′-DDS in tension is the occurrence of yield prior to failure – an indication of higher ductility and likely contributor to their greater peel strengths.

#### 2.3.6 Water Uptake and Weathering

Epoxy–amine networks are susceptible to absorption of water due to the presence of polar functional groups (e.g., hydroxyl, ether, amine) capable of hydrogen bonding, and typically exhibit water uptake of several weight percent under high relative humidity.^81^ The absorbed water disrupts intermolecular interactions and leads to plasticization and reduction in thermomechanical and mechanical properties.^82,83^ Water uptake in these materials is governed primarily by network polarity and free volume.^84–86^ Figure 8a shows the time-dependent water uptake for Epon 828–4,4′-DDS and E4-P4 specimens upon immersion at ambient temperature. The slower sorption kinetics of the E4-P4 network likely reflect strong water–polymer interactions (e.g., hydrogen bonding between water, hydroxyl, and ester carbonyl functionalities) that impede diffusion through the network.^84,87^ Despite a higher uptake rate for Epon 828–4,4′-DDS, both systems reached similar near-equilibrium levels after 150 days (3.9% for Epon 828–4,4′-DDS and 3.7% for E4-P4).

**Figure 8:**
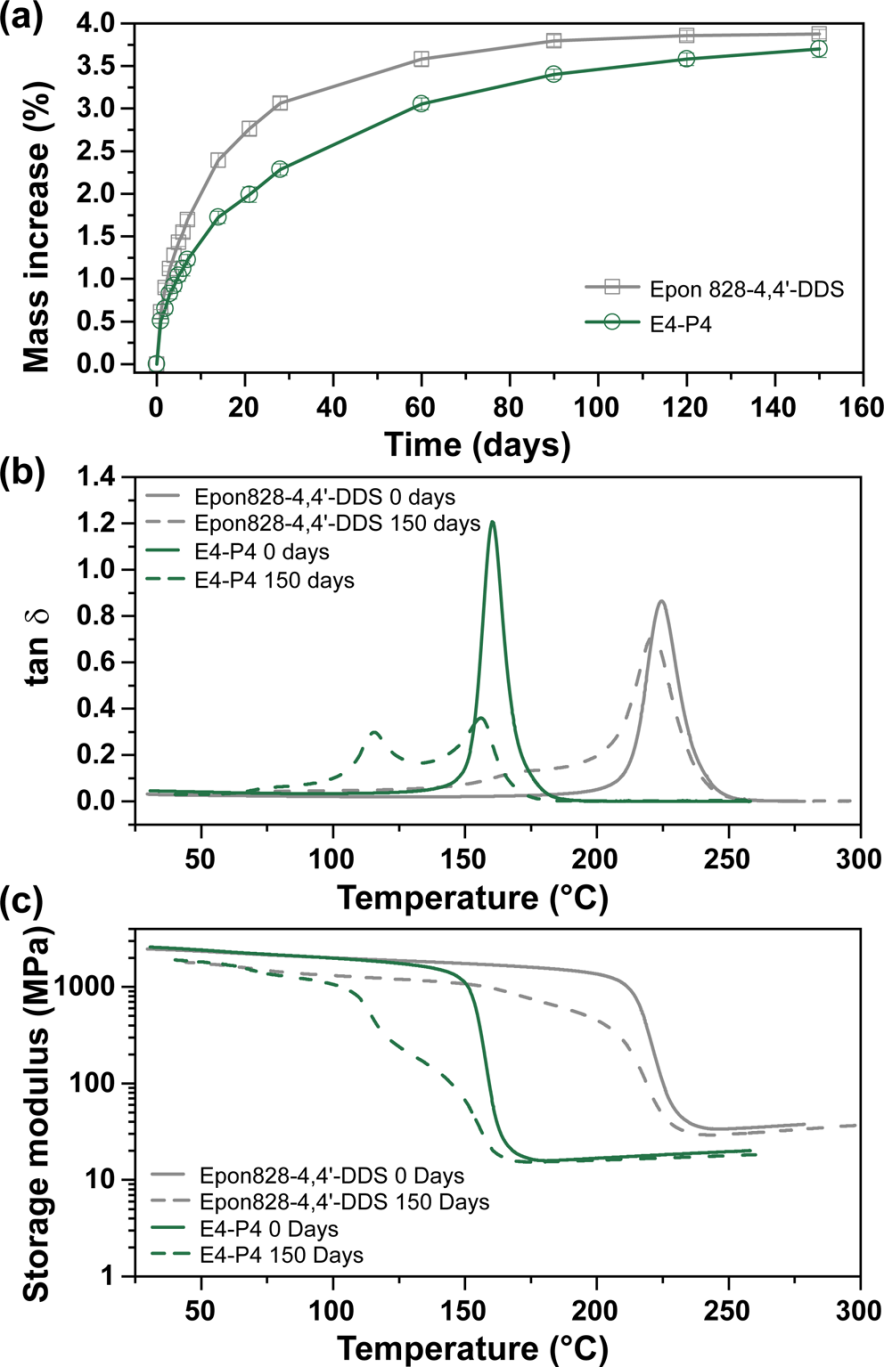
(a) Water uptake profiles for molded Epon 828–4,4′-DDS and E4-P4 samples immersed in DI water at ambient temperature. Effect of water uptake on the (b) tan δ and (c) storage moduli of Epon 828–4,4′-DDS and E4-P4 after 150 days immersion in DI water.

Despite comparable water uptake, immersion produced distinct changes in the thermomechanical behavior of the two systems (Figure 8b). Development of multimodality in tan δ and reduction of peak temperature occurred for both systems – as has been reported for aromatic epoxy-amine thermosets. The principal tan δ peak for Epon 828–4,4′-DDS decreased from 231 to 221 °C and exhibited a shoulder near 172 °C. In comparison, multimodality was severe for E4-P4; the primary tan δ peak shifted from 160 to 156 °C and a second pronounced tan δ appeared near 116 °C. Peak broadness and multimodality reflect heterogeneous plasticization within the networks arising from regions that experience different levels of water-induced plasticization.^82,83^ As water content was comparable for the two systems, a higher effect from absorbed water on E4-P4 was apparent – possibly due to greater hydrogen bond disruption. This is consistent with FTIR evidence of a more extensive hydrogen-bonding network in E4-P4 from the substantial concentration of ester carbonyl acceptor sites. Importantly, the rubbery storage modulus of E4–P4 (Figure 8c) showed only a minimal decrease after 150 days of immersion, indicating that ester hydrolysis was negligible under these conditions.

To evaluate general weatherability, specimens were exposed to 100 h of continuous full-spectrum light with intermittent water spray (18 min every 2 h) at elevated temperature, after which thermomechanical and mechanical properties were assessed. Figure 9a summarizes the resulting changes in *T*_g_ for representative DGEBA- and ester-containing networks from weathering. The DGEBA-based networks exhibited negligible changes in *T*_g_. On average, *T*_g_ for the nonester-containing systems showed only minor, statistically insignificant decreases of approximately 1%. In contrast, the polyester networks exhibited an average decrease in *T*_g_ of about 4%, with several systems showing statistically significant reductions (*p* < 0.05). Notably, the tan δ peaks for all systems remained monomodal, indicating minimal impact from water ingress and plasticization (in contrast to full water immersion). Tensile properties were also evaluated after weathering to assess mechanical durability under the same conditions. As shown in Figure 9b, the DGEBA-based networks exhibited only minor reductions in tensile strength (∼8%), remaining within experimental uncertainty. In contrast, all polyester systems showed statistically significant decreases (*p* < 0.05), with an average strength reduction of approximately 11%. The higher susceptibility of the polyester systems to weathering is consistent with the known photolytic and hydrolytic sensitivity of polyesters, which can undergo ester bond cleavage under UV and moisture exposure.^88^

**Figure 9:**
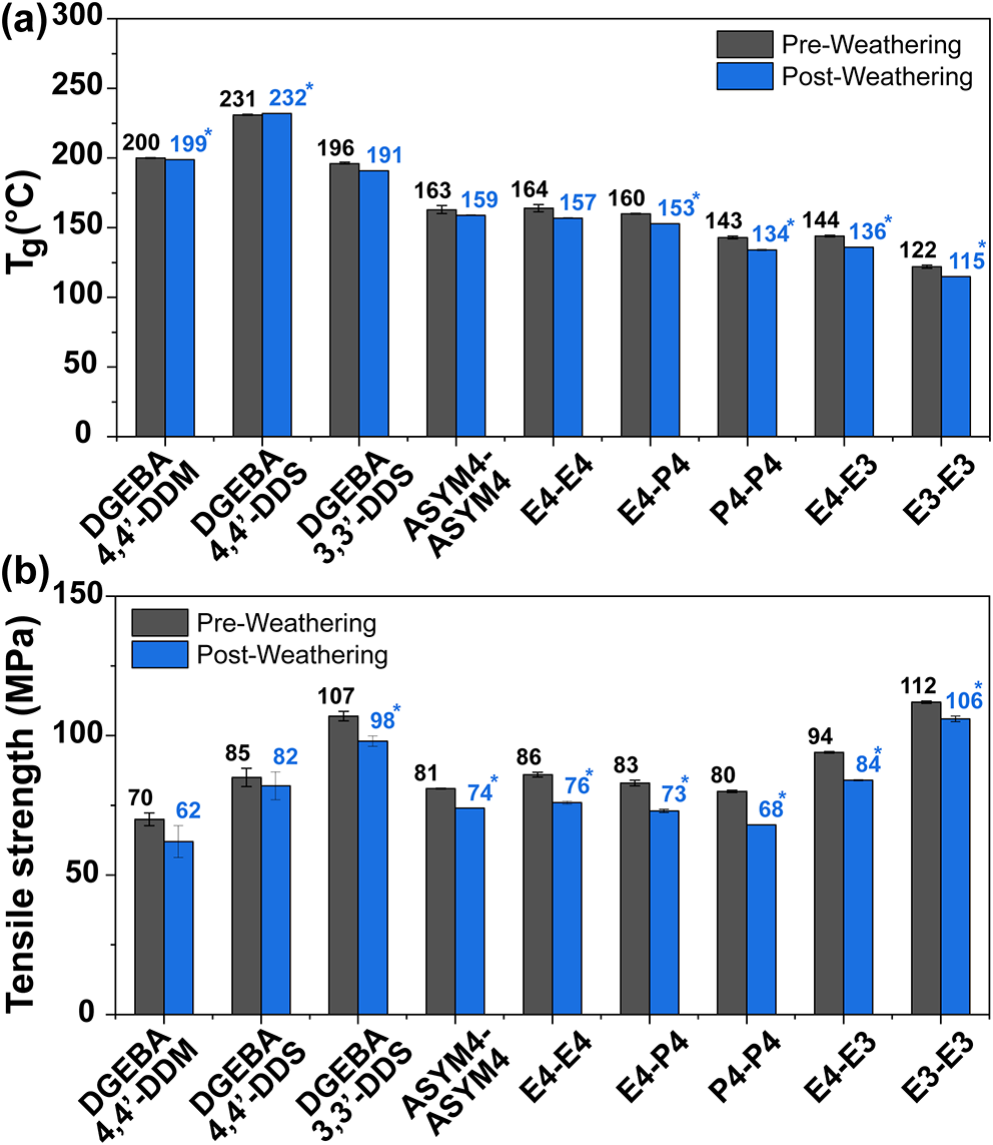
(a) Glass transition temperature and (b) tensile strength of DGEBA- and polyester-based networks before and after 100 h of exposure to ASTM G155 Cycle 1 conditions (full-spectrum light and intermittent water spray). Statistical significance was determined via Welch’s *t* test. Asterisks denote significant differences between preweathered and postweathered samples (*p* < 0.05).

### 2.4 Alcoholysis of Polyester Thermosets

The presence of ester linkages in every network strand permits thermoset deconstruction at the end-of-use. An effective deconstruction strategy for ester-linked thermosets should sever these linkages in a controlled manner, yielding soluble and chemically well-defined byproducts. For context, thermoplastic aromatic polyesters such as polyethylene terephthalate (PET) are depolymerized via alcoholysis on an industrial scale.^89,90^ A recent technoeconomic and environmental assessment by Uekert et al.^90^ demonstrated that glycolysis of PET offered superior economic and environmental metrics than methanolysis or hydrolysis. Here, we conducted ethylene glycolysis under ambient pressure at 190 °C (100:1 by mass ethylene glycol to polymer, 1 mm × 14 mm thermoset disc geometry) with 1 wt % Zn­(OAc)_2_ relative to polymer as a catalyst, as illustrated in Figure 10a. As shown in Figure 10b, PET completely degraded within 6 h, whereas the reference DGEBA–4,4′-DDS network showed no evidence of degradation over the 54 h experiment. Among the polyester thermosets, time to complete dissolution varied systematically with ester content and substitution pattern: E3-E3 < E4-E3 < E4-E4 < E4-P4 < P4-P4 < ASYM4-ASYM4. Faster degradation of the meta-substituted networks is consistent with enhanced electrophilicity of the carbonyl carbon and increased susceptibility to nucleophilic attack by ethylene glycol. The length of the aliphatic spacer also influenced degradation rate. As spacer length increased from ethyl to propyl, degradation time increased – attributable to a decrease in ester density (Table S4) due to dilution by aliphatic content. For instance, the E4-P4 network reached complete dissolution in 12 h, whereas the fully propyl-spaced P4-P4 network dissolved in 30 h under identical conditions. The ASYM4–ASYM4 network exhibited the slowest degradation (54 h), consistent with substantially lower ester content than the other polyester networks. Although the degradation products from the discs were completely soluble in ethylene glycol at 190 °C at the point of zero mass, the solutions yielded insoluble residue upon cooling to room temperature. Time-resolved GPC analysis of aliquots collected from E4-P4 glycolysis showed the presence of polymeric products after 12 h and a progressive decrease in molecular weight by 24 h (Figure 10c). Extension of glycolysis to 48 h yielded a narrow and nearly monomodal GPC trace. The material remained soluble in ethylene glycol at ambient temperature and was recovered in 67% yield as a white solid via precipitation in water, with no additional purification. Complete glycolysis of an ideally formed network would yield diols and a trihydroxyethyl ester arising from cleavage of all three ester linkages around each network junction. Figure 10d shows the ^1^H NMR spectrum of the crude degradation product, with peak assignments and integrations consistent with this predicted trihydroxyethyl ester structure. Electrospray ionization mass spectrometry (ESI-MS) further confirmed this assignment; dominant peaks at *m*/*z* 658.24 (M + H^+^), 680.22 (M + Na^+^), and 1315.48 (2 M + H^+^) were consistent with the calculated molecular mass of 657.24 Da, as seen in Figure 10e. Additional peaks at *m*/*z* 420.16 and 596.21 are potentially from a dihydroxyethyl ester with secondary amine functionality (from incomplete secondary amine conversion in the network formation process) and from cyclization via intramolecular transesterification of the described trifunctional hydroxyethyl ester (that could have occurred in the presence of the transesterification catalyst in the degradation process), respectively. It is possible that the peak at *m*/*z* 1077.40 is from two hydrogen-bonded components that share a charge: the main trihydroxyethyl ester structure (*m*/*z* 658.24) and secondary-amine dihydroxyethyl ester structure (*m*/*z* 420.16). Additional peaks at *m*/*z* 313.27 and 358.13 and the nearby region were difficult to attribute to structures possible from network formation or degradation – and are possibly related to instrumental noise.

**Figure 10:**
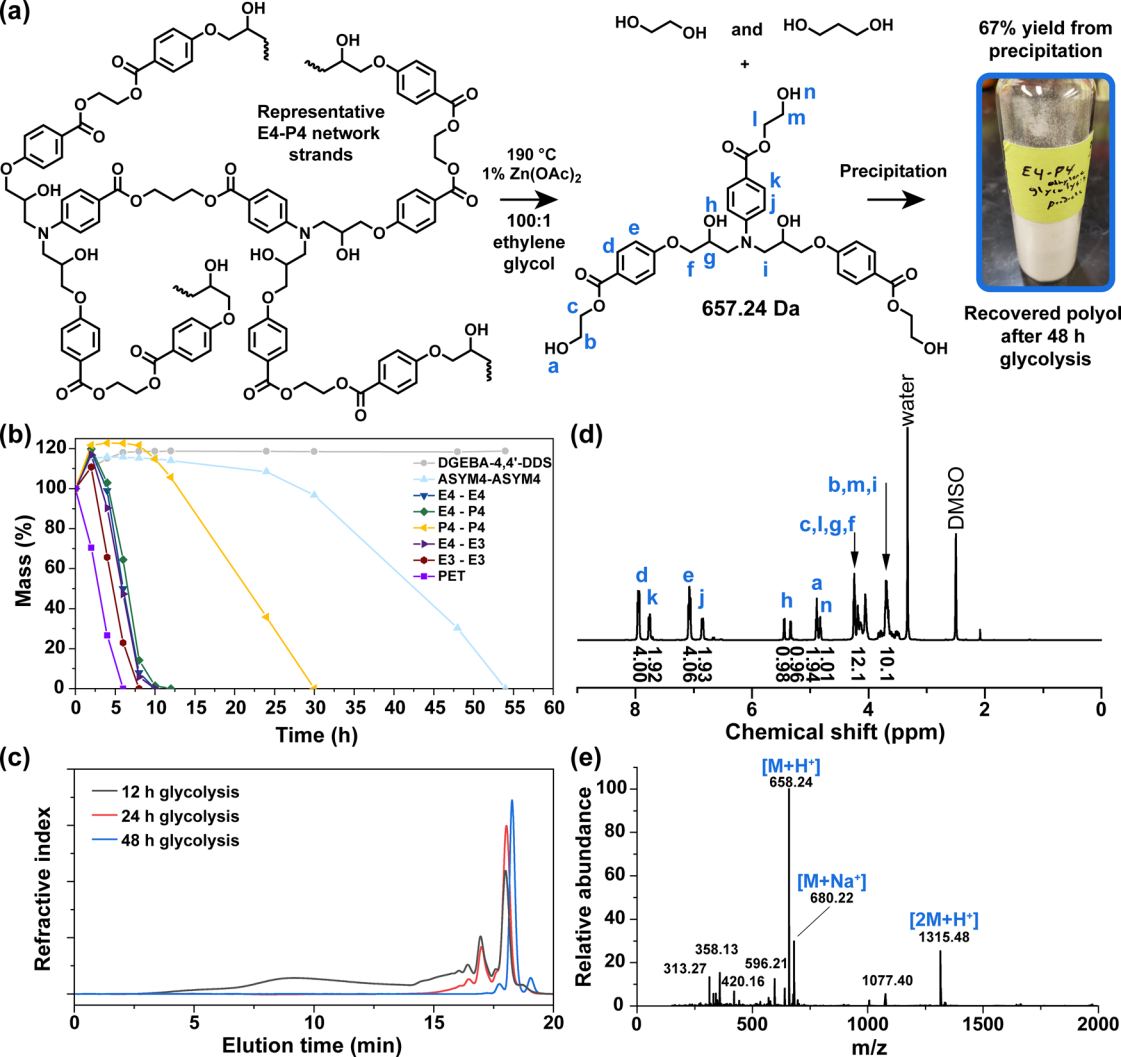
(a) Ambient-pressure glycolysis scheme for the polyester networks with E4-P4 used as an example. (b) Time-dependent mass loss curves for polyester networks, nonester reference networks, and PET under glycolysis conditions. (c) GPC chromatograms of aliquots collected at different times during glycolysis of the E4-P4 network. (d) ^1^H NMR spectrum and (e) ESI-MS of the recovered trihydroxyethyl ester degradation product.

Due to the different polymerization vs depolymerization process, the recovered product differs structurally from the epoxy and amine monomers used to prepare the network, so the process lacks circularity. Despite the lack of circularity, there is the potential to repurpose the trihydroxyethyl ester structure for polymeric materials derived from hydroxyl functional monomers, including polyurethanes or polyesters. While all polyester networks exhibited degradability, a comprehensive life cycle assessment is necessary to determine the economic and environmental practicality of implementing this strategy.

## 3 Conclusions

We have demonstrated the molecular design and synthesis of polyester thermosets from diepoxide and diamine monomers containing aromatic ester linkages and short aliphatic spacers. Variation in the structural features of the polyester networks enabled the regulation of segmental and subsegmental mobility and systematically influenced thermomechanical and mechanical properties – an indication of the modularity of this platform. Compared to DGEBA-aromatic amine systems, these thermosets exhibited lower glass transition temperatures, yet comparable strength and modulus and improved fracture toughness, impact resistance, and adhesive performance.

Materials in this platform could find utility in application areas where high cure temperatures are practical due to the subdued reactivity of the ester-containing diamines described. For example, composite applications that use a B-stage process where fracture toughness/impact resistance are particularly important. As all ester-containing monomers described are solids at ambient temperature, there is also potential utility in powder coating applications – where typical cure temperatures are in the range described in this work and where both epoxy and polyester systems are commonly employed.

The incorporation of aromatic ester linkages in every network strand enabled controlled degradation under glycolytic conditions, providing a pathway for chemical disassembly at end-of-use. Notably, glycolysis is being actively developed for industrial-scale recycling of thermoplastics such as PET, suggesting that analogous strategies for thermosets could leverage emerging chemical recycling infrastructure. This capability aligns with emerging industrial priorities aimed at reducing waste and improving the circularity of thermoset materials, where recovery and reuse remain major challenges. Collectively, these results establish a framework for designing epoxy–amine thermosets that combine performance and degradability – bridging in-service properties with responsible end-of-use management.

## 4 Experimental Methods

### 4.1 Materials

Methyl-4-hydroxybenzoate (>99%) was obtained from TCI. 3-hydroxybenzoic acid (99.5%), 4-nitrobenzoic acid (99.9%), 3-nitrobenzoic acid (99.9%), 4-nitrophenethyl alcohol (99.1%), and 4-hydroxyphenethyl alcohol (99.9%) were obtained from AmBeed. 1,2-ethanediol (≥99%) was obtained from Sigma-Aldrich or J.T. Baker, and 1,3-propanediol (≥99.6+%), ethyl-4-hydroxybenzoate (>99%), DBTDL (95%), and TBAB (≥99.0%) and Pd/C (10% Pd) were obtained from Sigma-Aldrich. ECH (99%) was obtained from Thermo-Fisher Scientific. sulfuric acid (∼97 w/w%), sodium hydroxide (≥97.0%), sodium bicarbonate, anhydrous magnesium sulfate, and solvents were obtained from Fisher Scientific. 4,4′-DDM (97%) was obtained from Sigma-Aldrich and 4,4′-DDS (Aradur 9644–1) and 3,3′-DDS were obtained from Huntsman. DGEBA was obtained from Sigma-Aldrich and Epon 828 and 834 were obtained from Hexion. Although also synthesized as a reference compound, propyl-1,3-bis-4-aminobenzoate (Polacure 470M) used for network formation was obtained from Air Products. All reagents were used without further purification unless otherwise specified.

### 4.2 Methyl-3-hydroxybenzoate

1.80 mol of 3-hydroxybenzoic acid and 18.0 mol of methanol (ca. 580 mL) were added to a 1 L one-neck round-bottom flask on an aluminum reaction block atop a stirplate. Under agitation, 0.180 mol sulfuric acid (concentrated) was added via pipet. A waterless condenser was added to the neck and the reaction was allowed to proceed for about 11 h at a block set point of 112 °C. The bulk of the methanol was removed via rotary evaporator (ca. 100 mL remained) at 60 °C and the product remained soluble. The concentrated solution was added to about 1.7 L cold DI water in a 4 L beaker and a white solid precipitated – mainly into a block at the bottom of the beaker. The block was fragmented mechanically, and sodium bicarbonate solution was added gradually under agitation to neutral pH. After an agitation period of at least 10 min, pH was rechecked and the product was vacuum-filtered and rinsed with DI water. When air-dried in a Buchner funnel, the product was pulverized using a mortar and pestle and added back to the 4 L beaker with additional DI water. The pH rechecked and the product refiltered and air-dried and then dried in a vacuum oven. Yield 80% (a white solid). ^1^H NMR (400 MHz, deuterated dimethyl sulfoxide (DMSO–*d*_6_)) δ 9.78 (s, 1H), 7.37 (d, *J* = 19.5 Hz, 2H), 7.31 (s, 1H), 7.03 (s, 1H), 3.82 (s, 3H).

### 4.3 4-hydroxyphenethyl-4-hydroxybenzoate

0.420 mol of ethyl 4-hydroxybenzoate and 0.400 mol of 4-(2-hydroxyethyl)­phenol were added to a 250 mL round-bottom flask. The temperature was increased to the point that the ethyl 4-hydroxybenzoate was molten, and 0.0044 mol of DBTDL was added under magnetic agitation. A Dean–Stark trap for collection of byproduct ethanol was connected to the flask in line with a condenser. The flask contents were further heated to about 200 °C and allowed to react for 22 h. The trap and condenser were removed in the final hours of the reaction. A nitrogen line was added the temperature was incrementally increased to 240 °C. The crude product, a solid at 240 °C, was cooled to ambient temperature, pulverized, washed two times with ethyl acetate to remove the DBTDL and residual ethyl paraben, and used without further purification. Yield = 78% (a white solid). ^1^H NMR (400 MHz, DMSO–*d*_6_) δ 10.26 (s, 2H), 9.21 (s, 2H), 7.81–7.74 (m, 4H), 7.11–7.05 (m, 4H), 6.88–6.80 (m, 4H), 6.73–6.65 (m, 4H), 4.33 (t, *J* = 6.8 Hz, 4H), 2.87 (t, *J* = 6.9 Hz, 4H). ^13^C NMR (101 MHz, DMSO–*d*_6_) δ 165.72 (C), 162.16 (C), 156.06 (C), 131.56 (CH), 129.87 (CH), 128.32 (C), 120.69 (C), 115.53 (CH), 115.39 (CH), 65.25 (CH_2_), 33.95 (CH_2_).

### 4.4 Ethyl-1,2-bis-4-hydroxybenzoate

0.260 mol of methyl p-hydroxybenzoate and 0.520 mol ethylene glycol were added to a 250 mL 1-neck round-bottom flask. The temperature was increased to the point that the methyl 4-hydroxybenzoate was molten, and 0.0029 mol of DBTDL was added under magnetic agitation. The flask was fitted with a Dean–Stark trap and a condenser to collect byproduct methanol. The flask was insulated, and the temperature was increased to 170–180 °C. When the transesterification reaction proceeded for 10 h, the temperature was lowered to 100 °C and the trap and condenser were replaced with a short path vacuum distillation apparatus and receiving flask. Vacuum was applied via a 0.9 CFM rotary vane pump and the temperature was gradually increased to 180 °C to control the distillation of free ethylene glycol. The ester interchange reaction proceeded at 180 °C for 8 h. At ambient temperature, the solid crude product was thoroughly pulverized with a mortar and pestle and washed 2x in a beaker under agitation with diethyl ether to remove the transesterification catalyst. Yield = 84% (white solid). 1H NMR (400 MHz, DMSO–*d*_6_) δ 10.32 (s, 2H), 7.85–7.76 (m, 4H), 6.88–6.79 (m, 4H), 4.53 (s, 4H). 13C NMR (101 MHz, DMSO–*d*_6_) 165.47 (C), 162.11 (C), 131.51 (CH), 120.12 (C), 115.38 (CH), 62.33 (CH_2_).

### 4.5 Ethyl-1,2-bis-3-hydroxybenzoate

0.260 mol of methyl m-hydroxybenzoate and 0.520 mol ethylene glycol were added to a 250 mL 1-neck round-bottom flask. The temperature was increased to the point that the methyl m-hydroxybenzoate was molten, and 0.0029 mol of DBTDL was added under magnetic agitation. The flask was fitted with a Dean–Stark trap and a condenser to collect byproduct methanol. The flask was insulated, and the temperature was increased to 170–180 °C. When the transesterification reaction proceeded for 11 h, the temperature was lowered to 100 °C and the trap and condenser were replaced with a short path vacuum distillation apparatus and receiving flask. Vacuum was applied via a 0.9 CFM rotary vane pump and the temperature was gradually increased to 180 °C to control the distillation of free ethylene glycol. The ester interchange reaction proceeded at 180 °C for 8 h. At ambient temperature, the solid crude product was thoroughly pulverized with a mortar and pestle and washed 2x in a beaker under agitation with dichloromethane to remove the transesterification catalyst. Yield = 87% (white solid). ^1^H NMR (400 MHz, DMSO–*d*_6_) δ 9.83 (s, 1H), 7.45–7.34 (m, 2H), 7.31 (t, *J* = 7.8 Hz, 1H), 7.03 (ddd, *J* = 8.1, 2.6, 1.1 Hz, 1H), 4.59 (s, 2H). 13C NMR (101 MHz, DMSO–*d*_6_) 165.65 (C), 157.52 (C), 130.69 (C), 129.85 (CH), 120.48 (CH), 119.88 (CH), 115.66 (CH), 62.74 (CH_2_).

### 4.6 Propyl-1,3-bis-4-hydroxybenzoate

0.260 mol of methyl p-hydroxybenzoate and 0.130 mol 1,3-propanediol were added to a 250 mL 1-neck round-bottom flask. The temperature was increased to the point that the methyl p-hydroxybenzoate was molten, and 0.058 mol of DBTDL were added under magnetic agitation. The flask was fitted with a Dean–Stark trap and a condenser to collect byproduct methanol, and the flask was insulated, and the temperature was increased to 170–180 °C. When the transesterification reaction proceeded for 10 h, the trap and condenser were removed and nitrogen flow was introduced to the headspace to promote the ester interchange reaction. At ambient temperature, the solid crude product was thoroughly pulverized with a mortar and pestle and washed 2x in a beaker under agitation with diethyl ether to remove the transesterification catalyst. Yield = 78% (white solid). ^1^H NMR (400 MHz, DMSO–*d*_6_) δ 10.29 (s, 2H), 7.86–7.77 (m, 4H), 6.88–6.79 (m, 4H), 4.35 (t, *J* = 6.2 Hz, 4H), 2.13 (h, *J* = 6.6 Hz, 2H). ^13^C NMR (101 MHz, DMSO–*d*_6_) δ 165.54 (C), 161.97 (C), 131.43 (CH), 120.35 (C), 115.28 (CH), 61.29 (CH_2_), 27.90 (CH_2_).

### 4.7 Diepoxide Monomer Syntheses

0.100 mol of bisphenol component, 0.010 mol of TBAB, and 2.000 mol of ECH were added to a 500 mL 1-neck round-bottom flask. For certain diepoxides, a lower reaction scale was used (see below). The flask contents were agitated magnetically and heated to about 100 °C. The ECH completely solvated the bisphenols in all cases at this temperature. The bisphenol-ECH coupling reaction was allowed to proceed for about 1 h, then the flask temperature was lowered to about 27 °C via water bath application to the underside of the flask. 0.400 mol of sodium hydroxide in a 5 M solution in water with 0.010 mol additional TBAB were added via an addition funnel to the flask, and the temperature was maintained in the range 25–30 °C for about 30 min. The flask contents were transferred to a 1L separatory funnel; 350 mL of ethyl acetate (or dichloromethane where noted below) and 150 mL of DI water were used to rinse the flask contents into the separatory funnel. The organic phase was subjected to three additional DI water washes. The organic phase was dried using MgSO_4_ and concentrated via rotary evaporation and then via Schlenk line briefly. Each monomer was then redissolved in dichloromethane, passed through a silica plug to remove TBAB, reconcentrated, and used without further purification.

#### 4.7.1 4-Glycidyloxyoxyphenethyl-4-glycidyloxybenzoate

White solid (76% yield). mp. 102 °C. *m*/*z* (Na^+^) = 393.13113 (vs 393.130860 expected). EEW = 184 g/eq (flash chromatography), 192 g/eq (silica plug only). ^1^H NMR (400 MHz, CDCl_3_) δ 8.00–7.92 (m, 2H), 7.23–7.14 (m, 2H), 6.97–6.89 (m, 2H), 6.93–6.83 (m, 2H), 4.46 (t, *J* = 7.0 Hz, 2H), 4.29 (dd, *J* = 11.1, 3.0 Hz, 1H), 4.19 (dd, *J* = 11.0, 3.2 Hz, 1H), 3.97 (ddd, *J* = 16.5, 11.1, 5.7 Hz, 2H), 3.40–3.30 (m, 2H), 3.00 (t, *J* = 7.0 Hz, 2H), 2.90 (dt, *J* = 10.1, 4.5 Hz, 2H), 2.75 (ddd, *J* = 7.6, 4.9, 2.6 Hz, 2H). ^13^C NMR (101 MHz, CDCl_3_) 166.19 (C), 162.25 (C), 157.35 (C), 131.68 (CH), 130.76 (C), 130.05 (CH), 123.38 (C), 114.84 (CH), 114.31 (CH), 68.93 (CH_2_), 65.46 (CH_2_), 50.24 (CH), 49.97 (CH), 44.77 (CH_2_), 44.65 (CH_2_), 34.49 (CH_2_).

#### 4.7.2 Ethyl-1,2-bis-4-glycidyloxybenzoate

White solid (80% yield). mp. 82 °C. *m*/*z* (Na^+^) = 437.12115 (vs 437.120689 expected). EEW = 205 g/eq (flash chromatography), 213 g/eq (silica plug only). ^1^H NMR (400 MHz, CDCl_3_) δ 8.04–7.95 (m, 4H), 6.97–6.88 (m, 4H), 4.61 (s, 4H), 4.28 (dd, *J* = 11.1, 3.0 Hz, 2H), 3.98 (dd, *J* = 11.1, 5.7 Hz, 2H), 3.36 (ddd, *J* = 5.8, 4.0, 2.8 Hz, 2H), 2.91 (t, *J* = 4.5 Hz, 2H), 2.75 (dd, *J* = 4.9, 2.6 Hz, 2H). ^13^C NMR (101 MHz, CDCl_3_) δ 166.05 (C), 162.43 (C), 131.87 (CH), 122.90 (C), 114.37 (CH), 68.94 (CH_2_), 62.66 (CH_2_), 49.96 (CH), 44.64 (CH_2_).

#### 4.7.3 Propyl-1,3-bis-4-glycidyloxybenzoate

White solid (75% yield -0.075 mol scale). Dichloromethane was used for extraction as opposed to ethyl acetate. mp. 110 °C. *m*/*z* (Na^+^) = 451.13696 (vs 451.136339 expected). EEW = 217 g/eq (flash chromatography), 218 g/eq (silica plug only). ^1^H NMR (400 MHz, CDCl_3_) δ 8.01–7.93 (m, 4H), 6.95–6.87 (m, 4H), 4.46 (t, *J* = 6.2 Hz, 4H), 4.28 (dd, *J* = 11.0, 3.0 Hz, 2H), 3.98 (dd, *J* = 11.1, 5.8 Hz, 2H), 3.36 (ddd, *J* = 5.7, 4.0, 2.8 Hz, 2H), 2.91 (t, *J* = 4.5 Hz, 2H), 2.76 (dd, *J* = 4.9, 2.6 Hz, 2H), 2.22 (p, *J* = 6.2 Hz, 2H). ^13^C NMR (101 MHz, CDCl_3_) δ 166.19 (C), 162.30 (C), 131.73 (CH), 123.15 (C), 114.30 (CH), 68.94 (CH_2_), 61.68 (CH_2_), 49.95 (CH), 44.63 (CH_2_), 28.42 (CH_2_).

#### 4.7.4 Ethyl-1,2-bis-3-glycidyloxybenzoate

White solid (82% yield). mp. 85 °C. *m*/*z* (Na^+^) = 437.12015 (vs 437.120689 expected). EEW = 209 g/eq (flash chromatography), 214 g/eq (silica plug only). ^1^H NMR (400 MHz, CDCl_3_) δ 7.67 (s, 2H), 7.57 (s, 2H), 7.34 (s, 2H), 7.13 (s, 2H), 4.65 (s, 4H), 4.25 (s, 2H), 3.95 (s, 2H), 3.34 (s, 2H), 2.89 (s, 2H), 2.74 (s, 2H). ^13^C NMR (101 MHz, CDCl_3_) δ 166.13 (C), 158.56 (C), 131.25 (C), 129.65 (CH), 122.71 (CH), 120.38 (CH), 114.98 (CH), 69.09 (CH_2_), 62.86 (CH_2_), 50.04 (CH), 44.61 (CH_2_).

#### 4.7.5 Ethyl-glycidyloxybenzoate (Monoepoxide to Check for Amidation Reaction)

White solid *(*85% yield - 0.200 mol scale) synthesized via the above-described procedure. Purified via flash chromatography (1:2 ethyl acetate:hexanes) to remove minor level of glycidyl ester. ^1^H NMR (400 MHz, DMSO–*d*_6_) δ 7.92 (s, 2H), 7.07 (s, 2H), 4.44 (s, 1H), 4.29 (s, 2H), 3.92 (s, 1H), 3.36 (s, 1H), 2.86 (s, 1H), 2.74 (s, 1H), 1.31 (s, 3H).

### 4.8 Methyl 3-nitrobenzoate

0.540 mol of 3-nitrobenzoic acid and 5.40 mol of methanol (ca. 170 mL) were added to a 500 mL one-neck round-bottom flask. Under agitation, 0.054 mol of sulfuric acid (concentrated) was added via pipet. A waterless condenser was added to the neck and the reaction was allowed to proceed for about 12 h at a block set point of 110 °C. Liquid–liquid phase-separation occurred on cooldown and then a white solid formed. The methanol was removed via vacuum filtration. The filtrand was fragmented mechanically, rinsed with DI water, and added to a 4 L beaker. A solution of sodium bicarbonate was added gradually under agitation to neutral pH. After a period of agitation and several pH rechecks, the product was vacuum- filtered. After air-drying in a Buchner funnel, the product was pulverized using a mortar and pestle and added back to the 4 L beaker with additional DI water. After rechecking the pH, the product was filtered, air-dried, and then dried in a vacuum oven. Yield 89% (a white solid). ^1^H NMR (400 MHz, CDCl_3_) δ 8.85 (s, 1H), 8.39 (d, *J* = 19.8 Hz, 2H), 7.65 (s, 1H), 3.98 (s, 3H).

### 4.9 Methyl 4-Nitrobenzoate

0.540 mol of 4-nitrobenzoic acid and 5.40 mol of methanol (ca. 170 mL) were added to a 500 mL one-neck round-bottom flask. Under agitation, 0.054 mol of sulfuric acid (concentrated) was added via pipet. A waterless condenser was added to the neck and the block set point was moved to 110 °C. Initially, dissolution of 4-nitrobenzoic acid was incomplete; additional methanol (about 90 mL) was added. The reaction was allowed to proceed for about 11.5 h at a block set point of 110 °C – where complete dissolution occurred over the course of the reaction. On cooldown, a white precipitate formed. About 600 mL of ethyl acetate were added and the product was rinsed in a separatory funnel with sodium bicarbonate solution and then DI water. The ethyl acetate phase was concentrated via rotary evaporator and residual solvent was removed under vacuum. Yield 90% (a white solid). ^1^H NMR (400 MHz, DMSO–*d*_6_) δ 8.34 (s, 1H), 8.16 (s, 1H), 3.91 (s, 2H).

### 4.10 4-Nitrophenethyl-4-nitrobenzoate

0.210 mol of methyl 4-nitrobenzoate and 0.200 mol of 4-nitrophenethyl alcohol were added to a 250 mL 1-neck round-bottom flask. The temperature was increased to the point that the methyl 4-nitrobenzoate was molten, and 0.0022 mol of DBTDL was added under magnetic agitation. A Dean–Stark trap for collection of byproduct methanol was connected to the flask in line with a condenser and the flask contents were further heated to about 200 °C and allowed to react for 10 h. The trap and condenser were removed in the final hours of the reaction and replaced with a nitrogen line through the open port. The crude product was cooled to ambient temperature, pulverized, washed two times with diethyl ether to remove the DBTDL and residual methyl 4-nitrobenzoate, and used without further purification. Yield = 85% (a beige solid). ^1^H NMR (400 MHz, DMSO–*d*_6_) δ 8.31 (s, 2H), 8.18 (s, 2H), 8.12 (s, 2H), 7.64 (s, 2H), 4.61 (s, 2H), 3.23 (s, 2H). ^13^C NMR (101 MHz, DMSO–*d*_6_) δ 164.02 (C), 150.20 (C), 146.37 (C), 146.30 (C), 134.86 (C), 130.49 (CH), 130.20 (CH), 123.81 (CH), 123.40 (CH), 65.21 (CH_2_), 33.94 (CH_2_).

### 4.11 Ethyl-1,2-bis-4-nitrobenzoate

0.700 mol of methyl 4-nitrobenzoate and 1.400 mol of ethylene glycol were added to a 1L 1-neck round-bottom flask. The temperature was increased to the point that the methyl 4-hydroxybenzoate was molten, and 0.0077 mol of DBTDL was added under magnetic agitation. A Dean–Stark trap for collection of byproduct methanol was connected to the flask in line with a condenser and the flask contents were further heated to about 180 °C and allowed to react for 3 h. The trap and condenser were then removed and replaced with a nitrogen line through the open port. The reaction continued at 180 °C for 5 h. At this point, the temperature was lowered to 100 °C and a short path vacuum distillation apparatus and receiving flask were added. Vacuum was applied via a 0.9 CFM rotary vane pump and the temperature was gradually increased to 200 °C to control the distillation of free ethylene glycol. The ester interchange reaction proceeded at 200 °C for 11 h. The crude product was cooled to ambient temperature, pulverized, washed two times with diethyl ether to remove the DBTDL and residual methyl 4-nitrobenzoate, and used without further purification. Yield = 84% (a beige solid). ^1^H NMR (400 MHz, DMSO–*d*_6_) δ 8.32 (s, 4H), 8.19 (s, 4H), 4.71 (s, 4H). ^13^C NMR (101 MHz, DMSO–*d*_6_) δ 164.17 (C), 150.26 (C), 134.78 (C), 130.63 (CH), 123.83 (CH), 63.44 (CH_2_).

### 4.12 Propyl-1,3-bis-4-nitrobenzoate

0.120 mol of methyl 4-nitrobenzoate and 0.240 mol of 1,3-propanediol were added to a 100 mL 1-neck round-bottom flask. The temperature was increased to the point that the methyl 4-nitrobenzoate was molten, and 0.0011 mol of DBTDL was added under magnetic agitation. A Dean–Stark trap for collection of byproduct methanol was connected to the flask in line with a condenser and the flask contents were further heated to about 180 °C and allowed to react for 2 h. The trap and condenser were then removed and replaced with a nitrogen line through the open port. The reaction continued at 180 °C for 4 h. At this point, the temperature was lowered to 100 °C and a short path vacuum distillation apparatus and receiving flask were added. Vacuum was applied via a 0.9 CFM rotary vane pump and the temperature was gradually increased to 200 °C. The ester interchange reaction proceeded at 200 °C for 2 h and 210, 220, 230, and 240 °C for about 1 h at each temperature. The crude product was cooled to ambient temperature, pulverized, washed two times with diethyl ether to remove the DBTDL and residual methyl 4-nitrobenzoate. Yield = 80% (beige-orange solid). ^1^H NMR (400 MHz, DMSO–*d*_6_) δ 8.26 (s, 4H), 8.15 (s, 4H), 4.51 (s, 4H), 2.25 (s, 2H). ^13^C NMR (101 MHz, DMSO–*d*_6_) δ 165.85 (C), 153.48 (C), 131.09 (CH), 115.85 (C), 112.65 (CH), 60.63 (CH_2_), 28.08 (CH_2_).

### 4.13 Ethyl-1,2-bis-3-nitrobenzoate

1.200 mol of methyl 3-nitrobenzoate and 2.400 mol of ethylene glycol were added to a 1L 1-neck round-bottom flask. The temperature was increased to the point that the methyl 3-nitrobenzoate was molten, and 0.0132 mol of DBTDL was added under magnetic agitation. A Dean–Stark trap for collection of byproduct methanol was connected to the flask in line with a condenser and the flask contents were further heated to about 180 °C and allowed to react for 3 h. The trap and condenser were then removed and replaced with a nitrogen line through the open port. The reaction continued at 180 °C for 5 h. At this point, the temperature was lowered to 100 °C and a short path vacuum distillation apparatus and receiving flask were added. Vacuum was applied via a 0.9 CFM rotary vane pump and the temperature was gradually increased to 200 °C to control the distillation of free ethylene glycol. The ester interchange reaction proceeded at 200 °C for 11 h, 210 °C for 1 h, and 220 °C for 1 h. The crude product was cooled to ambient temperature, cryofractured, pulverized, and washed two times with diethyl ether to remove the DBTDL and residual methyl 3-nitrobenzoate. The solid product was dissolved in dichloromethane and passed through a silica plug to remove residual monocondensation adduct. Yield = 81% (beige solid). ^1^H NMR (400 MHz, DMSO–*d*_6_) δ 8.62 (s, 2H), 8.48 (s, 2H), 8.35 (s, 2H), 7.83 (s, 2H), 4.74 (s, 4H). ^13^C NMR (101 MHz, DMSO–*d*_6_) δ 163.94 (C), 147.88 (C), 135.22 (CH), 130.97 (C), 130.72 (CH), 127.85 (CH), 123.60 (CH), 63.42 (CH_2_).

### 4.14 Diamine Syntheses

A sealed 2-neck 1L round-bottom flask with a magnetic stir bar was purged via a vacuum pump and backfilled with dry nitrogen via Schlenk line techniques. 0.00120 mol Pd (as 10% in activated carbon) was added to the flask. The flask was opened briefly and 0.050 mol of dinitro precursor predissolved in tetrahydrofuran was added (a typical concentration of dinitro compound in tetrahydrofuran was 0.1 mol/L). Under agitation, the resealed flask was again purged and filled with nitrogen (4× cycles). A balloon within a balloon was filled with nitrogen, nearly emptied, then filled with hydrogen. The balloon was connected to a valve equipped with a Luer-Lok on the opposite end. A needle was connected to the Luer-Lok end and was inserted into one of the rubber septa on the flask under static vacuum. The reaction proceeded initially at ambient temperature, and the temperature was gradually ramped to ca. 30 °C over a ca. 30-h reaction period. Additional hydrogen was supplied (balloon replaced) upon depletion.

#### 4.14.1 4-Aminophenethyl-4-aminobenzoate

Beige solid (95% yield at 0.0500 mol scale). mp. 133 °C. *m*/*z* (Na^+^) = 279.11017 (vs 279.110399 expected). ^1^H NMR (400 MHz, DMSO–*d*_6_) δ 7.65–7.58 (m, 2H), 6.96–6.90 (m, 2H), 6.60–6.53 (m, 2H), 6.53–6.47 (m, 2H), 5.92 (s, 2H), 4.85 (s, 2H), 4.24 (t, *J* = 7.0 Hz, 2H), 2.78 (t, *J* = 7.0 Hz, 2H). ^13^C NMR (101 MHz, DMSO–*d*_6_) δ 165.82 (C), 153.40 (C), 146.92 (C), 130.99 (CH), 129.25 (CH), 124.92 (C), 116.01 (C), 113.98 (CH), 112.62 (CH), 64.74 (CH_2_), 33.90 (CH_2_).

#### 4.14.2 Ethyl-1,2-bis-4-aminobenzoate

Beige solid (97% yield at 0.0500 mol scale). mp. 214 °C. *m*/*z* (Na^+^) = 323.10001 (vs 323.100228 expected). ^1^H NMR (400 MHz, DMSO–*d*_6_) δ 7.64 (s, 4H), 6.55 (s, 4H), 5.95 (s, 4H), 4.44 (s, 4H). ^13^C NMR (101 MHz, DMSO–*d*_6_) δ 165.73 (C), 153.58 (C), 131.14 (CH), 115.54 (C), 112.63 (CH), 61.89 (CH_2_).

#### 4.14.3 Propyl-1,3-bis-4-aminobenzoate

Synthesized as a reference only (Air Products Polacure 470 M was used for network synthesis). beige solid (92% yield at 0.0160 mol scale). mp. = 124 °C. *m*/*z* (Na^+^) = 337.11575 (vs 337.115878 expected). ^1^H NMR (400 MHz, DMSO–*d*_6_) δ 7.65 (s, 4H), 6.57 (s, 4H), 5.93 (s, 4H), 4.28 (s, 4H), 2.07 (s, 2H). ^13^C NMR (101 MHz, DMSO–*d*_6_) δ 165.73 (C), 153.58 (C), 131.14 (CH), 115.54 (C), 112.63 (CH), 61.89 (CH_2_), 30.62 (CH_2_).

#### 4.14.4 Ethyl-1,2-bis-3-aminobenzoate

Beige solid (95% yield at 0.0400 mol scale). mp. 137 °C. *m*/*z* (Na^+^) = 323.10004 (vs 323.100228 expected). ^1^H NMR (400 MHz, DMSO–*d*_6_) δ 8.62 (s, 2H), 8.48 (s, 2H), 8.35 (s, 2H), 7.83 (s, 2H), 4.74 (s, 4H). ^13^C NMR (101 MHz, DMSO–*d*_6_) δ 163.94 (C), 147.88 (C), 135.22 (CH), 130.97 (C), 130.72 (CH), 127.85 (CH), 123.60 (CH), 63.42 (CH_2_).

#### 4.14.5 Ethyl-aminobenzoate (Monoamine to Check for Amidation Reaction)

White solid *(*81% yield -0.120 mol scale) synthesized using Fischer esterification process. Briefly, 0.120 mol of *p*-aminobenzoic acid and 4.8 mol of ethanol (280 mL) were added to a 500 mL round-bottom flask. Under agitation, initially 0.132 mol of sulfuric acid was added. Dissolution of the amine salt in ethanol was incomplete, so an additional 0.084 mol of sulfuric acid was added and complete dissolution was achieved at ethanol reflux. The reaction was allowed to proceed for about 4 h at reflux. The flask contents were cooled and transferred to a 4L beaker. At subambient temperature, saturated sodium bicarbonate (aq) solution was added gradually until about pH 8, and the white precipitate that formed was vacuum-filtered. The filtrand was washed with DI water, dried under vacuum, and used without further purification. ^1^H NMR (400 MHz, DMSO–*d*_6_) δ 7.63 (s, 2H), 6.56 (s, 2H), 5.91 (s, 2H), 4.20 (s, 2H), 1.26 (s, 3H).

### 4.15 Network Synthesis

Diepoxide (mass dependent on the type of part formed) was weighed into a tared container with a polytetrafluoroethylene (PTFE)-coated stir bar and the tare weight was recorded. The container was transferred to a vacuum oven and the monomer was degassed at ∼120 °C for a period of about 1 h. In a separate vacuum oven, a vial filled roughly halfway with diamine was degassed at ∼100 °C. The container of degassed epoxy monomer was reweighed and the tare weight subtracted. The container was agitated on a magnetic stir plate at 100–130 °C (system-dependent). The vial of degassed diamine was removed and the weight of diamine to add to achieve a 1:1 epoxide functional group to amine hydrogen ratio was determined from the weight of the degassed diepoxide. This weight of diamine was added to an empty vial. The filled vial was tared and added to the epoxy monomer under agitation; the vial was reweighed to confirm the added weight. Agitation of the vial contents at 100 °C (DGEBA-4,4′-DDM, ASYM4-ASYM4) or 120 °C (DGEBA-4,4′-DDS, DGEBA-3,3′-DDS, E4-P4, P4-P4, E4-E3, E3-E3) or 160 °C (E4-E4) continued until an optically clear solution formed (∼10 min). The vial contents were then degassed at the agitation temperature (or at 130 °C for E4-E4) for about 10 min and transferred via pipet to a preheated high temperature (HT) silicone mold in a convection oven programmed to run the following profile: 3 h at 100 °C, ramp 100 to 200 °C over 2 h, 200 °C for 2 h (DGEBA-4,4′-DDM) or 3 h at 110 °C, ramp 110 to 200 °C over 2 h, 200 °C for 2 h (ASYM4-ASYM4) or 3 h at 120 °C, ramp 120 to 200 °C over 2 h, 200 °C for 2 h (DGEBA-4,4′-DDS, DGEBA-3,3′-DDS, E4-P4, P4-P4, E4-E3, and E3-E3) or 3 h at 130 °C, ramp 130 to 200 °C over 2 h, 200 °C for 2 h (E4-E4).

### 4.16 NMR Spectroscopy

^1^H and ^13^C spectra of the synthesized bisphenols, diarylnitro compounds, diepoxides, and diamines were obtained using a Bruker Avance NEO 400 MHz spectrometer. DMSO–*d*_6_ and CDCl_3_ were used as lock solvents. For all ^1^H analysis, the nominal analyte concentration was 40 mg/mL, and 32 scans were taken using a recycle/relaxation delay (d1) of 5 s. For all ^13^C NMR analysis, the nominal analyte concentration was 80 mg/mL, and 256 scans were taken using a d1 time of 2 s. Data were analyzed in MNova software.

### 4.17 High Resolution Mass Spectrometry (HR-MS) – Applicable to Analysis of Monomers

Diepoxide and diamine structures were confirmed by mass spectrometry. The analysis was completed using a positive-ion mode electrospray ionization with an LTQ Orbitrap XL Hybrid FTMS. All *m*/*z* reported include Na+ ion. Analysis were completed by Old Dominion University.

### 4.18 HR-MS – Applicable to Analysis of Degradation Products

Masses of the precipitated and dried E4-P4 degradation products were assessed using electrospray ionization (ESI)-mass spectrometry (MS) on a Thermo Scientific Orbitrap Exploris 240. The sample was solubilized in 50:50 water:acetonitrile containing 0.1% formic acid and diluted to a final concentration of 1 μg/mL prior to direct liquid infusion.

### 4.19 Gel Permeation Chromatography (GPC)

Precipitated and dried aliquots taken throughout E4-P4 glycolysis were dissolved at 1 mg/mL concentration in BHT-stabilized tetrahydrofuran. The E4-P4 degradation products were analyzed using a Tosoh ECOSEC HLC-8320 GPC with tetrahydrofuran as the eluent at 0.350 mL/min flow rate through both reference and sample pumps and a column oven temperature of 40 °C.

### 4.20 Melt Point Determination via DSC

Diepoxide and diamine melting points were determined using a TA Instruments Discovery 250 DSC. 5–10 mg of each monomer was added to an aluminum hermetic pan and subjected to a single temperature ramp at 10 °C/min. Data were analyzed in TA Instruments Trios software.

### 4.21 *E*_a_ Determination via DSC

Stoichiometric amounts (1:1 epoxide functional group to amine hydrogen) of diepoxide and diamine in solid form were pulverized via mortar and pestle and agitated (2 g cumulative scale). Single temperature ramp experiments were performed using 7.5–8.0 mg of the evenly distributed solid components in aluminum hermetic pans. *E*_a_ was determined via the Kissinger method using data obtained at 1.0, 2.5, 5.0, and 10.0 °C/min heating rates. Data were analyzed in TA Instruments Trios software.

### 4.22 Extent of Cure via DSC

To determine initial polymerization enthalpies, the 1.0 °C ramp rate curves from the *E*_a_ experiments were repurposed. For additional replicates of initial polymerization enthalpy evaluation, components were premixed to the point of optical clarity then abruptly cooled; ∼10 mg was added to DSC pans and a 1.0 °C ramp rate was used with a temperature range of 40 to 260 °C. To determine residual enthalpies, ∼10 mg sections were cut from molded parts of each network formed using the cure protocols described in the network synthesis section and added to DSC pans. A 1.0 °C ramp rate and a temperature range of 40 to 260 °C were also used for residual enthalpy determination. Extent of cure was calculated as 100–100 (residual enthalpy/initial polymerization enthalpy) as described by the TA Instruments report titled “Characterization of the Degree of Cure of Thermosetting Resins by DSC.” Data were analyzed in TA Instruments Trios software.

### 4.23 NIR Spectroscopy

A Thermo Scientific Antaris II spectrometer equipped with an InGaAs 2.6 μm detector and a CaF_2_ beamsplitter was used to monitor network cure. Epoxy and amine components were agitated magnetically in a 20 mL vial at about 3.0 g cumulative scale and added onto a glass slide or into a 2-dram vial. Periodically, scans were taken throughout the cure profile. A gold backing plate was placed over the glass slide to limit noise. The scan range was 8000–4000 cm^–1^, the number of scans was 32, and the resolution was 2.000 cm^–1^. Data were analyzed in Thermo Scientific OMNIC software and in OriginPro.

### 4.24 Mid-IR Spectroscopy

A Thermo Scientific Nicolet iS50 spectrometer (deuterated triglycine sulfate (DTGS) detector, KBr beamsplitter) and a Pike GladiATR capable of operation at ambient to 210 °C were used to qualitatively investigate hydrogen bonding. The number of scans was 48 and the resolution was 4 cm^–1^; background scans were taken at each increment of temperature prior to sample analysis. Samples were flexural specimens previously dried for 2 h at 150 °C.

### 4.25 Thermogravimetric Analysis

Network thermal stability was analyzed on a TA Instruments Discovery 550 TGA using platinum HT pans and samples of ∼ 15 mg. Under nitrogen flow, the temperature was ramped at 10 °C/min from ambient to 800 °C. Tests were run in duplicate. Data were analyzed in TA Instruments Trios software.

### 4.26 Dynamic Mechanical Analysis

Network thermomechanical behavior was evaluated via strain-controlled DMA performed on a TA Instruments Discovery DMA 850 operating in tension mode. Sample dimensions were about 30.0 mm × 5.00 mm × 1.00 mm, and the initial gap from stationary to mobile clamp was set to 10.0 ± 0.1 mm. The experiments consisted of temperature ramps from +30 °C to +260 °C (or as high as +300 °C dependent on network) or from −140 °C to +200–300 °C (upper bound dependent on network) using a 3.0 °C/min ramp rate, a frequency of 1.0 Hz, a strain of 0.1%, and a preload force of 0.01 N. Tests were run in duplicate and data are reported as average ± standard error. Postwater immersion data was obtained using the same conditions except an initial temperature of +40 °C. Data were analyzed in TA Instruments Trios software.

### 4.27 Tensile Properties

Network mechanical properties in tension were evaluated at ambient temperature on an MTS Insight electromechanical load frame equipped with a 2.5 kN load cell using a 1 mm/min crosshead speed and type V tensile bar geometry as per ASTM D638 with 1.3 ± 0.1 mm thickness (sanded to rectangular gage cross section). Tests were run in triplicate and data are reported as average ± standard error.

### 4.28 Flexural Properties

Network flexural properties at ambient temperature were also evaluated on an MTS Insight electromechanical load frame using specimens sanded to rectangular geometry and 1.60 ± 0.05 mm thickness × 12.7 mm width × 25.4 mm length. The load frame was equipped with a 500 N load cell and a three-point bend fixture. A strain rate of 0.10 mm/mm/min was used to a limit of 0.10 mm/mm (procedure B of ASTM D790 except 2x the strain endpoint). Toughness was calculated by calculation of the area under the stress/strain curve. Tests were run in sextuplicate and data are reported as average ± standard error.

### 4.29 Plane-Strain Fracture Toughness

Specimens of 6.6 mm thickness (B) by 15 mm height (W) by 76 mm length were formed and sanded to rectangular dimensions. Central notches were cut via a 1/8″ fluted bit, and fresh razor notches were cut into the notch bases of each specimen with sliding motion. An a/W of 0.47 was used, where a = precrack length inclusive of machined notch and razor-cut notch. An MTS Insight electromechanical load frame equipped with a 500 N load cell was used with a three-point bend fixture and a support span of 62 mm. Tensile yield stresses were input into the validity criterion described in section 7.1.2 of ASTM5045-14 and sufficiency of the specimen ligament (W-a) size was established so conditional *K*_C_ values were translated to *K*_IC_ values in all cases. Although ASTM D5045 is based on linear elastic fracture mechanics, limited nonlinearity is permissible provided that small-scale yielding criteria are satisfied. Compliance for the polyester thermosets fell within criteria established by ASTM D5045, and part dimensions were such that plane strain conditions were met.^71,91^ Tests were run in triplicate and data are reported as averages, where the error bars indicate standard error.

### 4.30 Impact Resistance

Panels of 100 × 100 × ca. 3.6 mm thickness were formed in HT silicone molds and planed/sanded to rectangular dimensions (final panel thickness 3.4 ± 0.2 mm). Prior to those operations, some curvature was apparent for DGEBA – DDS type panels (Although polyester panels were relatively flat, they were still planed/sanded to copy the process used for reference specimens). Impact resistance of the panels was determined on Instron Dynatup 9250HV frame with a 15.6 kN 10 mm diameter radiused impactor. The impact energy was fixed at 20.0 J. Load vs deflection curves were integrated as per ASTM D3763-23 to determine puncture energy. In all cases, specimens were fully penetrated. Tests were performed in triplicate and data are reported as averages, where the error bars indicate standard error.

### 4.31 SEM

Fractured SENB specimens were cut to ca. 7 mm roughly parallel to the fracture surfaces so that it was possible to fit the specimens into the SEM analysis chamber. Scanning electron microscopy was performed using a Zeiss Sigma VP FEGSEM at an accelerating voltage of 20.00 kV and an NTS-BSD (detector).

### 4.32 Single Lap Shear

Lap shear tests were performed in accordance with ASTM D1002 on Alloy 2024 aluminum panels (Q-Panel AR-14) cleaned with isopropanol prior to application of adhesive. The standard 25.4 × 12.7 mm overlap/bond area was used, and aluminum tabs were adhered to either end of the panels on opposite sides to maintain the centrality of the bond line in the test. Epoxy/amine adhesive was preheated to 120 °C and added to a preheated aluminum bottom panel on an alignment device used for assembly to limit bending moments of the assembled specimens under tension. A preheated top panel was added and 15 mm binder clips were used to apply pressure; excess adhesive (observed in all cases as joints were purposefully overfilled) was removed, and the assembled specimens were run through the cure protocol described in network formation section. An MTS Insight electromechanical load frame equipped with a 10 kN load cell using a 1.3 mm/min crosshead speed was used. Tests were run in sextuplicate and data are reported as averages, where the error bars indicate standard error.

### 4.33 Peel Resistance (T-peel)

Epoxy and amine components (∼16 g cumulative scale) were degassed in separate containers and agitated magnetically at about 120 °C to the point of optical clarity. 3 M K1 glass microspheres (median particle size = 65 μm, maximum particle size = 120 μm) were added at 5 vol % to regulate bond line thickness. Microglass-filled adhesive was applied along 9″ of the length of a 12 × 4″ preheated, isopropanol-cleaned 0.025″ aluminum panel via a preheated 25 mil applicator. A preheated top panel of the same type was added, and 1/8″ PTFE spacer plates and 3/4″ aluminum plates (to apply pressure to the joint) were added to the top/bottom. The material underwent cure as described previously. Cured specimens were cut into 1″ widths on a table saw equipped with a nonferrous blade, with the outer ∼3/8″ along the length discarded. The nonbonded panel sections at the end of each 1″ strip were bent 90° upward and downward from the plane of the bonded section to form a T-shape, in accordance with ASTM D1876. Load was applied at a crosshead speed of 10″ per minute via a Mark 10 electromechanical load frame equipped with a 250N load cell. Tests were run in sextuplicate and data are reported as averages, where the error bars indicate standard error.

### 4.34 Network Glycolytic Degradation

Disks of about 14 mm × 1 mm (0.21 ± 0.005 g each) were submerged in 21 ± 0.1 g ethylene glycol with 1 wt % zinc acetate in needle-vented 40 mL vials. The degradation solution temperature was maintained at roughly 190 °C under agitation. The disks were weighed at intervals to establish weight loss vs time plots. Tests were run in triplicate and data reported are averages.

### 4.35 Water Pickup

Rectangular specimens of 0.43 ± 0.02 g and 1.6 mm thickness predried at 160 °C for 4 h were weighed then immersed in DI water and at specified intervals blotted dry, reweighed, and reimmersed. Tests were run in triplicate and data reported are averages, where the error bars indicate standard error.

### 4.36 Weatherability

Formed network parts underwent 100 h of exposure in a Q-Sun model Xe-3 Xenon test chamber following ASTM G155-21 cycle 1 (102 min full spectrum light, 18 min water spray/full spectrum light in alternation). Two DMA bars and three tensile specimens for each network type were exposed and evaluated post exposure via the previously described procedures. Data are reported as averages, where the error bars indicate standard error.

## Supplementary Material



## Notes

^1^H and ^13^C NMR spectra of all monomers, monomer precursors; ^1^H NMR spectra of monoepoxide/monoamine compounds used in a study of the potential for amidation to occur under thermoset cure conditions, and the product of the monoepoxide/monoamine reaction; a scheme of potential amidation side reaction; comparative polymerization enthalpy data for E4-P4 and DGEBA-4,4′-DDS systems; comparative extent of cure data for all systems; overlaid NIR spectra for all systems through the polymerizations; integral values and peak maxima for secondary relaxations; mass densities, theoretical maximum cross-link densities, and values related to theoretical maximum cross-link density calculations for all systems; theoretical functional group concentrations and related values for all systems; overlaid mid-IR spectra from temperature sweep experiments on E4-P4 and Epon 828–4,4′-DDS systems as qualitative assessment of hydrogen bonding; fits and residuals for mid-IR hydroxyl peak deconvolution for quantitate assessment of hydrogen bonding at ambient temperature. The following files are available free of charge. Brief description (PDF)
