# Supplementary material for: Epoxy–Amine Route to Tough and Degradable Aromatic Polyester Thermosets
Source: ACS Appl Polym Mater. 2025 Nov 26;7(23):16173–93. doi: 10.1021/acsapm.5c03377 (PMC12706740; doi:10.1021/acsapm.5c03377)
Supplement: Supplementary file 1 [file ap5c03377_si_001.pdf]

Supporting Information

Epoxy–Amine Route to Tough and Degradable Aromatic Polyester Thermosets

*Jeffrey Aguinaga,[1] Richard C. Ferguson, [1] Michael Blanton, [1] Windfield S. Swetman, [1] James W. Rawlins, Tristan D. Clemons, [1] Travis Thornell, [2] and Derek L. Patton[1]**

1 School of Polymer Science and Engineering, University of Southern Mississippi, Hattiesburg, MS 39406, USA

2 Geotechnical and Structures Laboratory, Engineer Research and Development Center, US Army Corps of Engineers, Vicksburg, MS 39180, USA

Corresponding author: derek.patton@usm.edu

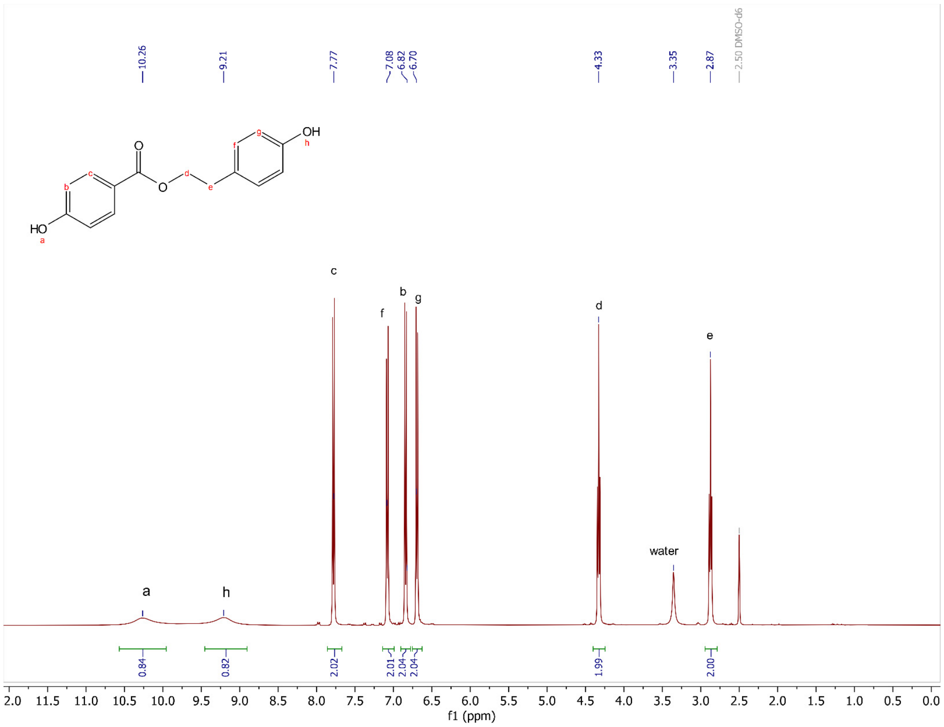

Figure S1. ^{1}H NMR spectrum of 4-hydroxyphenethyl-4-hydroxybenzoate

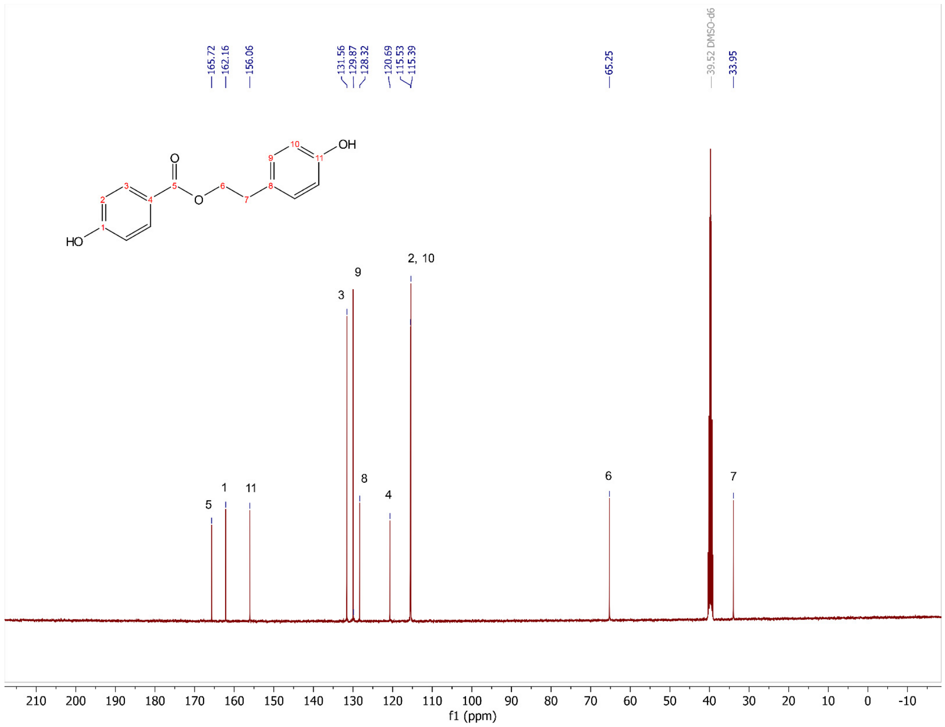

Figure S2. ^{13}C NMR spectrum of 4-hydroxyphenethyl-4-hydroxybenzoate

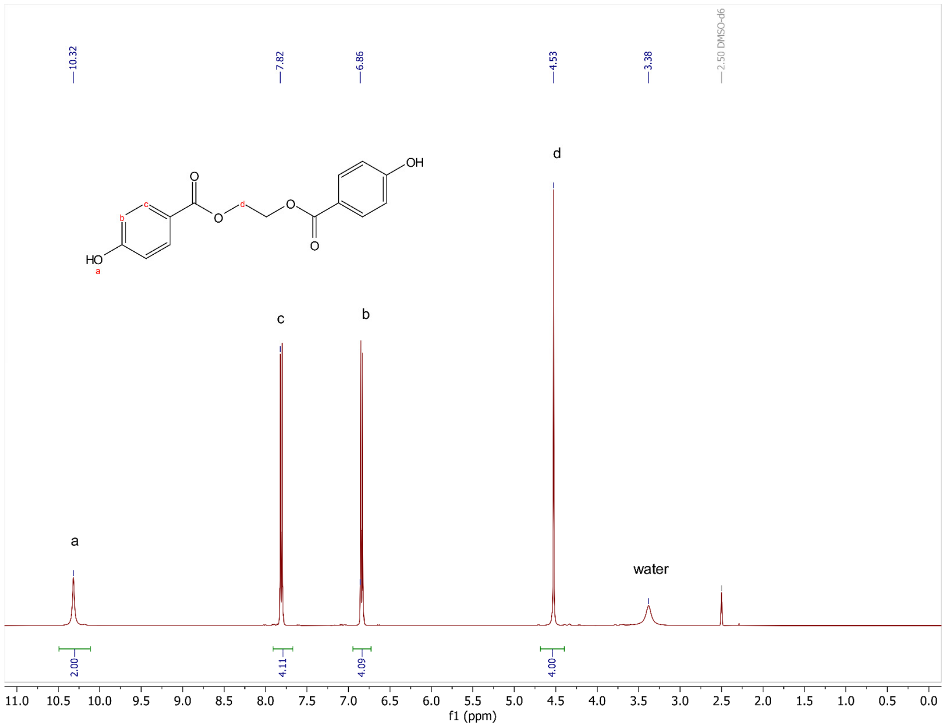

Figure S3. ^{1}H NMR spectrum of ethyl-1,2-bis-4-hydroxybenzoate

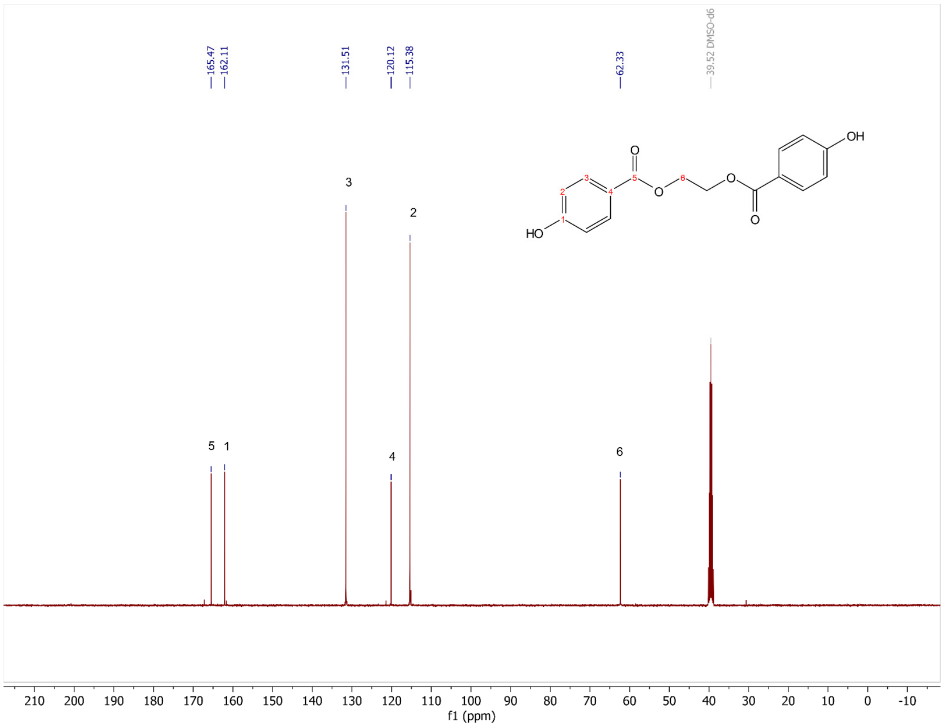

Figure S4. ^{13}*C NMR spectrum of ethyl-1,2-bis-4-hydroxybenzoate*

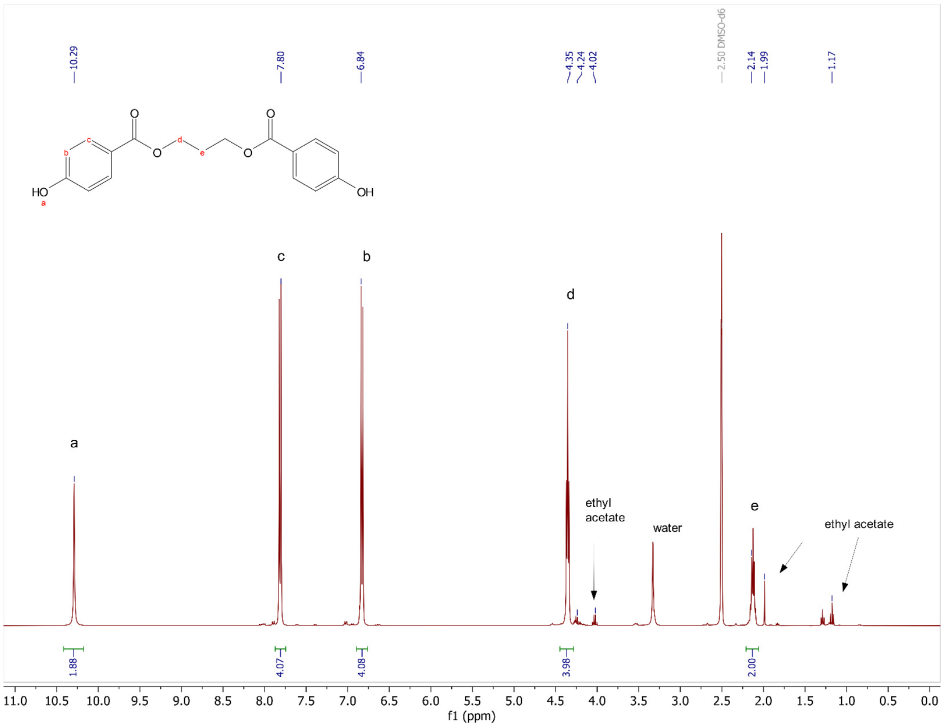

Figure S5. ^{1}*H NMR spectrum of propyl-1,3-bis-4-hydroxybenzoate*

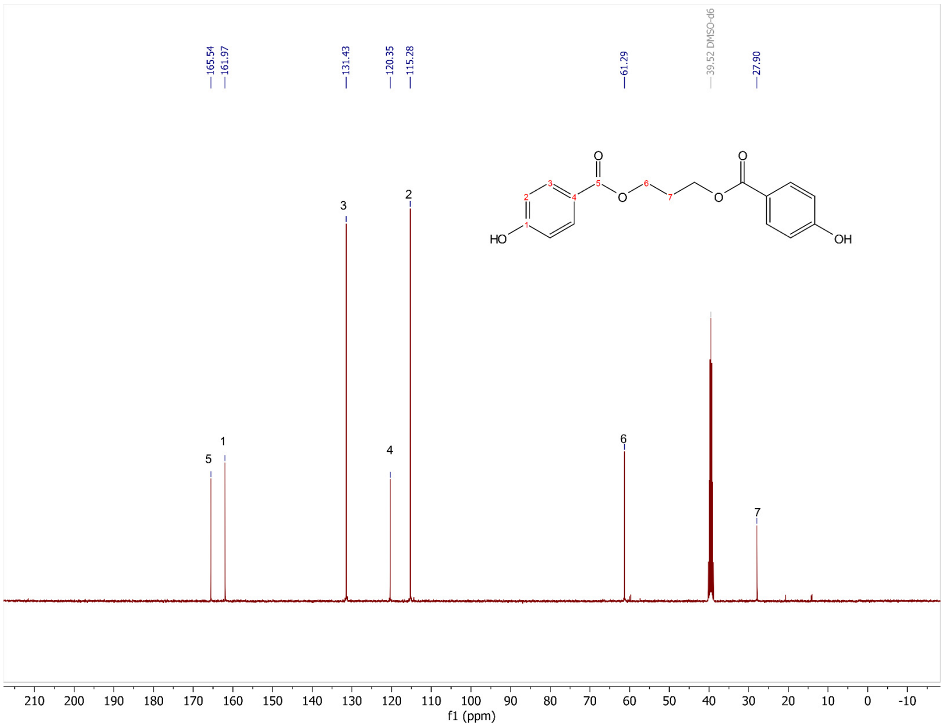

Figure S6. ^13^*C NMR spectrum of propyl-1,3-bis-4-hydroxybenzoate*

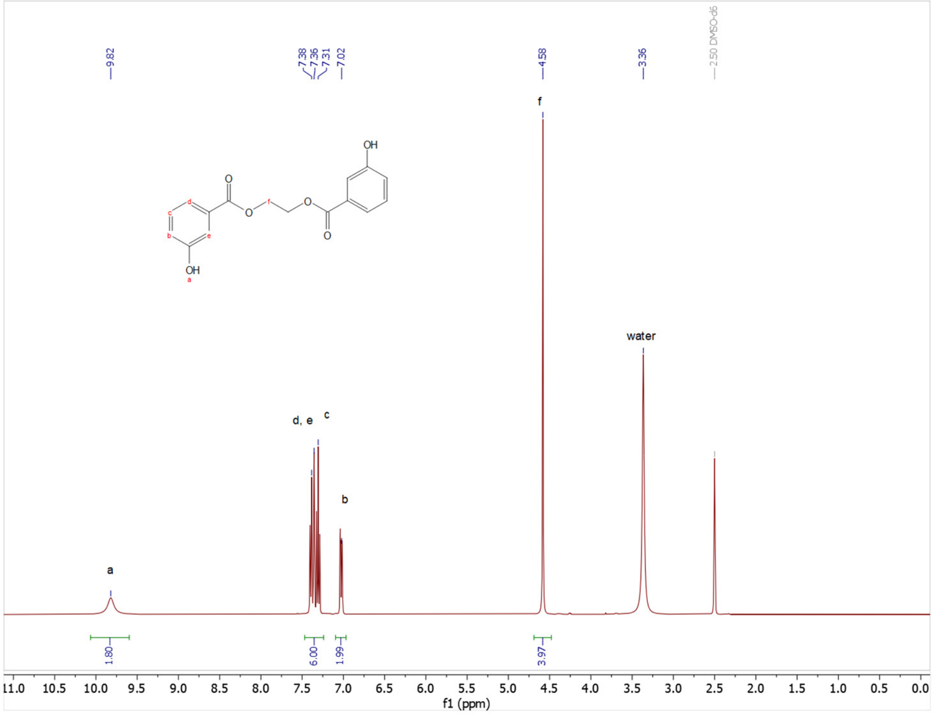

Figure S7. ^1^*H NMR spectrum of ethyl-1,2-bis-3-hydroxybenzoate*

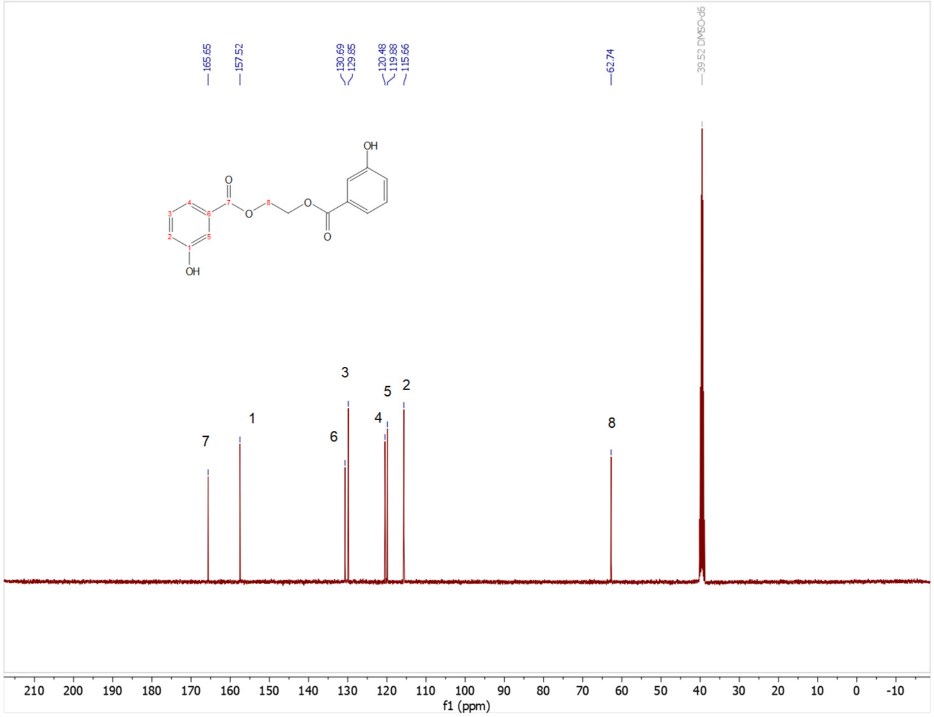

Figure S8. ^{13}C NMR spectrum of ethyl-1,2-bis-3-hydroxybenzoate

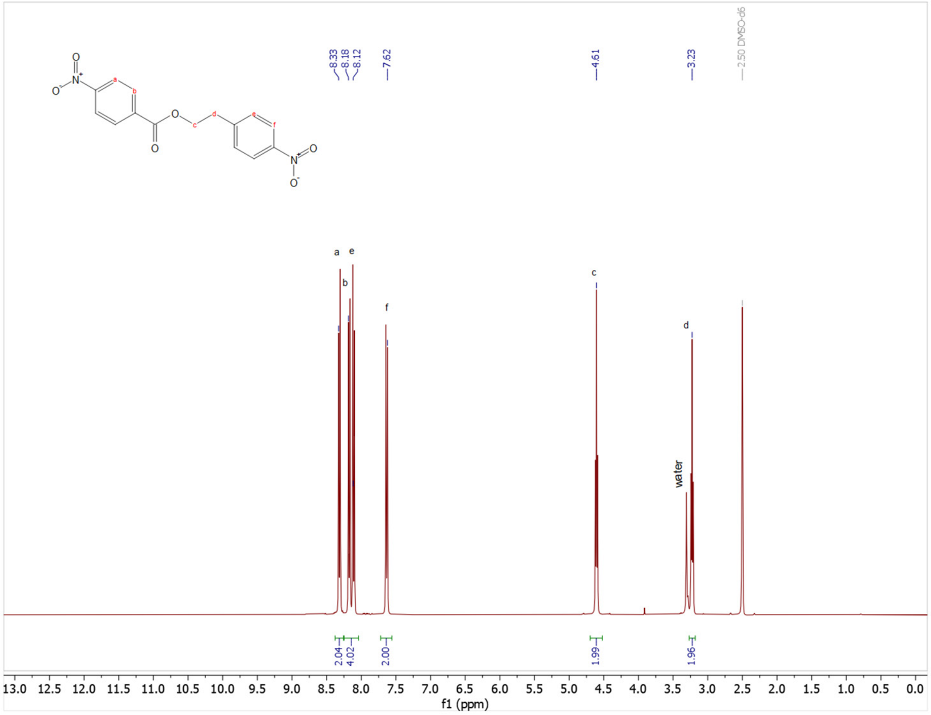

Figure S9. ^{1}H NMR spectrum of 4-nitrophenethyl-4-nitrobenzoate

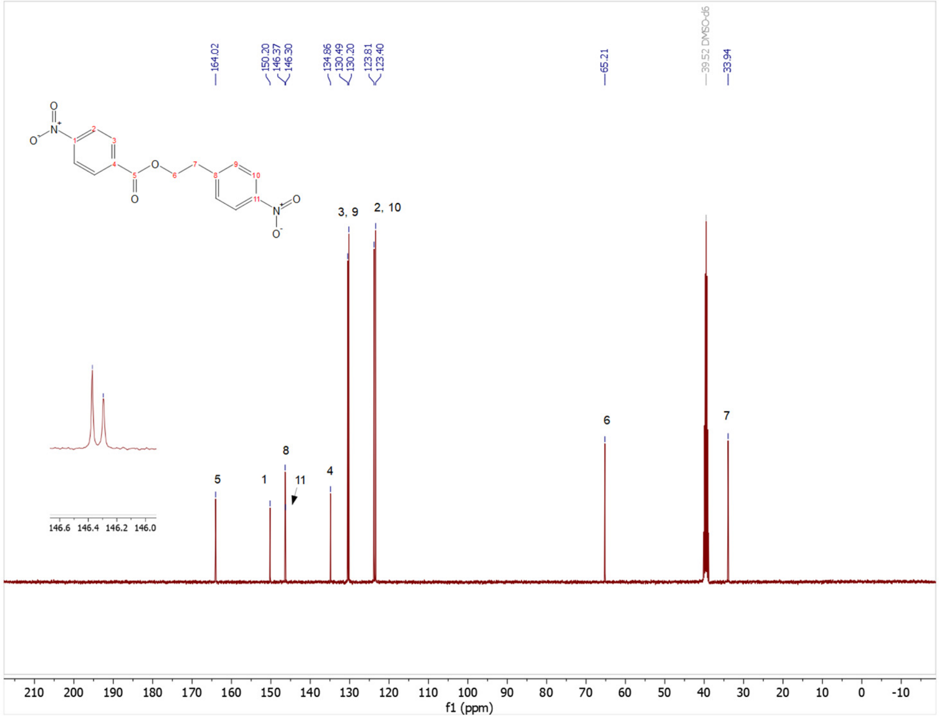

Figure S10. ^{13}C NMR spectrum of 4-nitrophenethyl-4-nitrobenzoate

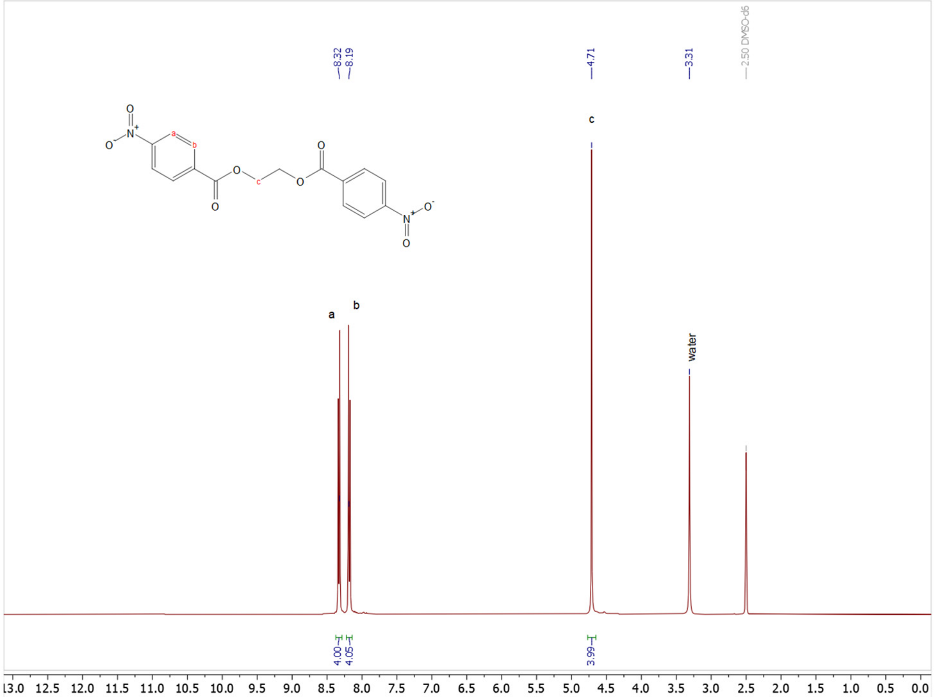

Figure S11. ^{1}H NMR spectrum of ethyl-1,2-bis-4-nitrobenzoate

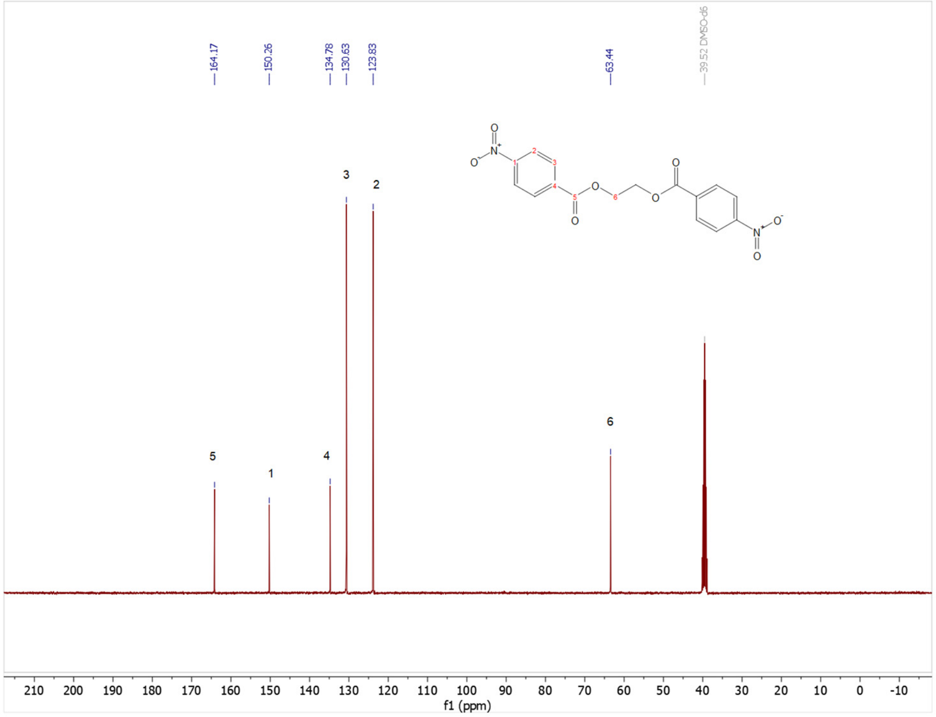

Figure S12. ^{13}C NMR spectrum of ethyl-1,2-bis-4-nitrobenzoate

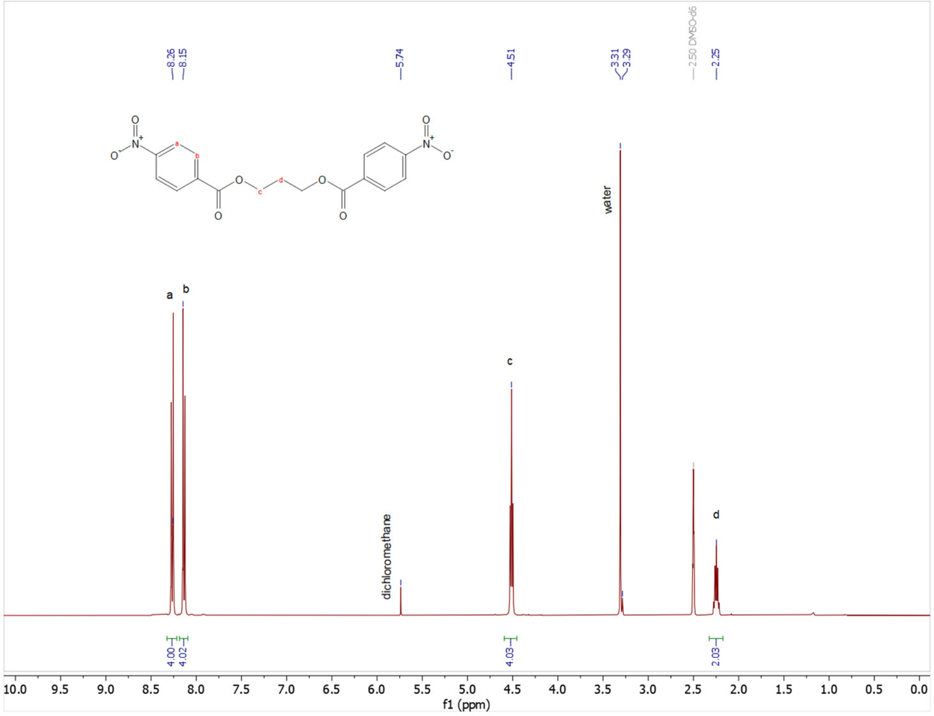

Figure S13. ^{1}H NMR spectrum of propyl-1,3-bis-4-nitrobenzoate

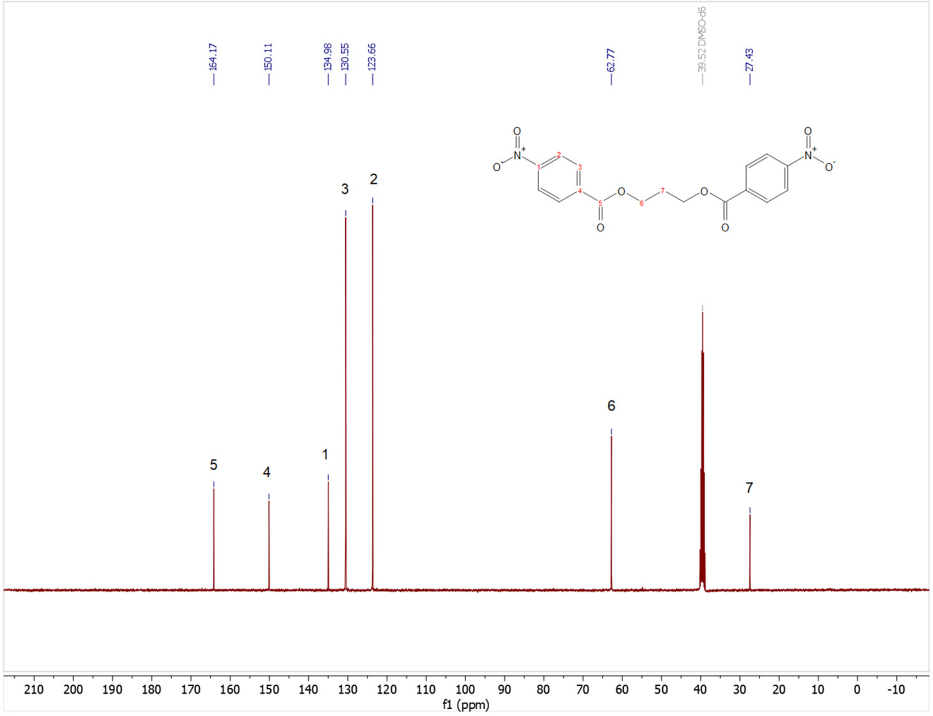

Figure S14. *^{13}C NMR spectrum of propyl-1,3-bis-4-nitrobenzoate*

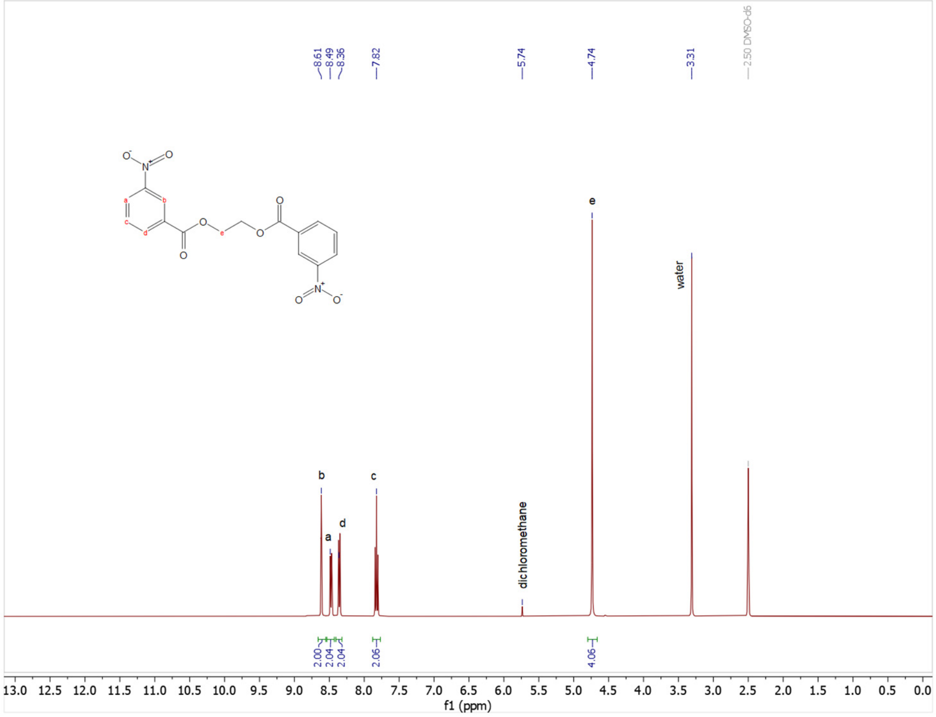

Figure S15. *1H NMR spectrum of ethyl-1,2-bis-3-nitrobenzoate*

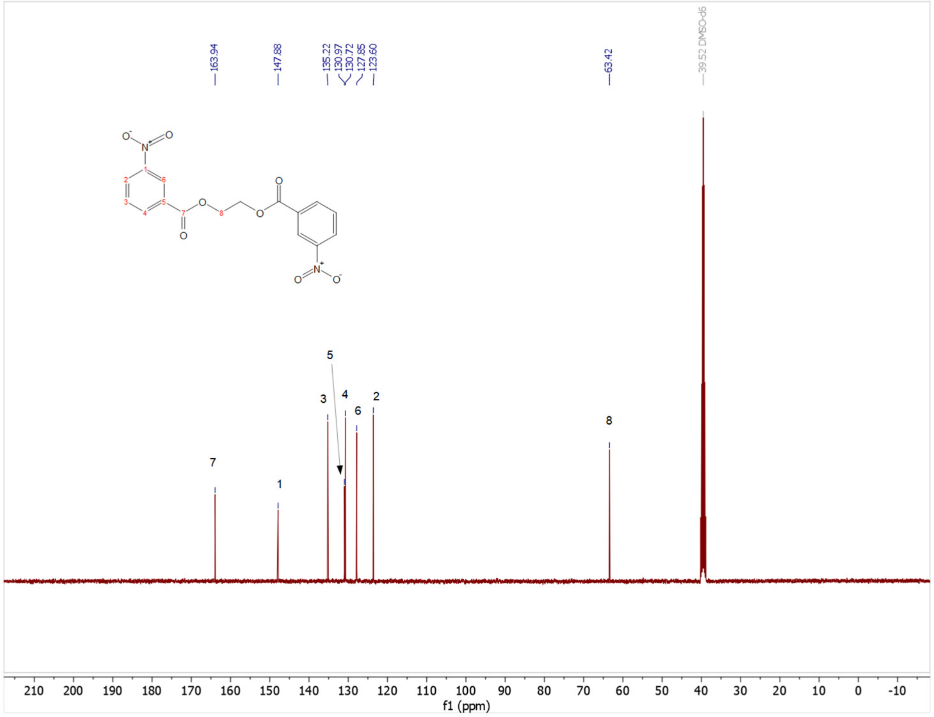

Figure S16. ^{13}C NMR spectrum of ethyl-1,2-bis-3-nitrobenzoate

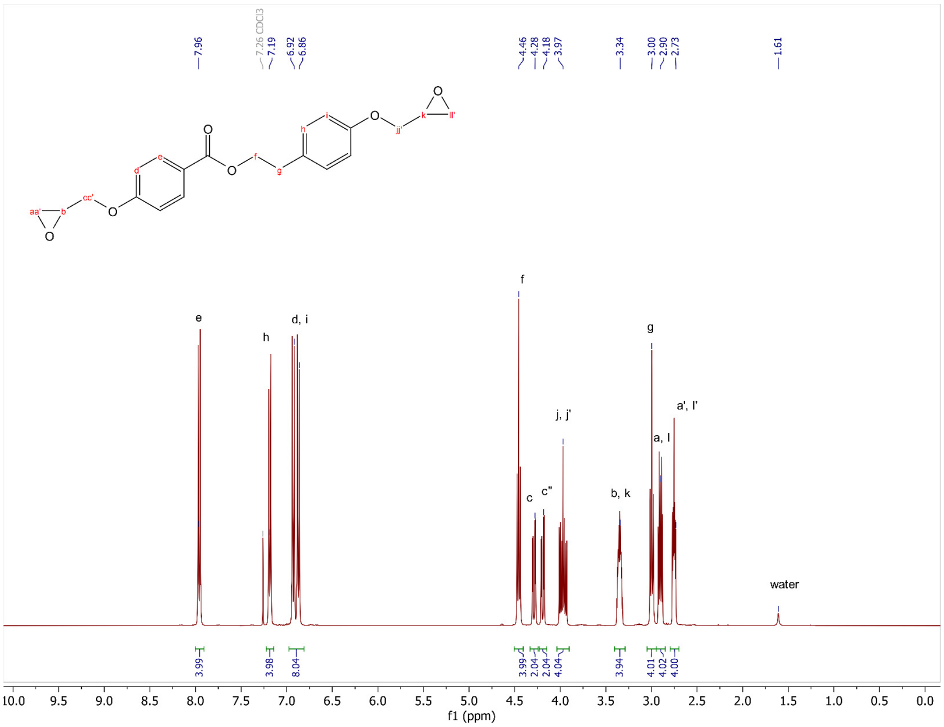

Figure S17. ^{1}H NMR spectrum of 4-glycidyloxyphenethyl-4-glycidyloxybenzoate

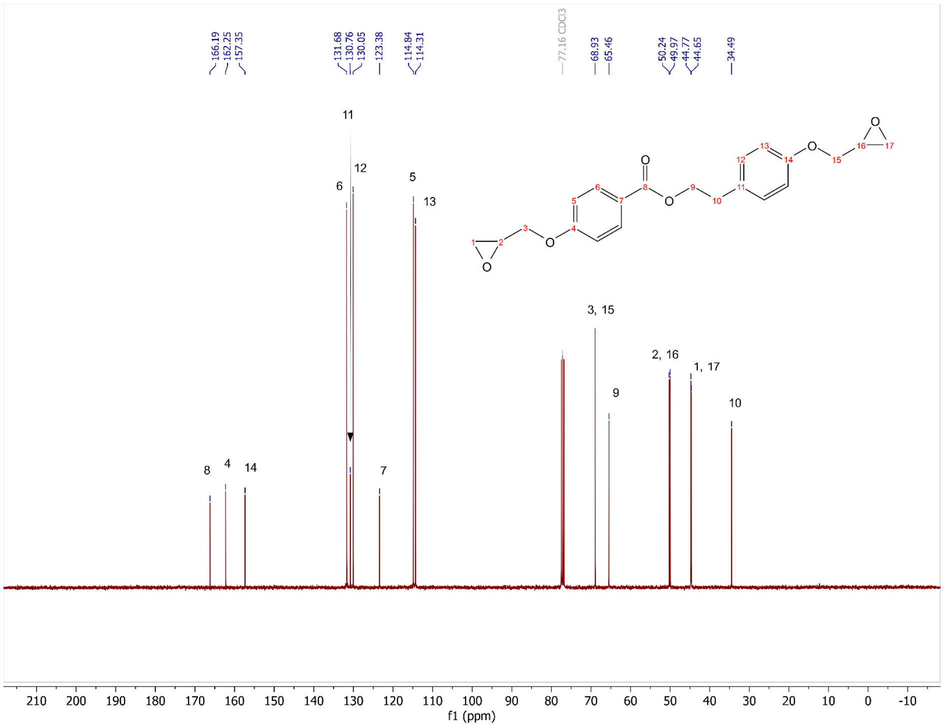

Figure S18. ^{13}C NMR spectrum of 4-glycidyloxyphenethyl-4-glycidyloxybenzoate

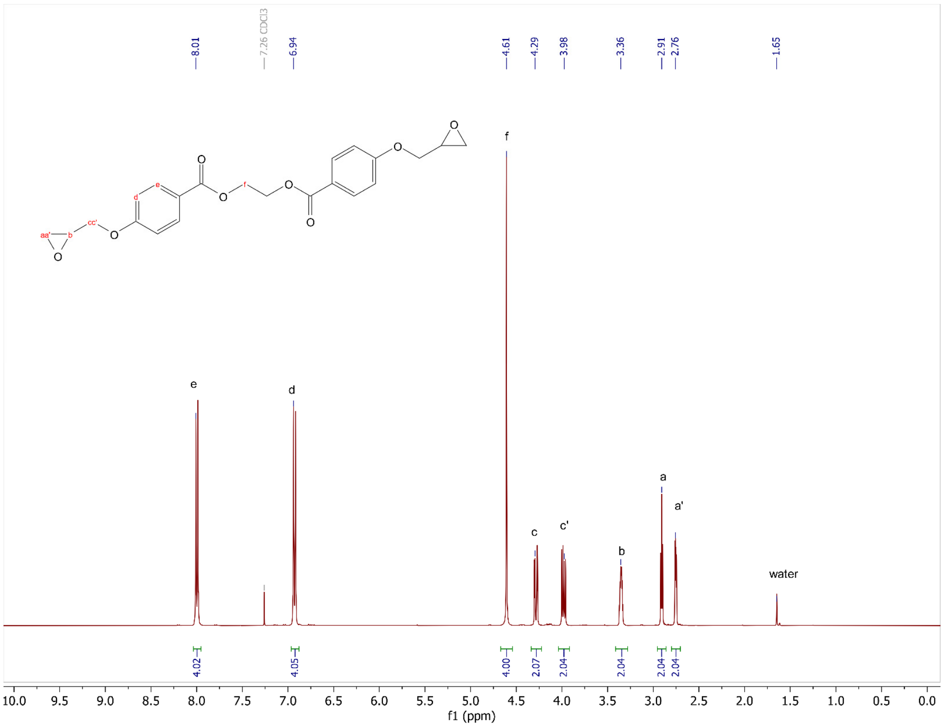

Figure S19. ^{1}H NMR spectrum of ethyl-1,2-bis-4-glycidyloxybenzoate

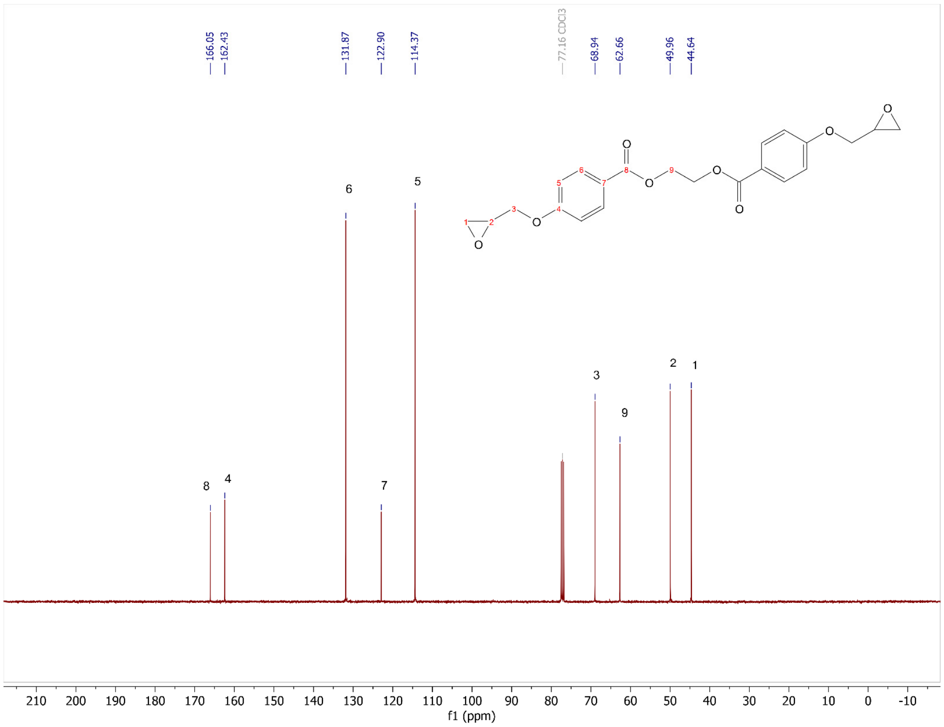

Figure S20. ^{13}C NMR spectrum of ethyl-1,2-bis-4-glycidyloxybenzoate

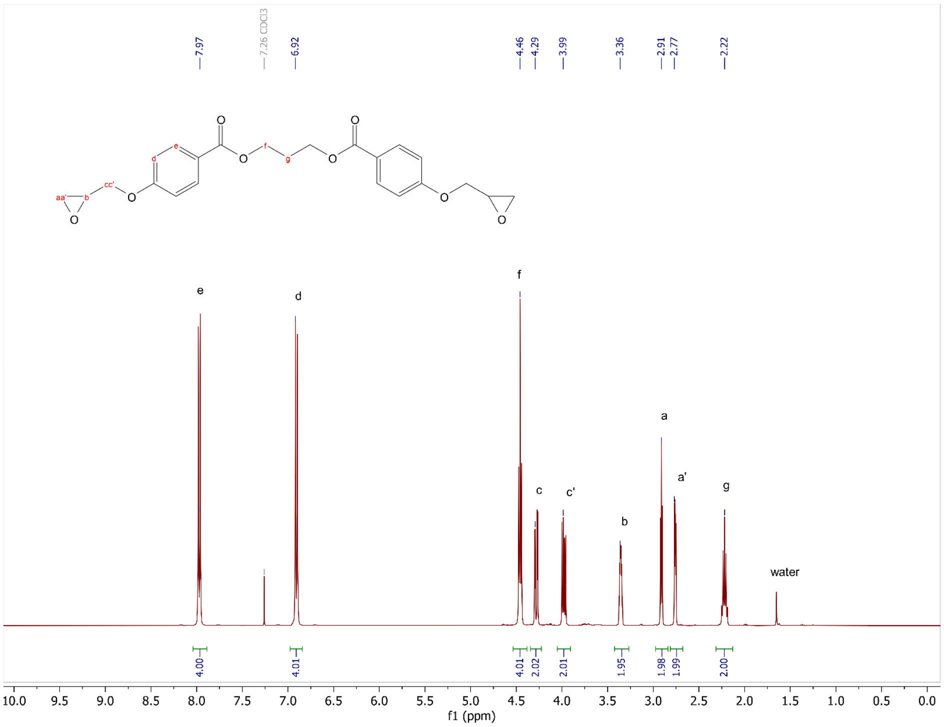

Figure S21. ^{1}H NMR spectrum of propyl-1,3-bis-4-glycidyloxybenzoate

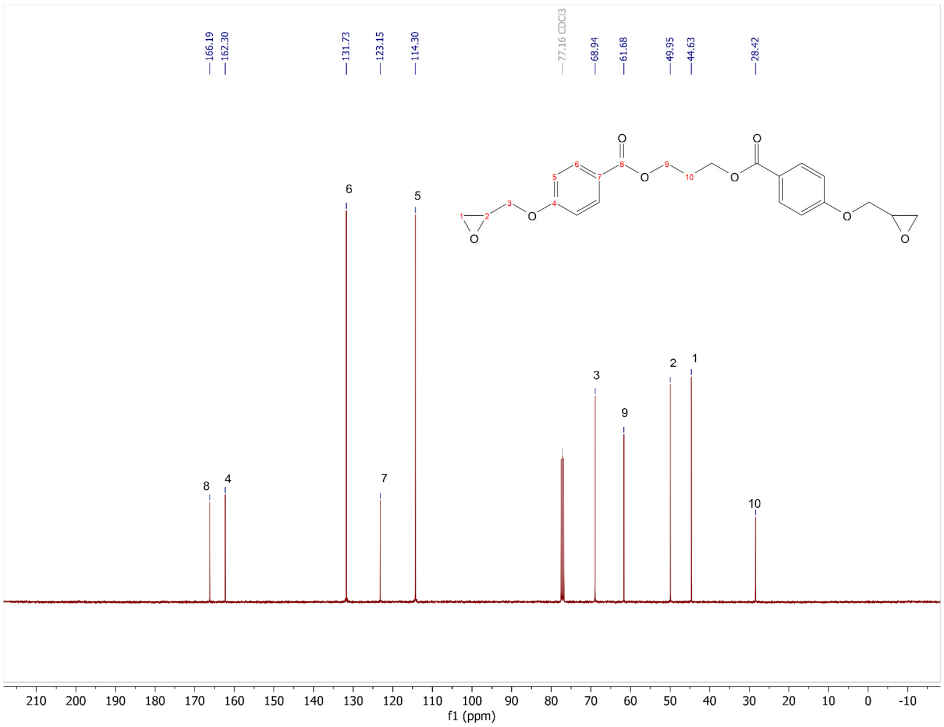

Figure S22. ^{13}C NMR spectrum of propyl-1,3-bis-4-glycidyloxybenzoate

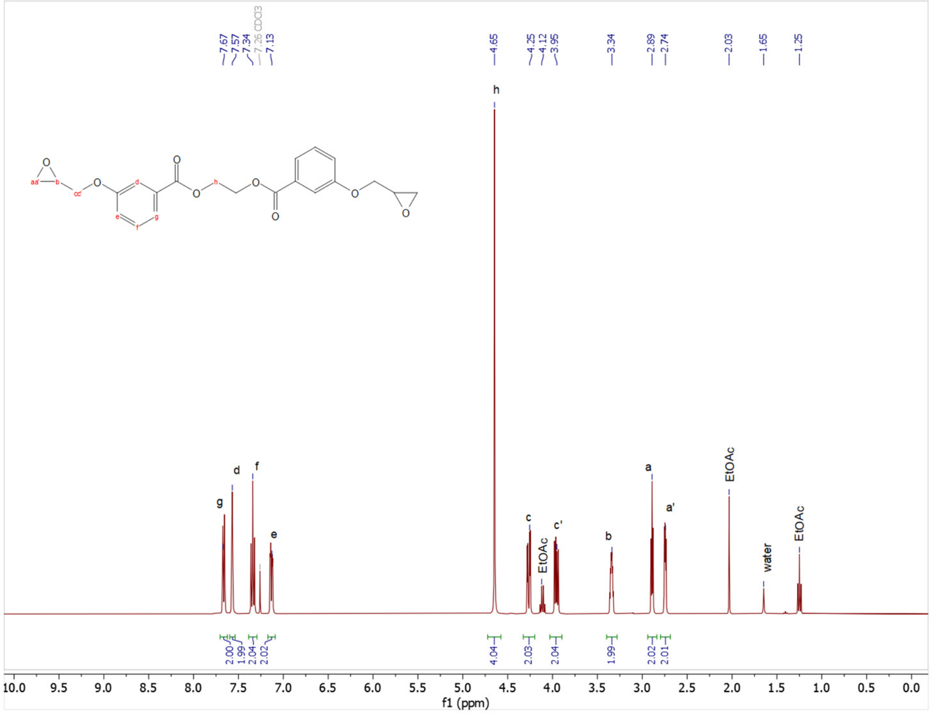

Figure S23. ^{1}H NMR spectrum of ethyl-1,2-bis-3-glycidyloxybenzoate

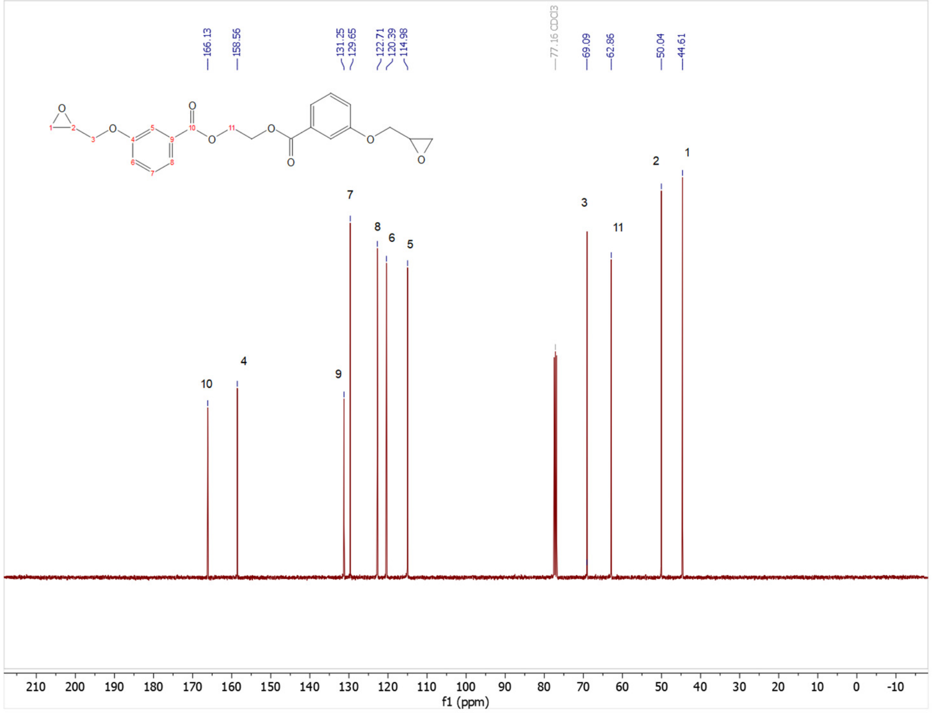

Figure S24. ^{13}C NMR spectrum of ethyl-1,2-bis-3-glycidyloxybenzoate

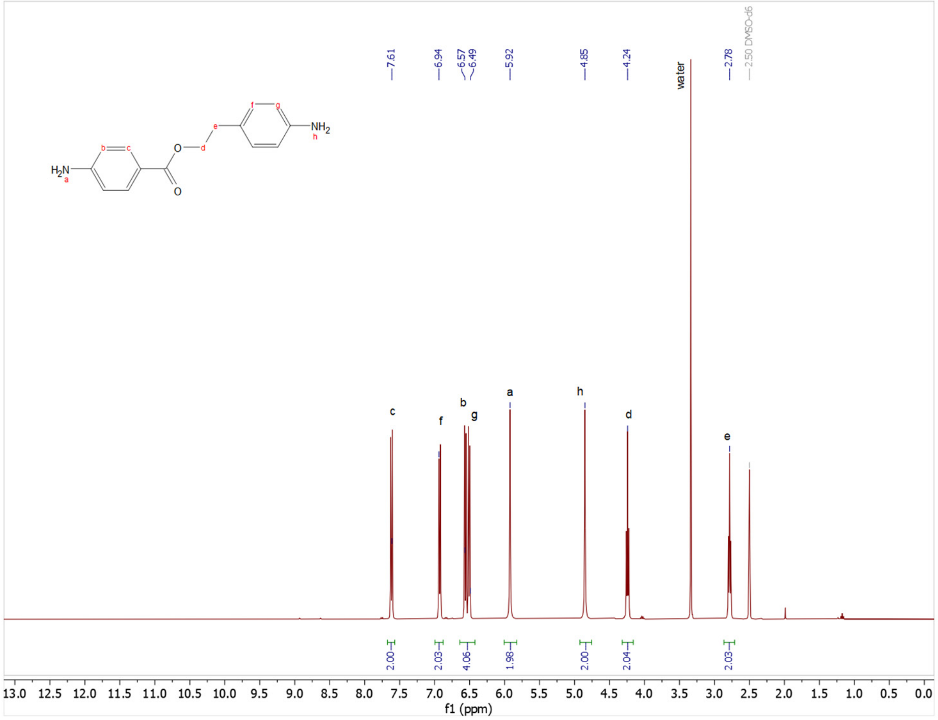

Figure S25. 1H NMR spectrum of 4-aminophenethyl-4-aminobenzoate

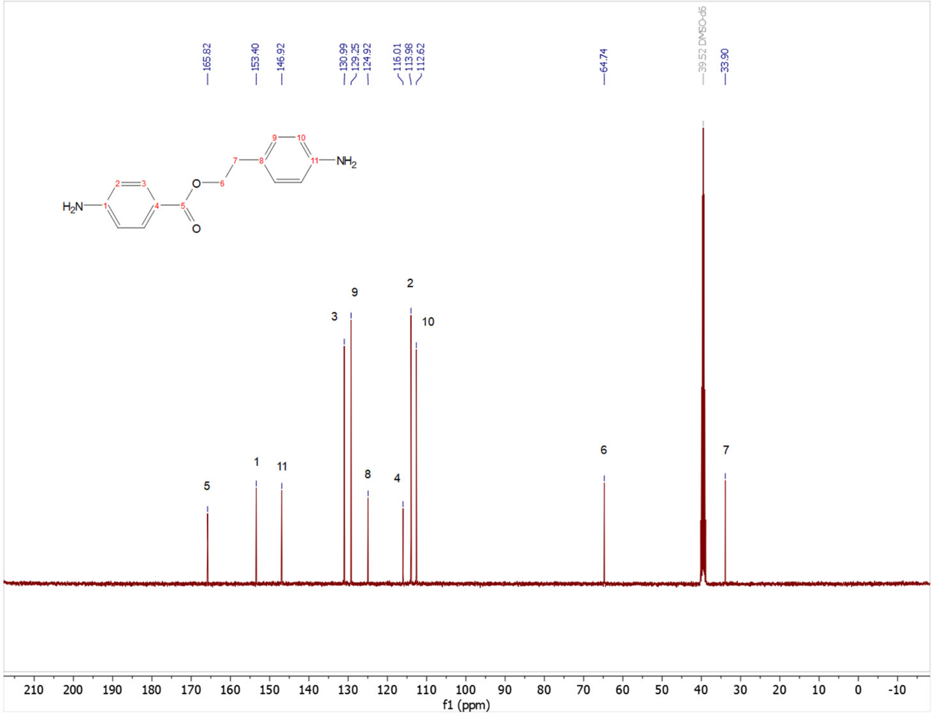

Figure S26. ^{13}C NMR spectrum of 4-aminophenethyl-4-aminobenzoate

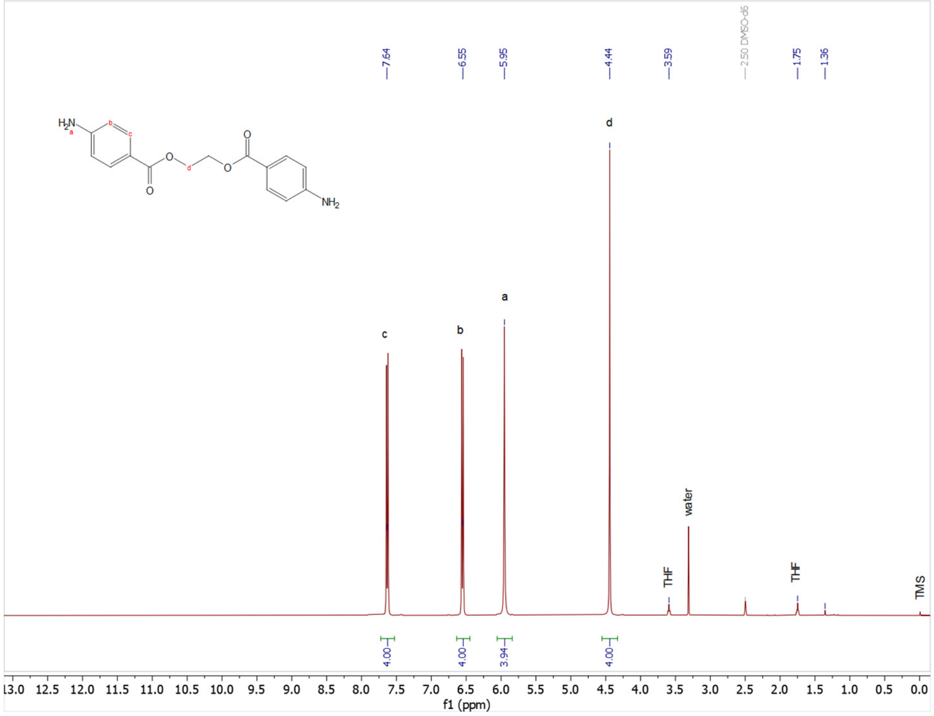

Figure S27. ^{1}H NMR spectrum of ethyl-1,2-bis-4-aminobenzoate

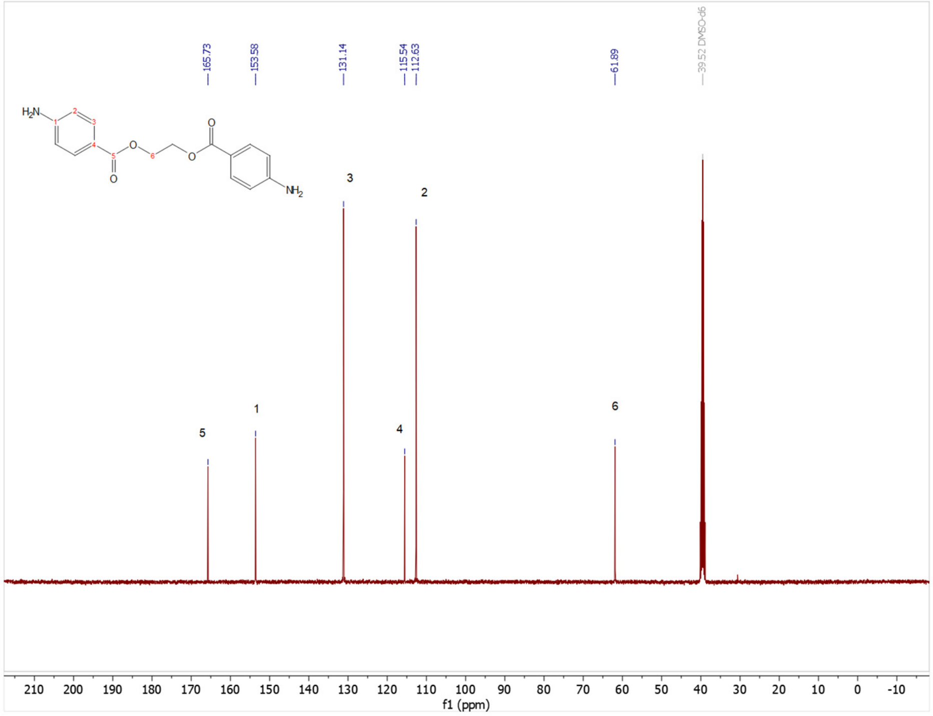

Figure S28. ^{13}C NMR spectrum of ethyl-1,2-bis-4-aminobenzoate

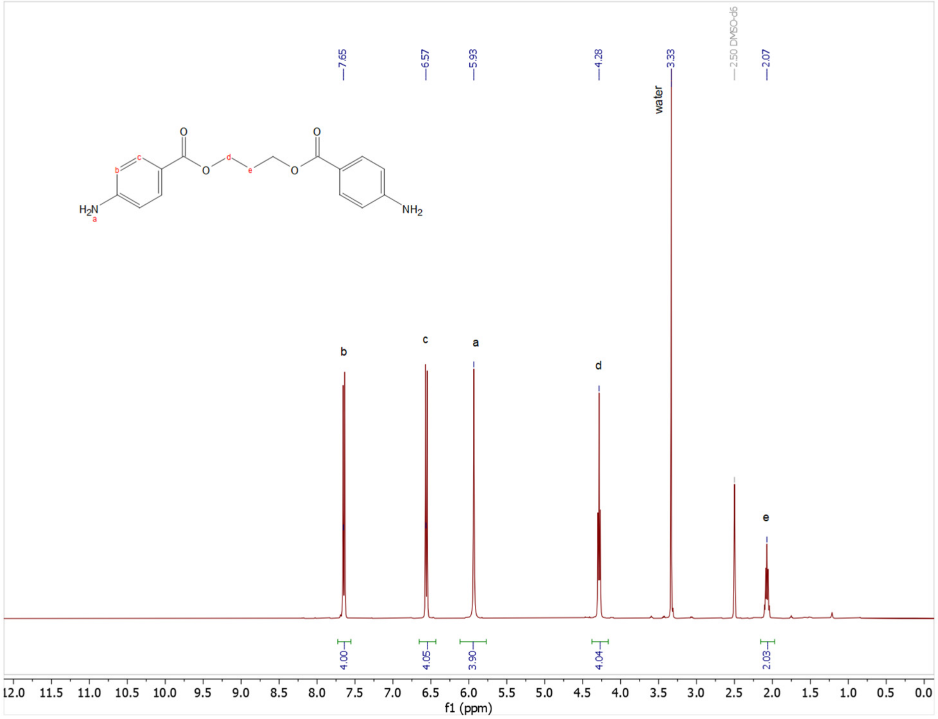

Figure S29. 1H NMR spectrum of propyl-1,3-bis-4-aminobenzoate

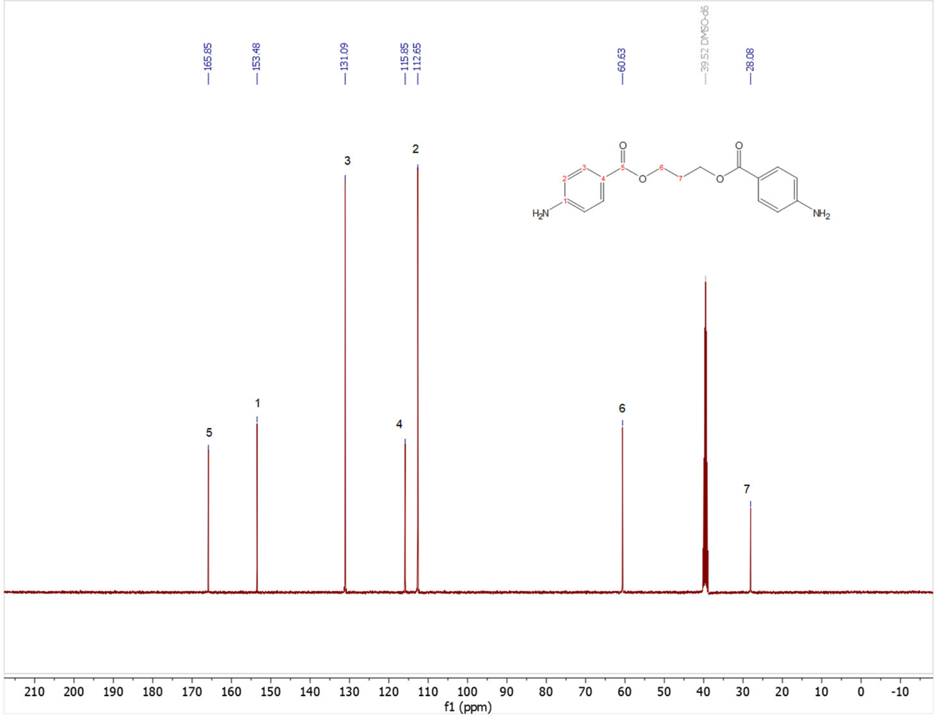

Figure S30. ^{13}*C NMR spectrum of propyl-1,3-bis-4-aminobenzoate*

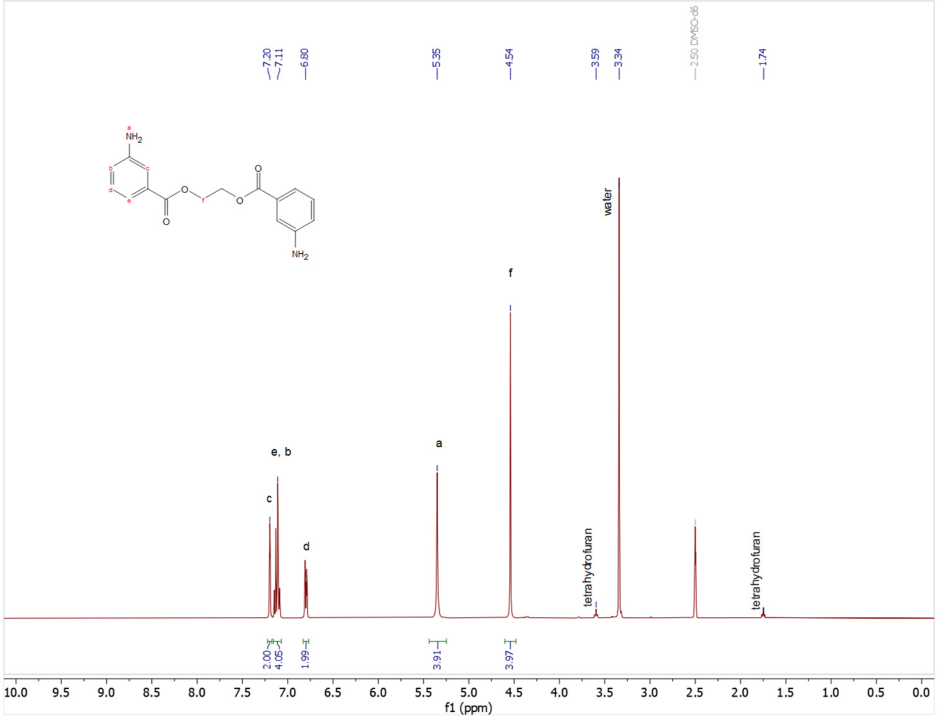

Figure S31. ^{1}*H NMR spectrum of ethyl-1,2-bis-3-aminobenzoate*

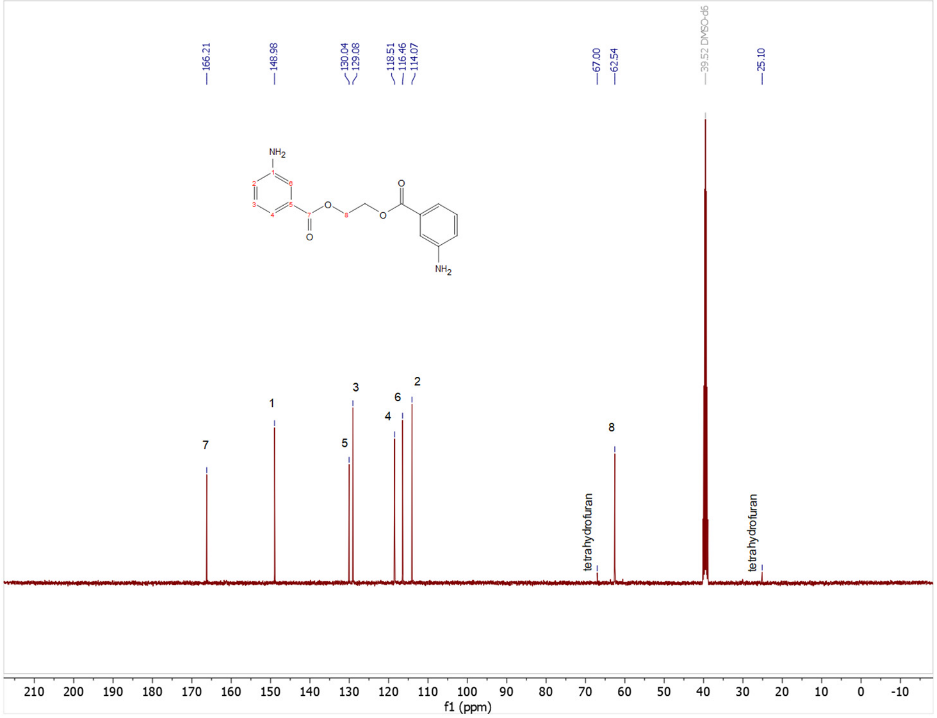

Figure S32. ¹³C NMR spectrum of ethyl-1,2-bis-3-aminobenzoate

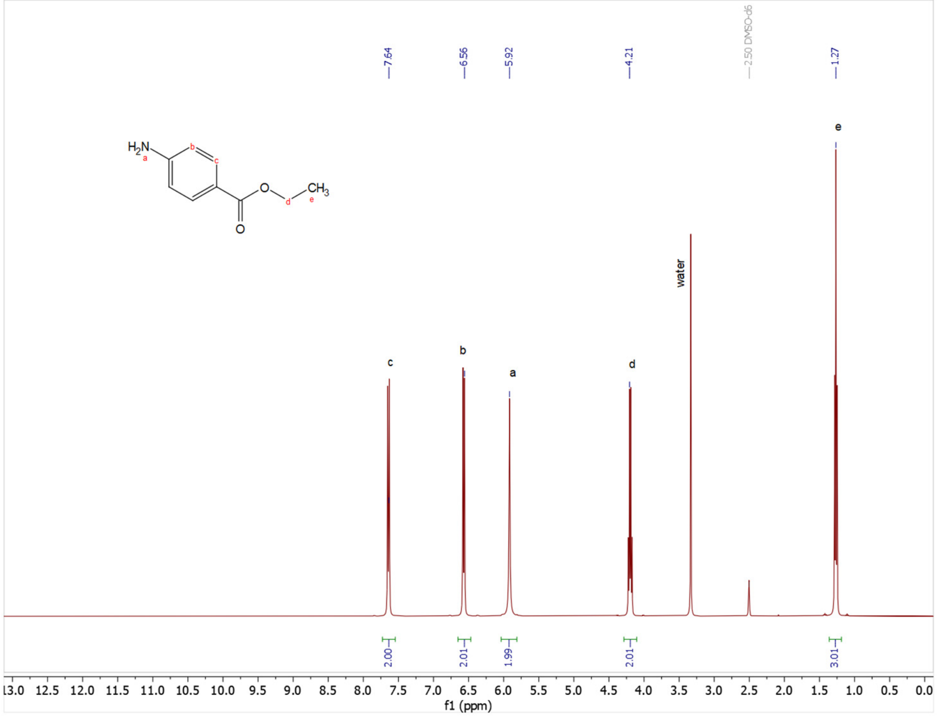

Figure S33. ¹H NMR spectrum of ethyl-4-aminobenzoate (for amidation study)

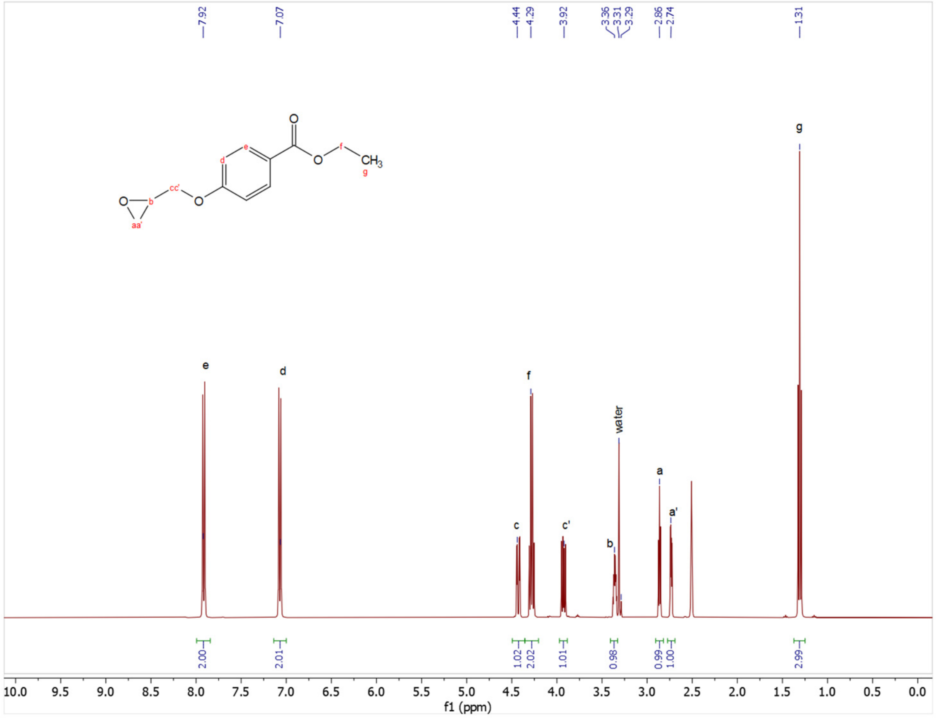

Figure S34. ^{1}H NMR spectrum of ethyl-4-glycidyloxybenzoate (for amidation study)

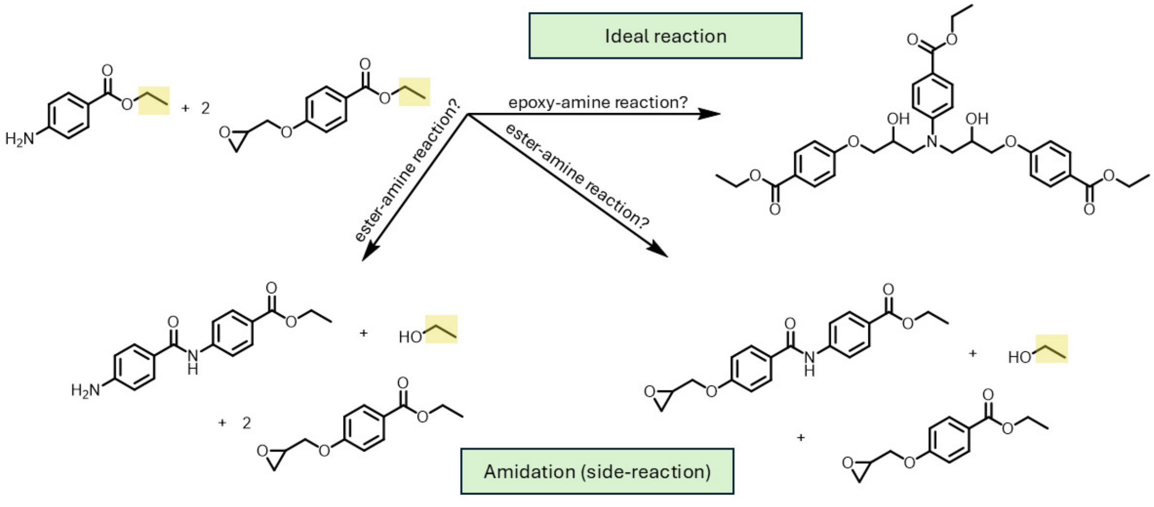

Figure S35. Scheme of potential reactions of ethyl-4-aminobenzoate and ethyl-4-glycidyloxyobenzoate

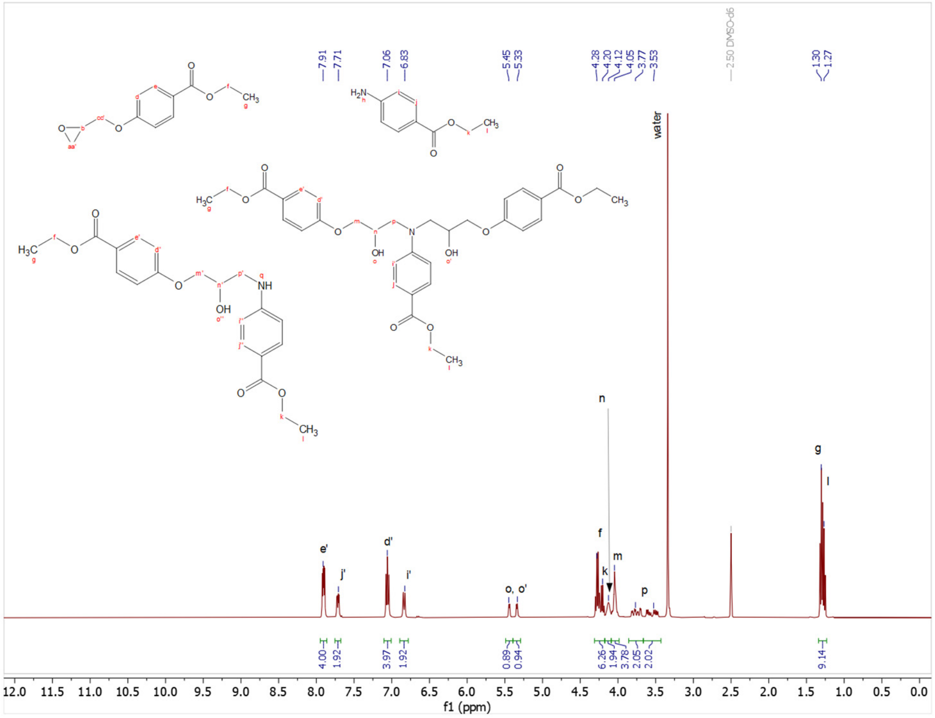

Figure S36. ^{1}H NMR spectrum of ethyl-4-glycidyloxybenzoate/ethyl-4-aminobenzoate adduct

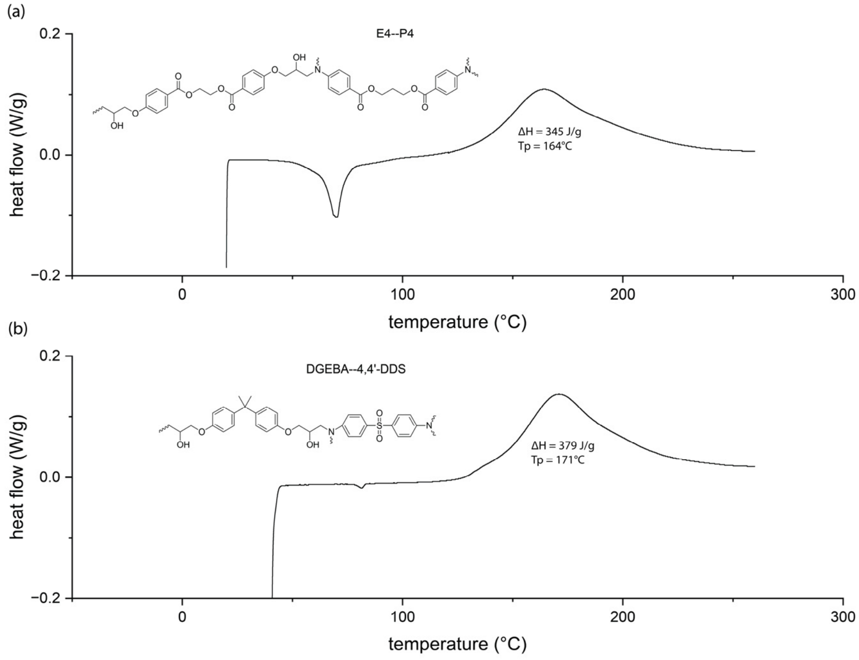

Figure S37. DSC traces for polymerization of (a) a representative epoxy-amine derived polyester and (b) DGEBA-4,4'-DDS networks for comparison of polymerization enthalpy (ΔH) and peak temperature (Tp)

Table S1. *Extent of cure information obtained by comparison of initial and residual polymerization enthalpies for polyester and reference systems.*						
**network identification**	**cure enthalpy, round 1 (J/g)**	**cure enthalpy, round 2 (J/g)**	**cure enthalpy, round 3 (J/g)**	**residual enthalpy, round 1 (J/g)**	**residual enthalpy, round 2 (J/g)**	**calculated extent of cure (%)**
DGEBA-4,4'-DDM	505.84	435.35	506.02	7.1104	10.744	98.149
DGEBA-4,4'-DDS	379.09	423.13	387.90	19.245	16.966	95.436
Epon828-4,4'-DDS	444.82	444.82	432.94	16.635	13.798	96.548
DGEBA--3,3'-DDS	429.18	376.71	464.72	18.137	15.874	95.985
ASYM4--ASYM4	318.12	305.66	363.30	23.448	17.314	93.806
E4--E4	333.12	336.47	339.82	12.749	15.662	95.778
E4--P4	345.76	366.48	417.95	14.113	11.845	96.555
P4--P4	381.69	401.97	356.68	21.187	15.907	95.121
E4--E3	480.04	397.78	355.30	20.587	15.098	95.659
E3--E3	418.22	336.09	382.52	19.596	18.638	94.955

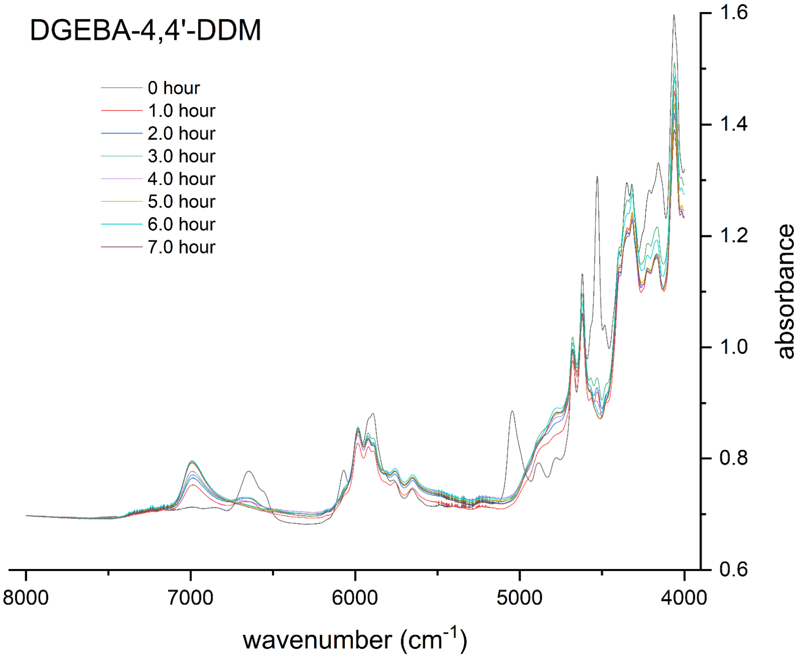

Figure S38. Overlaid NIR spectra of DGEBA-4,4'-DDM throughout cure profile.

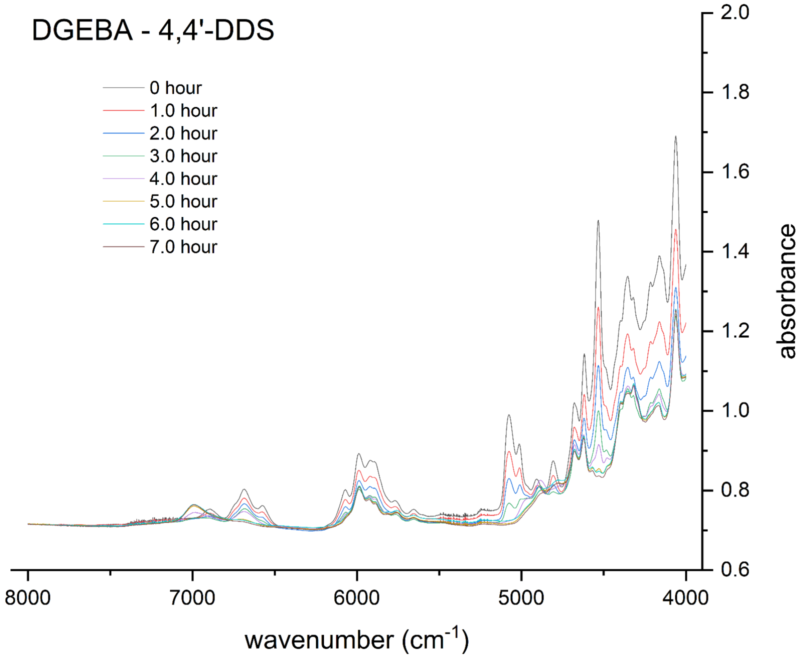

Figure S39. Overlaid NIR spectra of DGEBA-4,4'-DDS throughout cure profile.

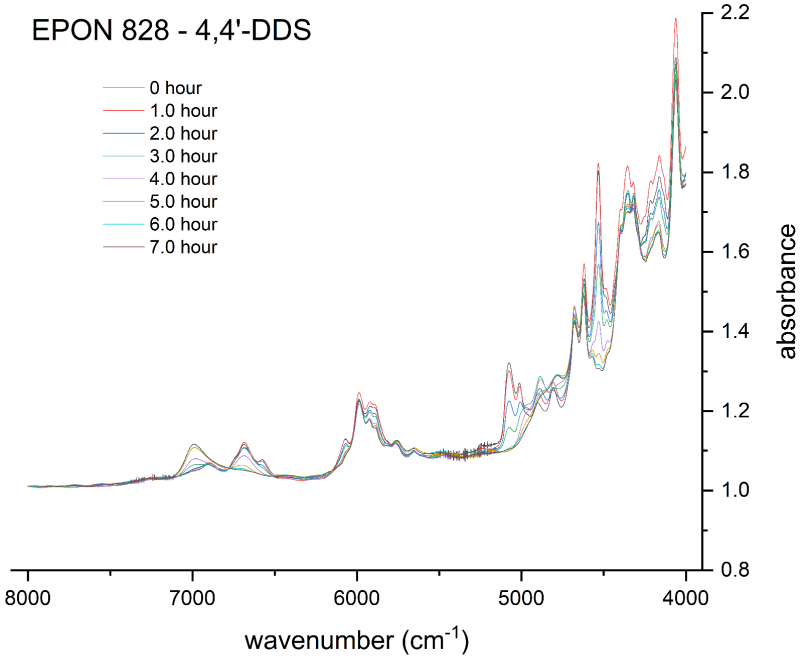

Figure S40. Overlaid NIR spectra of Epon 828-4,4'-DDS throughout cure profile.

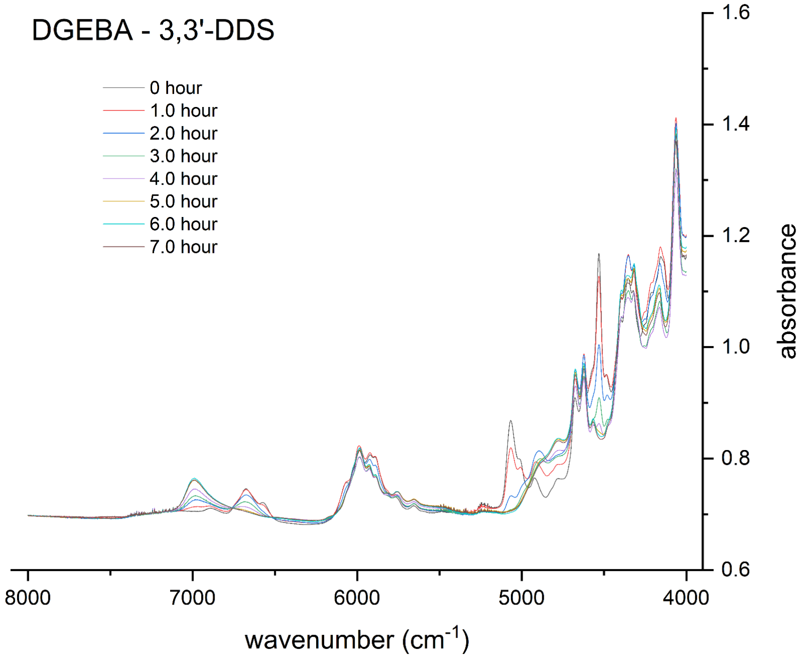

Figure S41. Overlaid NIR spectra of DGEBA-3,3'-DDS throughout cure profile.

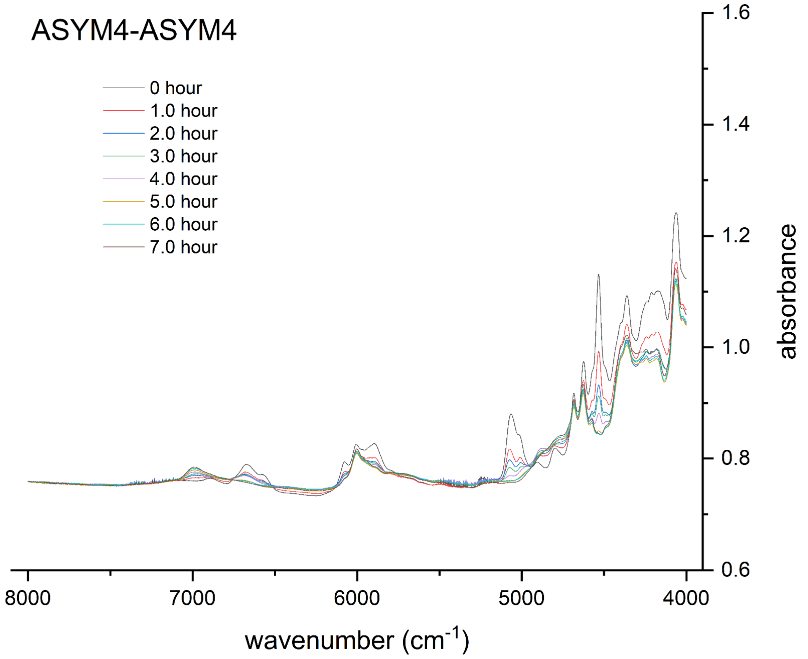

Figure S42. Overlaid NIR spectra of ASYM4-ASYM4 throughout cure profile.

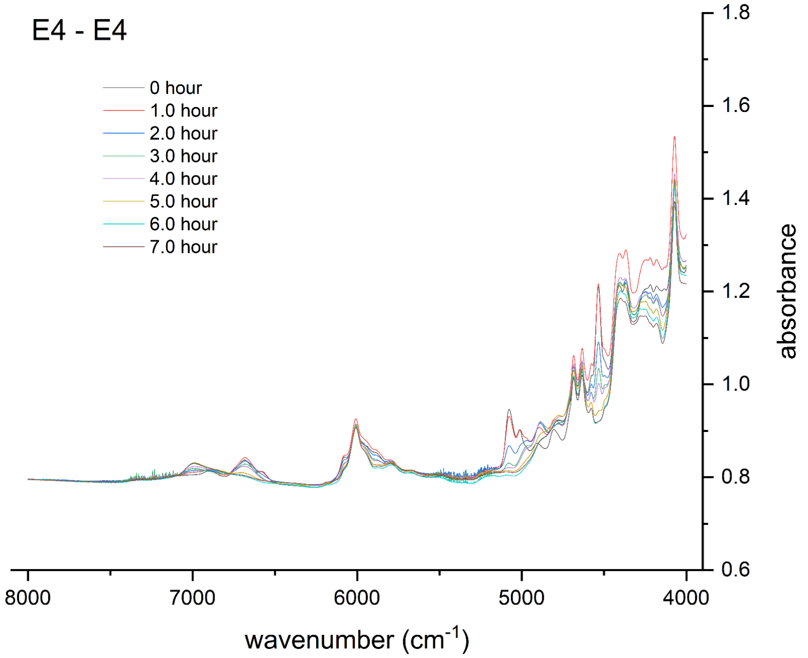

Figure S43. Overlaid NIR spectra of E4-E4 throughout cure profile.

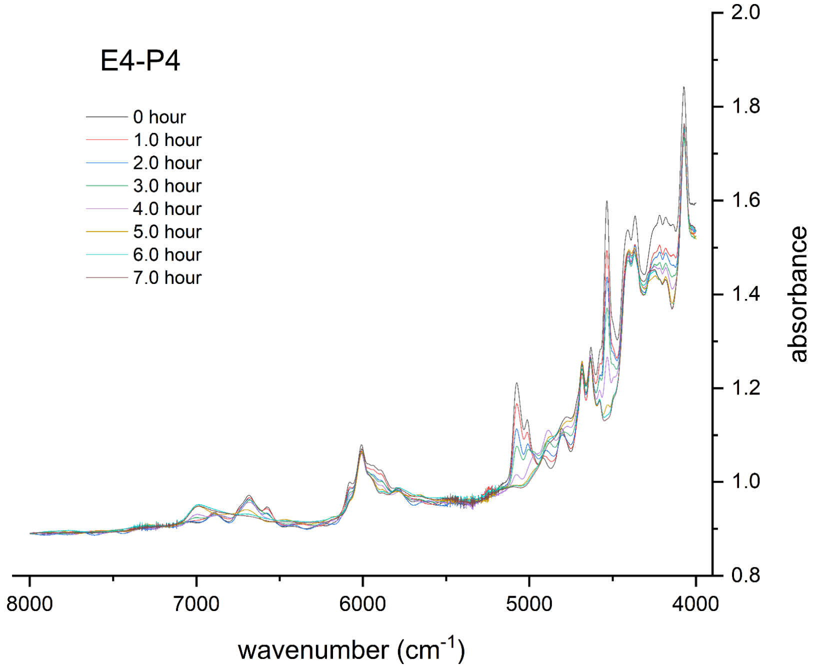

Figure S44. Overlaid NIR spectra of E4-P4 throughout cure profile.

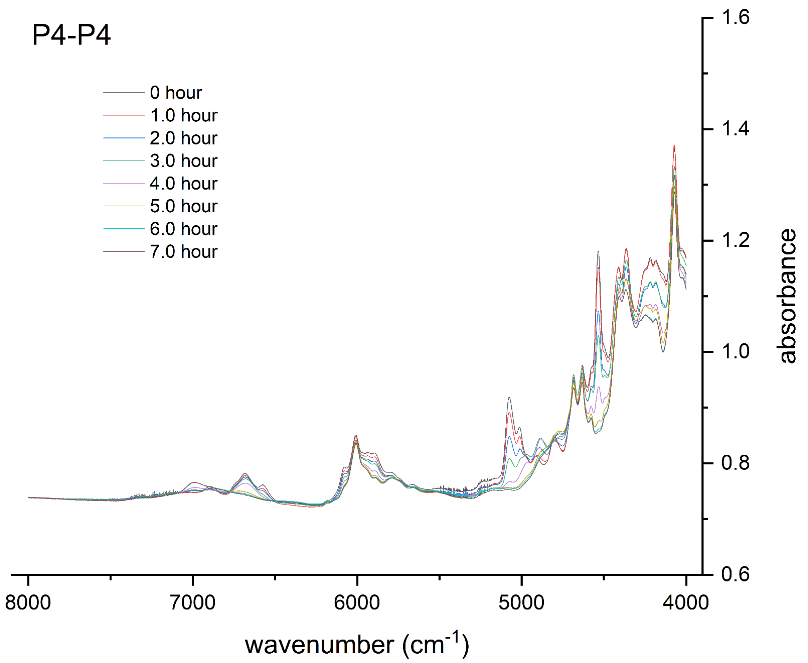

Figure S45. Overlaid NIR spectra of P4-P4 throughout cure profile.

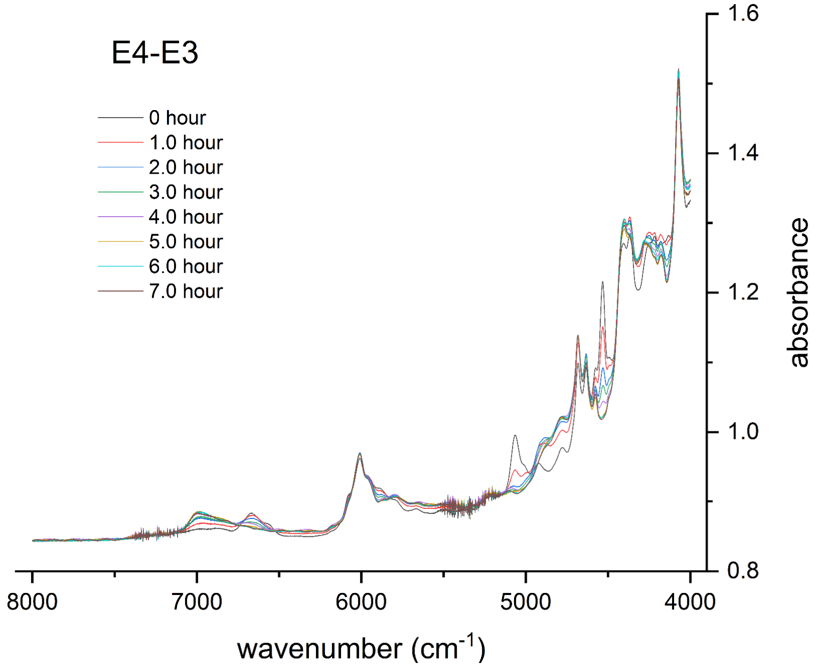

Figure S46. Overlaid NIR spectra of E4-E3 throughout cure profile.

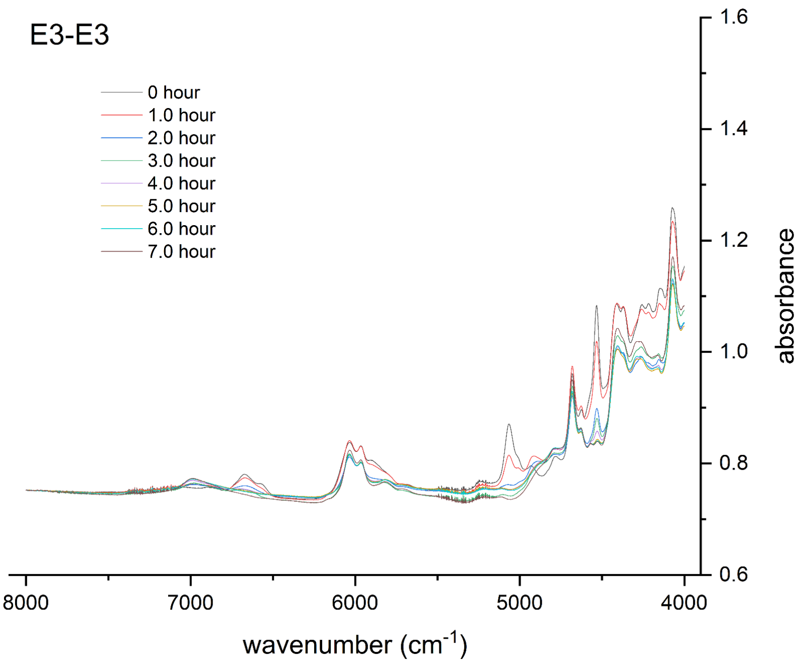

Figure S47. Overlaid NIR spectra of E3-E3 throughout cure profile.

**Table S2.** Integral values for secondary relaxation peak(s) from tan δ curves.		
network	integral value	peak maximum (°C)
DGEBA - 4,4'-DDS	4.95 ± 0.16	-40.5 ± 7.1
Epon 828 - 4,4'-DDS	4.89 ± 0.21	-36.4 ± 0.4
DGEBA - 3,3'-DDS	3.59 ± 0.15 *	-43.9 ± 5.6, +71.3 ± 6.7
P4-P4	4.63 ± 0.09	-35.8 ± 2.4
E4-P4	5.50 ± 0.15	-34.6 ± 2.1
E4-E4	5.83 ± 0.16	-29.1 ± 0.4
E4-E3	4.63 ± 0.09	-44.1 ± 0.4
E3-E3	2.28 ± 0.25	-54.1 ± 3.6
*cumulative integral value for both peaks observed		

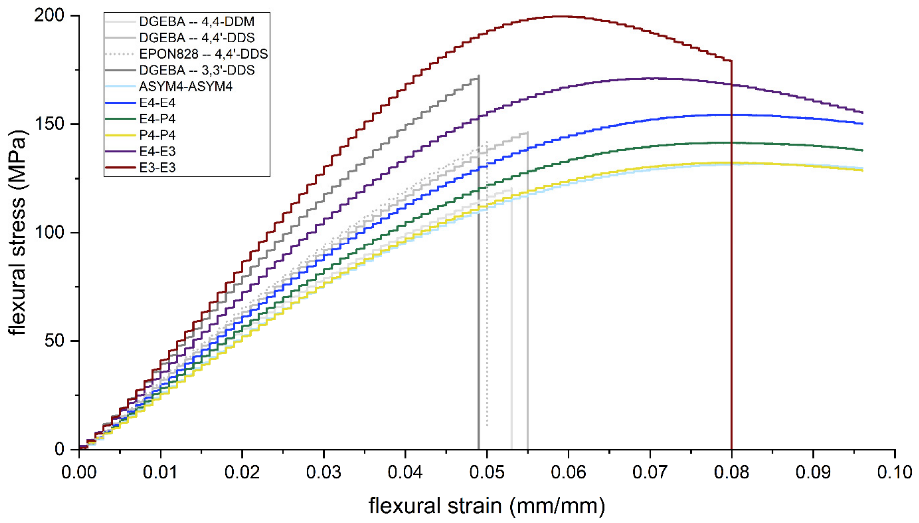

Figure S48. Representative flexural stress-strain curves for polyester and reference networks.

Table S3. Overview of polyester and reference network densities (obtained by Archimedes principle), component molar masses, theoretical molecular weight between crosslinks, and crosslink density

network	ρ (g/ cm^3)	molar mass of diepoxide (g/mol)	molar mass of diamine (g/mol)	diepoxide strand molar mass	diamine strand molar mass	calculated average molecular weight between crosslink points (M_c) **	υ theoretical maximum crosslink density (mol crosslinks per cm^3 network) ***
DGEBA -- 4,4'-DDM	1.2002	340.42	198.27	342.42	196.27	294	0.00272
DGEBA -- 4,4'-DDS	1.2423	340.42	248.3	342.42	246.3	310	0.00267
EPON828 -- 4,4'-DDS	1.2421	374*	248.3	376	246.3	333	0.00249
DGEBA -- 3,3'-DDS	1.2438	340.42	248.3	342.42	246.3	310	0.00267
ASYM4-ASYM4	1.2742	370.4	256.31	372.4	254.31	333	0.00255
E4-E4	1.3144	414.41	300.31	416.41	298.31	377	0.00232
E4-P4	1.3061	414.41	314.34	416.41	312.34	382	0.00228
P4-P4	1.2991	428.44	314.34	430.44	312.34	391	0.00222
E4-E3	1.3161	414.41	300.31	416.41	298.31	377	0.00233
E3-E3	1.3193	414.41	300.31	416.41	298.31	377	0.00233

*molecular weight defined by 2x EEW, **weighted as 2/3 of network strands are epoxy-derived, 1/3 are amine-derived, ***calculated from Mc as described by Levita et al. [1]and Chang et al.[2]: nitrogens are the crosslink points for 3 network strands and each strand is shared by two crosslink points – so crosslink density = $(3/2\ M_c)^{-1}$

Table S4. Overview of polyester and reference network functional group equivalent weights.

network	molecular weight of repeat unit* (g/mol)	hydroxyl groups per repeat unit**	calculated hydroxyl equivalent weight (g/mol hydroxyl)	ester groups per repeat unit	calculated ester equivalent weight (g/mol ester)	calculated hydroxyl concentration (mol/cm^3)	calculated ester concentration (mol/cm^3)
DGEBA—4,4'-DDM	879.11	4	220	0	N/A	0.00546	N/A
DGEBA—4,4'-DDS	929.14	4	232	0	N/A	0.00535	N/A
EPON 828—4,4'-DDS ***	1056.50	_	245	0	N/A	0.00507	N/A
DGEBA—3,3'-DDS	929.14	4	232	0	N/A	0.00535	N/A
ASYM4--ASYM4	997.11	4	249	3	332	0.00511	0.00383
E4--E4	1129.13	4	282	6	188	0.00466	0.00698
E4--P4	1142.16	4	286	6	190	0.00457	0.00686
P4--P4	1171.22	4	293	6	195	0.00444	0.00666
E4--E3	1129.13	4	282	6	188	0.00466	0.00699
E3--E3	1129.13	4	282	6	188	0.00467	0.00701

*repeat unit derived from 2 equivalents of diepoxide and 1 equivalent of diamine
**2 hydroxyl group integral to repeat unit, 4 additional hydroxyl groups shared at termini of repeat units (2 + ½(4) = 4),
For example: A network formed from 200 diepoxide molecules and 100 diamine molecules would form 400 potential hydroxyl groups and have 100 repeat units as defined
***weighted average based on n = 0.17 (EEW = 187 g/eq, MW = 374 g/mol)

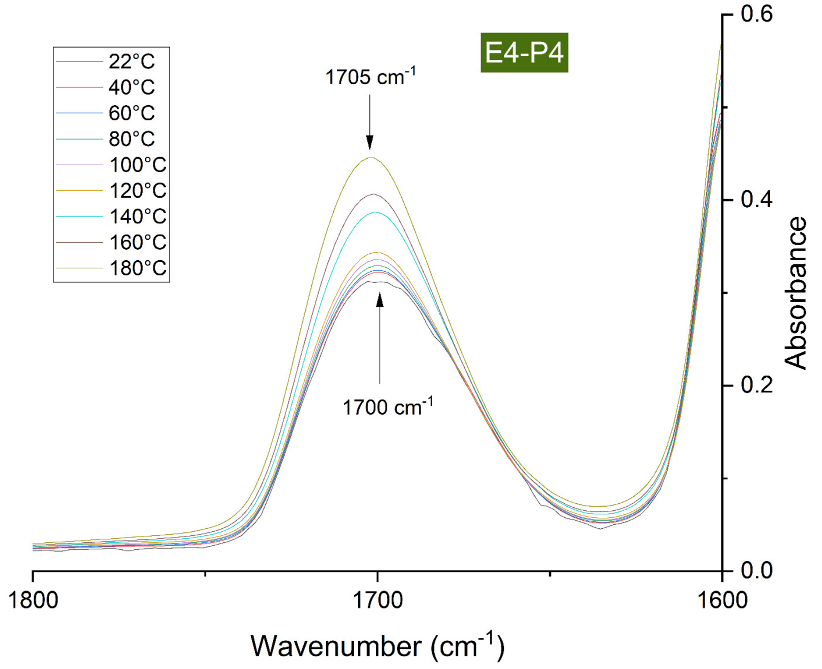

Figure S49. Overlaid mid-IR spectra (ambient to 180°C) of E4-P4 network (C=O stretch region).

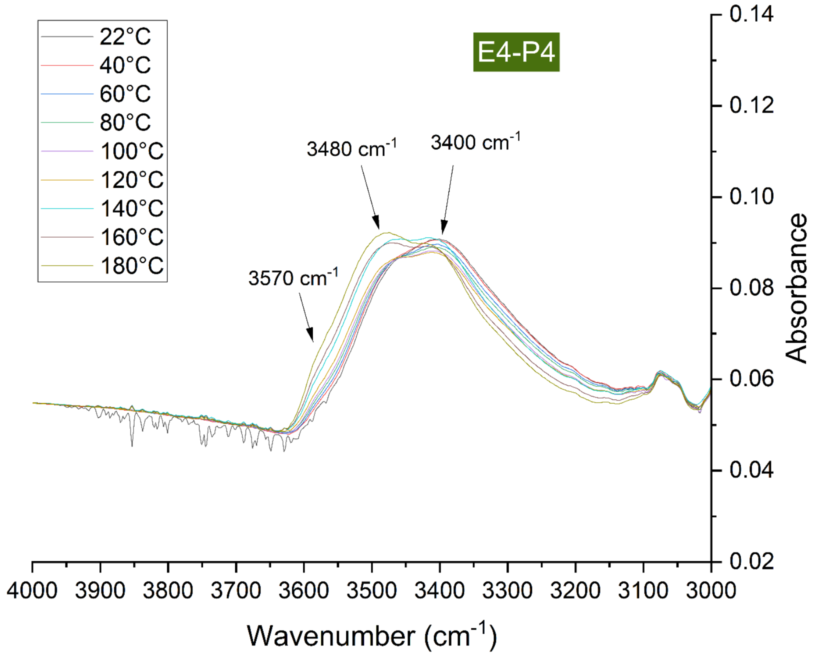

Figure S50. Overlaid mid-IR spectra (ambient to 180°C) of E4-P4 network (OH stretch region).

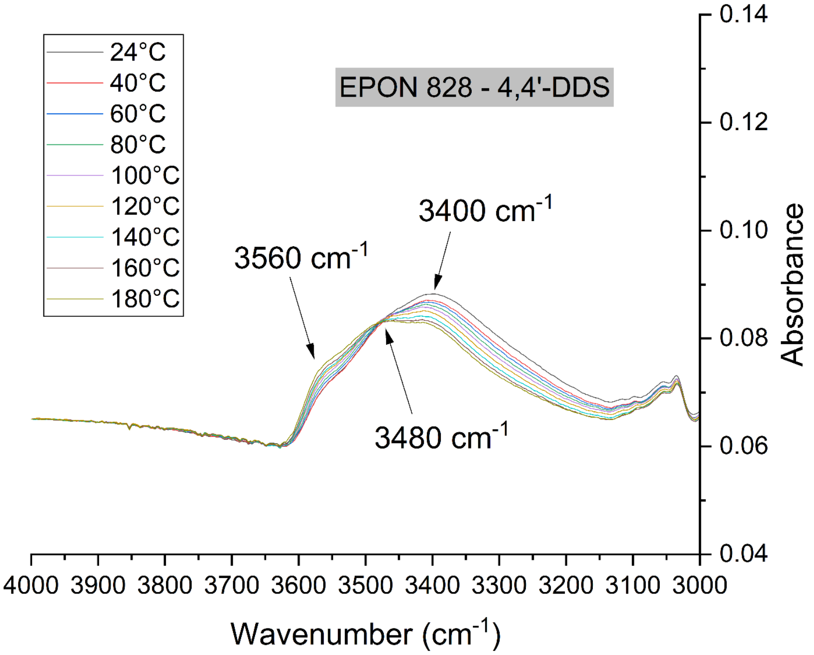

Figure S51. Overlaid mid-IR spectra (ambient to 180°C) of Epon 828 – 4,4'-DDS network (OH stretch region).

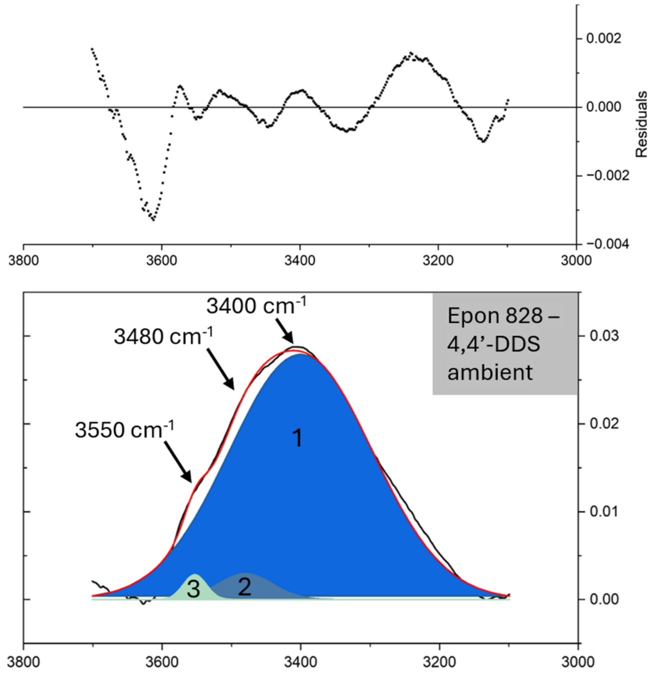

Fitting Results

Peak Index	Peak Type	Area Intg	FWHM	Max Height	Center Grvty	Area IntgP
1	Gaussian	7.18271	242.71624	0.0279	3400	94.66289
2	Gaussian	0.28782	91.716	0.00295	3480	3.79326
3	Gaussian	0.11714	39.09251	0.00282	3552.87574	1.54385

Figure S52. Peak fits and residuals for OH stretch region at ambient temperature, Epon 828 – 4,4'-DDS

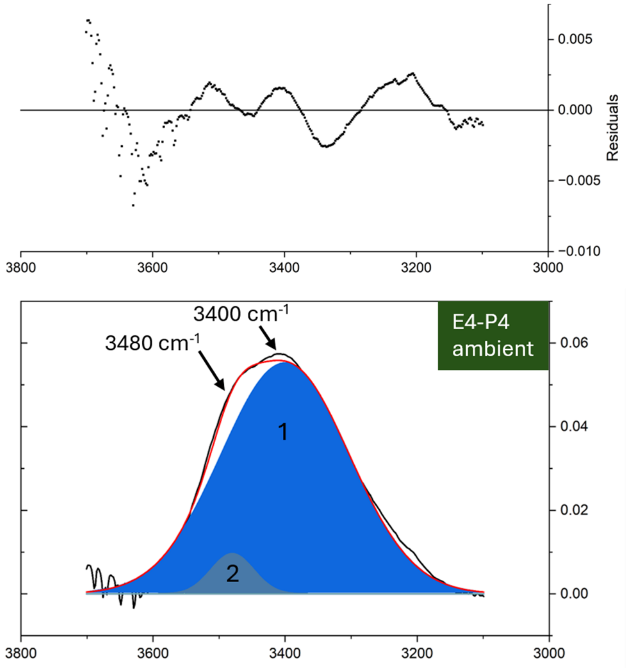

Fitting Results

Peak Index	Peak Type	Area Intg	FWHM	Max Height	Center Grvty	Area IntgP
1	Gaussian	13.35206	227.46621	0.05525	3400	94.50605
2	Gaussian	0.7762	75.80324	0.00962	3480	5.49395
3	Gaussian	0	0.00402	2.02222E-4	3552.68919	0

Figure S53. Peak fits and residuals for OH stretch region at ambient temperature, E4-P4

(1) Levita, G.; De Petris, S.; Marchetti, A.; Lazzeri, A. Crosslink density and fracture toughness of epoxy resins. *Journal of Materials Science* **1991**, *26* (9), 2348-2352. DOI: 10.1007/BF01130180.
(2) Chang, T. D.; Carr, S. H.; Brittain, J. O. Studies of epoxy resin systems: Part B: Effect of crosslinking on the physical properties of an epoxy resin. *Polymer Engineering & Science* **1982**, *22* (18), 1213-1220. DOI: https://doi.org/10.1002/pen.760221807 (acccessed 2025/10/20).
